# Proof-of-concept study of an at-home, engaging, digital intervention for pediatric ADHD

**DOI:** 10.1371/journal.pone.0189749

**Published:** 2018-01-11

**Authors:** Naomi O. Davis, Jeffrey Bower, Scott H. Kollins

**Affiliations:** 1 Department of Psychiatry and Behavioral Sciences, School of Medicine, Duke University, Durham, North Carolina, United States of America; 2 Akili Interactive Labs, Inc., Boston, MA, United States of America; Karolinska Institutet, SWEDEN

## Abstract

**Objective:**

Pharmacological and behavioral therapies have limited impact on the distinct neurocognitive impairments associated with ADHD, and existing cognitive training programs have shown limited efficacy. This proof-of-concept study assessed treatment acceptability and explored outcomes for a novel digital treatment targeting cognitive processes implicated in ADHD.

**Method:**

Participants included 40 children with ADHD and 40 children without ADHD. Following psychiatric screening, ADHD ratings, and baseline neuropsychological measures, participants completed 28-days of at-home treatment. Neuropsychological assessment was repeated at end-of-study along with treatment satisfaction measures.

**Results:**

Eighty-four percent of treatment sessions were completed and ratings showed strong intervention appeal. Significant improvements were observed on a computerized attention task for the ADHD group and a highly impaired ADHD High Severity subgroup. There was no change for the non-ADHD group. Spatial working memory also improved for the ADHD group and the ADHD High Severity subgroup.

**Conclusion:**

Findings provide preliminary support that this treatment may improve attention, working memory, and inhibition in children with ADHD. Future research requires larger-scale randomized controlled trials that also evaluate treatment impact on functional impairments.

**Trial registration:**

ClinicalTrials.gov NCT01943539

## Introduction

Attention Deficit/Hyperactivity Disorder (ADHD) is a neurodevelopmental disorder characterized by symptoms of inattention, hyperactivity, and impulsivity that are elevated relative to developmental level and pervasive across settings and over time, often persisting from childhood into adulthood [1]. Although ADHD is diagnosed by examining behavioral symptoms and associated functional impairments, the disorder is associated with distinct structural and functional brain differences that subserve neurocognitive functioning [2, 3], including executive functioning, working memory, processing efficiency, as well as motivational systems that impact intrinsic motivation and sensitivity to rewards/punishments [4, 5]. These challenges to cognitive control influence the behavioral manifestations of ADHD and constitute key potential treatment targets.

Current evidence-based treatments for ADHD include medication and behavioral therapies involving the patient and/or family members [6, 7]. Despite significant research documenting its benefits, pharmacological treatment has limitations including unfavorable side effects for some patients [8], lack of normalization even in well-titrated doses [9], and lack of benefit when medication is not taken. Recent evidence also suggests that pharmacological treatment may not show optimal benefits in some neurocognitive domains [10, 11]. With respect to behavioral therapy, parent behavior management training and cognitive behavioral skills training for adolescent and adults with ADHD are effective treatments, in particular when provided in conjunction with medication, and optimal outcomes may result when behavioral therapy is initiated prior to pharmacological treatment [12]. However, effects of behavioral therapy are often not maintained outside the treatment period, treatment can be difficult to access, and a recent meta-analysis raised questions about its efficacy for improving ADHD symptoms [13, 14]. Critically, adherence and compliance to both pharmacological and behavioral treatment regimens is often quite poor [15].

Given the chronicity of ADHD and the challenges of achieving sustained treatment efficacy over time, the potential for directly impacting ADHD impairments through direct cognitive training has garnered widespread interest [16]. Indeed, current evidence-based treatments were not explicitly designed to address the underlying pathophysiology that is associated with ADHD, which may explain some of the limitations of existing pharmacological and behavioral treatments [17]. An array of cognitive training programs has been promoted to address the specific cognitive processes that are impaired in ADHD. To date, however, despite promising findings from individual studies of such methods, equivocal findings from meta-analyses have been reported, such as inadequate generalizability of effects outside of training and the limitations of addressing cognitive processes versus core symptoms [18–20]. Lack of effects may also be attributed to the wide range of treatment targets, which may not address neurocognitive mechanisms that are specifically associated with impairment in ADHD [17].

Cognitive neuroscience research has the potential to inform the development of novel interventions for specific disorders, such as ADHD, by linking implicated brain systems with specifically-targeted digital interventions, which can be optimally designed to operate within game interfaces [21]. In a series of studies, Gazzaley and colleagues identified a critical neural process underlying cognitive control ability, and demonstrated that a game-like cognitive intervention built to specifically target this system can achieve cognitive remediation [22, 23]. Their therapeutic approach for cognitive remediation relied on improving management of “cognitive interference,” which occurs when two or more tasks or goals compete for cognitive resources within the executive function system. Susceptibility to cognitive interference is a core limitation to cognitive control ability and underlies cognitive deficits in sustained attention, impulsivity and working memory. Susceptibility to cognitive interference was measured by comparing performance when tasks are performed in isolation to when they are performed simultaneously with other tasks. The team designed a video game-like computerized treatment deployed in a home setting, called *Neuroracer*, to measure and, over time, reduce susceptibility to cognitive interference among a population of older adults. Results of the study indicated that interference susceptibility was reduced and demonstrated that cognitive benefits transferred to performance on untrained tests of attention, impulsivity, and multitasking [23]. In addition, they showed that changes in EEG signals occurred at neurological loci associated with cognitive control after completing the prescribed therapeutic regimen. Improvements in multi-tasking were maintained in the treatment group after 6 months of no treatment, and this maintenance effect also correlated with the same EEG-based neurological changes.

Given that these cognitive processes–difficulty with sustained attention, impulsivity, and working memory–are similarly implicated in ADHD, the current study was designed to directly explore the feasibility of using an advanced version of the digital interference treatment with this population. Designed to achieve acceptability in this population, as well as to enable at-home engagement and high-compliance with the treatment regimen, *Project*: *EVO* (EVO) is a digital treatment based on the Neuroracer model that deploys the interference-based cognitive control-targeting mechanics in a high-quality action video game-like interface. The intervention was developed using high-quality graphics and reward loops designed to be engaging for children. Additionally, the intervention embeds algorithms that continuously adapt to cognitive control ability and provide feedback on progress and compliance.

A multi-site research program was undertaken to examine the cognitive mechanics of the EVO platform across ADHD and non-ADHD children to assess feasibility in multiple pediatric populations. Here, we report on the feasibility, safety, and effects of the EVO adaptive treatment as an at-home, non-physician-administered intervention in this population. Specifically, the objectives were: 1) To demonstrate treatment acceptability in an at-home intervention protocol, specifically addressing compliance with the protocol and qualitative feedback at the end of the study period; and 2) To explore whether participants demonstrate improvements in cognitive function (e.g., objective measures of attention and working memory) following the intervention period.

## Methods

### Participants

**Fig 1:**
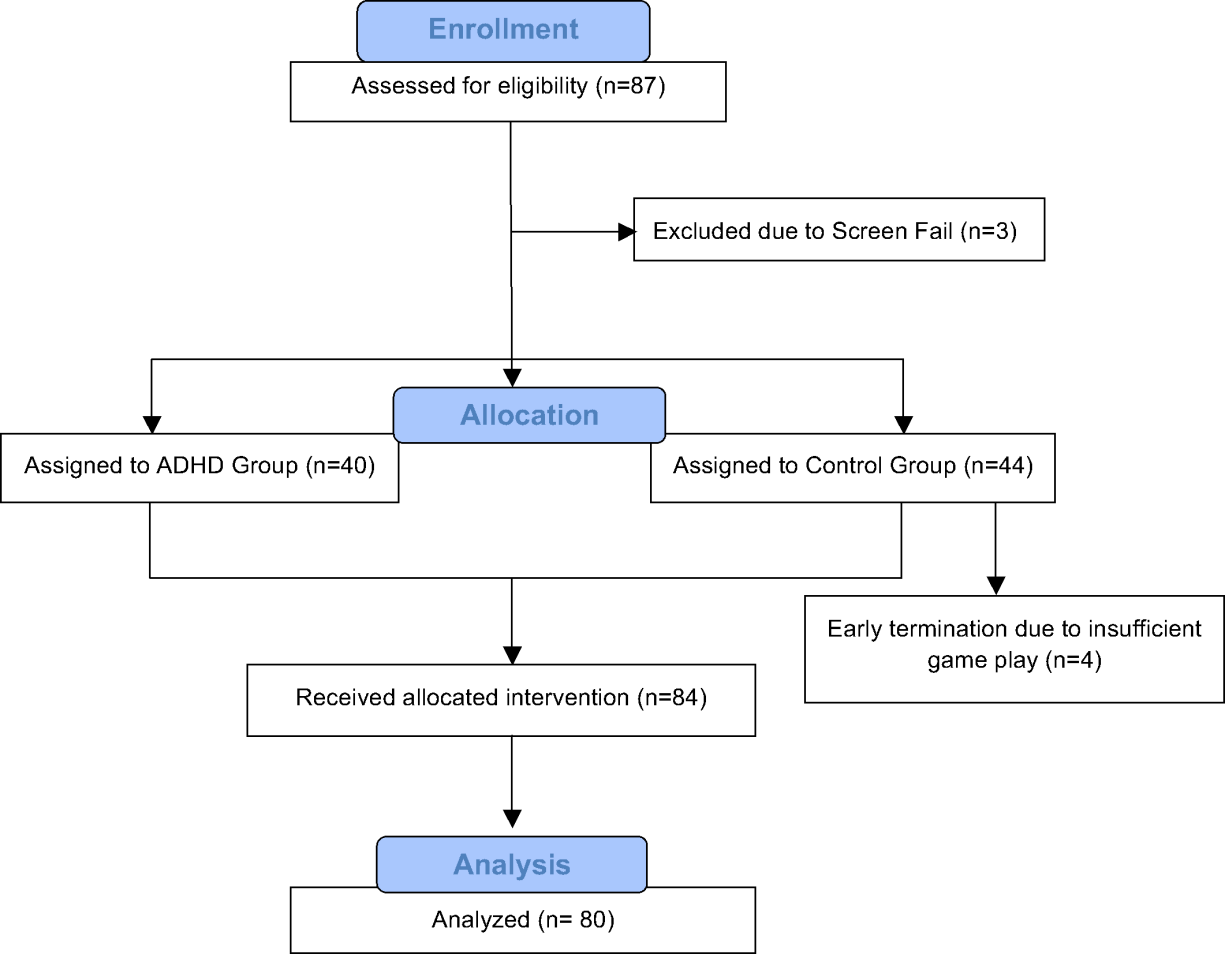
CONSORT flow diagram.

Participants were self-referred and were recruited from 3 U.S. sites (i.e., one university setting and two clinical research centers) between January 2014 and August 2014. Data collection was completed on-site. Participants included 40 children with a confirmed diagnosis of ADHD (*M* = 10.3 years) and 40 age-matched children without ADHD or any psychiatric disorder (*M* = 10.5 years) (Table 1). Eligible participants were between the ages of 8–12 years and were able to follow verbal and written instructions in English. All participants were, in the opinion of the investigators, functioning within the normal range of intellectual functioning. Eligibility criteria for the ADHD cohort included the following: 1) confirmed DSM-IV diagnosis of ADHD (any subtype); 2) baseline ADHD-RS-IV score > = 24 (completed via a clinician-administered semi-structured interview with the participant’s parent); and 3) off psychotropic medication to treat ADHD (i.e., stimulant medication) for at least one week prior to study enrollment. Participants who had previously taken medication were expected to have discontinued the use of ADHD medication prior to study recruitment and were not asked or encouraged to discontinue their ADHD treatment in order to participate in the study. Eligibility criteria for the non-ADHD cohort included an ADHD-RS-IV score < = 13. For both cohorts, additional eligibility criteria required the following: 1) consistently off any other psychotropic medication for the prior month; 2) no comorbid psychiatric diagnosis that was uncontrolled or may have confounded the study objectives, as assessed via the MINI-KID; 3) not entering or exiting behavioral therapy within the prior four weeks; and 4) no history of failure to respond to an adequate trial of two ADHD treatments. Written consent was obtained from parents/caregivers of all participants prior to completing study procedures. Given the open-label design, participants and study staff were not blinded to study condition. This study was reviewed and approved by the Copernicus Group IRB on 8/28/2013 (IRB Tracking #: AKI1-13-296). See Fig 1 for the CONSORT flow diagram.

**Table 1:** Baseline demographics and measures for the ADHD, ADHD High Severity subgroup, and non-ADHD groups.

Characteristic	Group	N (Male/Female)	Mean	SD	P Value ^a^
Age	ADHD	40 (24/16)	10.35	1.24	-
	ADHD High Severity Subgroup	22 (14/8)	10.2	1.26	0.66
	non-ADHD	40 (21/19)	10.54	1.49	0.55
Baseline TOVA API	ADHD	40 (24/16)	-4.06	3.57	-
	ADHD High Severity Subgroup	22 (14/8)	-5.93	3.32	0.043
	non-ADHD	40 (21/19)	-1.24	2.66	< 0.0001
Baseline ADHD-RS	ADHD	40 (24/16)	35.88	8.85	-
	ADHD High Severity Subgroup	22 (14/8)	39.36	6.4	0.08
	non-ADHD	40 (21/19)	4.03	3.25	< 0.0001

^a^ P values are from a t-test (Between-Subjects, 2-Tailed) comparing the indicated group to the ADHD group.

A subset of participants from the ADHD group was subsequently selected via post-hoc statistical analyses to comprise the ADHD High Severity group. As described further below and characterized in Table 1, this subset of participants demonstrated a greater response to the intervention. The ADHD High Severity group was characterized by a baseline TOVA API score of less than or equal to -1.8 and an ADHD-RS score of greater than or equal to 30. Twenty-two of the 40 ADHD participants met these criteria.

### Measures

#### Screening and selection measures

MINI International Neuropsychiatric Interview for Children and Adolescents (MINI-KID) [24]. The MINI-KID is a structured diagnostic interview that assesses psychiatric disorders in children and adolescents. It has demonstrated concordance to other structured psychiatric interviews and can be implemented using parent report. For the present study, the MINI-KID was used to establish ADHD diagnosis and to rule out other exclusionary psychopathology.

ADHD-Rating Scale-4^th^ edition (ADHD-RS-IV) [25]. The ADHD-RS is an 18-item validated scale that is completed via semi-structured interview with the participant’s parent to assess symptom severity. Each item corresponds to one of the DSM-IV diagnostic criteria for ADHD and is rated on a 4-point scale (0–3) based on frequency of symptoms, resulting in a range of scores from 0–54, with higher scores reflecting more significant symptomatology.

Columbia Suicide Severity Rating Scale (C-SSRS) [26]. The C-SSRS is a brief interview instrument that is used to assess suicidal ideation and suicidal behavior including severity, intensity, types of behaviors, and potential lethality of behaviors. It has been well-validated for use in clinical trials.

#### Safety and feasibility measures

Adverse events. Parents were instructed to report any adverse events by phone to the study staff during the study period, and any adverse events that were spontaneously reported during study visits were recorded.

Compliance. The number of treatment sessions completed at home was divided by the number of prescribed sessions to calculate the proportion of required intervention sessions completed for each participant.

Questionnaire on intervention appeal. Questionnaires regarding treatment experience and satisfaction were administered at Screening (Day 0) and Post-Intervention (Day 28) visits and were used to assess product acceptance and tolerability. Parents completed a 14-item paper questionnaire about their child’s experience, and study participants responded to 16 questions that were administered orally by study staff. Both Questionnaires were a mix of Likert scales, multiple choice, and open-ended questions. Descriptive statistics were calculated for Likert items while frequency of responses was totaled for multiple choice and open-ended questions.

#### Neurocognitive/attentional outcomes

Test of Variables of Attention (TOVA, version 8) [27]. The TOVA is a computerized continuous performance test that objectively measures attention and impulsivity, comprising a target stimulus and a nontarget stimulus that are presented individually and randomly based on a predetermined ratio. Participants are instructed to press a button immediately after seeing a target but not to respond when a nontarget is presented. The TOVA provides numerous summary statistics of performance (e.g., average reaction time, reaction time standard deviation, omission errors, and commission errors) and generates an Attention Performance Index (API) score. The API is a composite of z-scores for reaction time, reaction time variability, and *d’* (sensitivity index) and includes scores typically ranging from -10 to 10. The threshold for typical performance on the API is set at zero, with most typically developing individuals performing above zero (i.e., between 0–10). The API score was used as the primary TOVA outcome for this study. In addition, all of the TOVA standardized scores were also examined as well as Exgaussian TAU, which is an experimental measure that is sensitive to the long reaction times that are observed among individuals with ADHD.

Behavior Rating Inventory of Executive Function (BRIEF)–Parent Form [28]. The BRIEF is an 86-item parent-completed questionnaire that assesses executive functioning behaviors of children in everyday life. Behaviors are rated on a 3-point scale based on frequency. The BRIEF’s eight subscales are aggregated into two domains (Metacognition and Behavioral Regulation) and also yield one overall score (Global Executive Composite). Given the hypothesis that the intervention would directly affect executive measures in the attention, impulsivity, and working memory domains, we analyzed the Working Memory subscale (Metacognition domain) and the Inhibit subscale (Behavioral Regulation domain). Additional analyses were also conducted with the available summary scores (i.e., Metacognition, Behavioral Regulation, Global Executive Composite).

Cambridge Neuropsychological Test Automated Battery (CANTAB) is a computer-based cognitive assessment that measures neuropsychological functioning [29, 30]. The CANTAB cognitive tests used in this study were selected from tests that were relevant to ADHD and provide information on different cognitive domains fitting with our intended cognitive targets, including Spatial Working Memory (SWM), Rapid Visual Processing (RVP), and Delayed Match to Sample (DMS).

### Procedure

**Fig 2:**
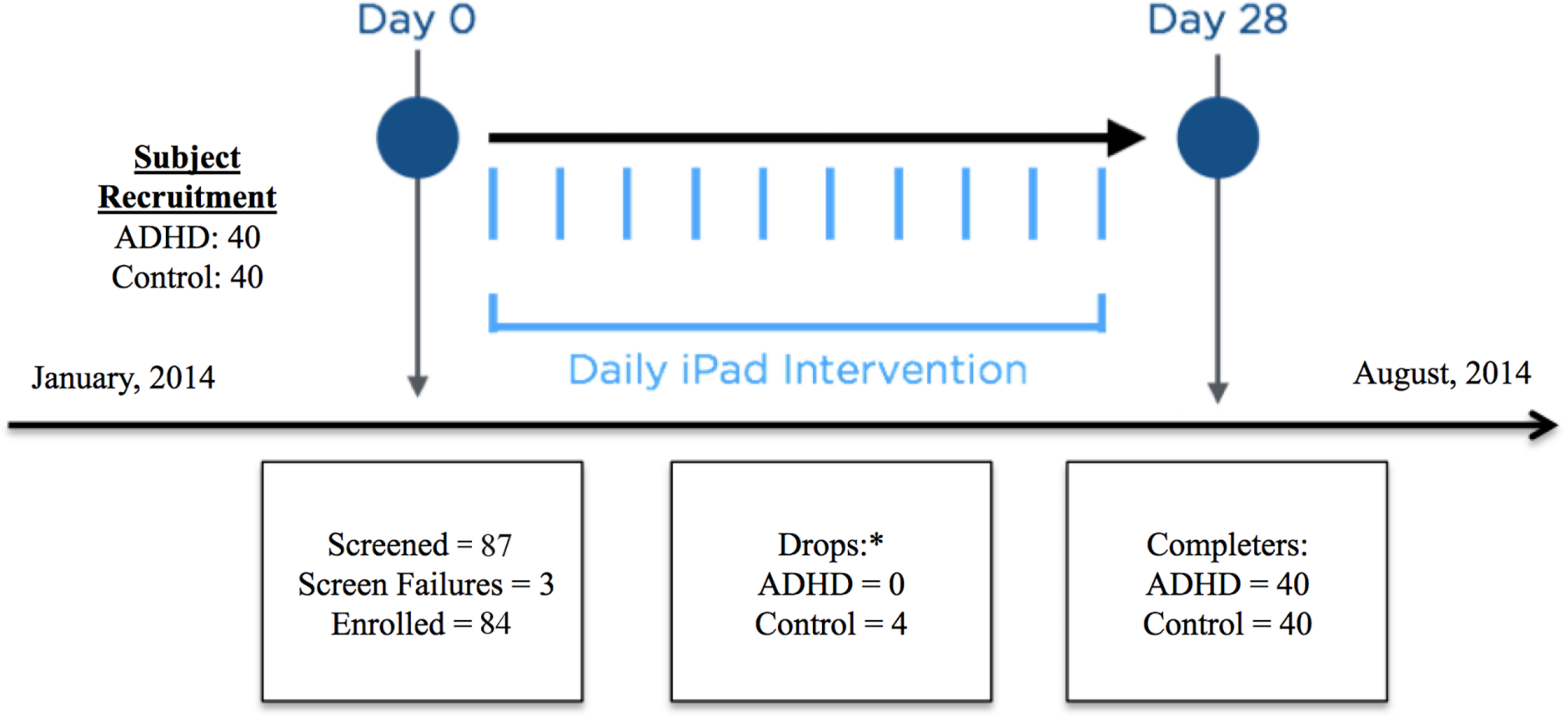
Study design and participant flow. *All participants drop outs were due to noncompliance with the intervention.

The study involved three phases: a screening visit, an intervention period, and a post-intervention visit (Fig 2). The screening visit (Day 0) was conducted to determine study eligibility and to collect baseline data before initiating the intervention period. Eligibility was determined by administration of the MINI-KID to establish or rule out a diagnosis of ADHD and other psychiatric conditions, and to determine the severity of any presenting ADHD symptoms. Following psychiatric screening, eligible participants completed laboratory baseline assessments (CANTAB, TOVA). After a short break, participants were instructed to engage with EVO for approximately 20 minutes to establish baseline performance and ensure that participants understood how to use with the intervention properly. This period included one EVO practice session, one diagnostic/assessment session, and one treatment session. Following EVO use, study staff recorded observations about the child’s experience, and throughout the session any adverse events were captured by study staff. At the end of the screening visit, parents were given instructions for the at-home intervention period.

During the intervention period (Days 1–27) participants were instructed to use the intervention at-home for approximately 30–45 minutes a day, 5 days a week. Each daily treatment period was accomplished by completing 7 consecutive EVO sessions, which consisted of either a four-minute dual-task intervention segment or an automatically triggered ten-minute dual-task evaluation segment that was used to recalibrate the cognitive skills difficulty for the next level. On each day of the intervention, EVO counts the number of sessions as they are completed and limits use for the remainder of any given day following 7 completed sessions. Compliance with treatment was monitored electronically and the parents of participants were notified by email if there was failure to use the intervention over a 48-hour period. Parents were also given a reminder phone call on day 21 to notify them that their child was entering the final week of the study.

Participants returned to the clinic on Day 28 for a final evaluation. Post-intervention data that were collected included the cognitive assessments (CANTAB, TOVA) and additional EVO cognitive metric and interaction data, as well as parent-completed questionnaires.

### Digital treatment

The *Project*: *EVO* platform was designed to incorporate the fundamental features of *Neuroracer* [23] into a state-of-the-art mobile digital intervention platform that participants engage with through an action video game interface that deploys modern videogame graphics, engaging reward loops, and real-time adaptive mechanics to dynamically personalize difficulty based on the user’s ability. *Project*: *EVO* employs a perceptual discrimination attention/memory task as well as a continuous visuomotor “driving” task. Performance is assessed on each task in isolation and when performed together to calculate a performance index for each individual user. A personalized multi-task intervention regimen is automatically configured and delivered to the user, and is optimized adaptively across both tasks to constantly increase multi-task performance. As patients/participants proceed through the treatment, periodic re-calibration occurs to maintain an optimal difficulty level. In addition, the mechanics are coded into a proprietary framework that utilizes real-time and between-session adaptation in order to tailor the treatment specifically to an individual automatically, without the need for any configuration or tuning by a clinician, thus allowing it to be deployed entirely remotely in the patient’s home. This tailoring ensures that patients are consistently performing at a predefined level of difficulty that is challenging but also tolerable because of inherently rewarding videogame-like interaction mechanics.

### Statistical analysis

Intervention safety and acceptability were examined by analyzing EVO data on compliance with intervention use, adverse events, and parent and child satisfaction ratings.

Analyses were also conducted to examine all cognitive/behavioral outcome measures that were administered in the pre- and post-intervention visits. Analyses were run separately for the ADHD and non-ADHD groups. The means of the pre-test scores were compared to the means of the post-test scores using a 2-tailed paired T-test. For the main neurocognitive outcome, TOVA API, a Shapiro-Wilk test for normality was performed. If the distribution of scores was found to deviate from normality at an alpha of less than 0.05, an equivalent Wilcoxon sign rank test was performed instead of a T-test. Due to the exploratory nature of these analyses, all results less than an alpha of 0.05 were considered statistically significant. For completeness we also report if the alpha levels meet significance under a Bonferroni correction within a statistical family. The outcome measures from the TOVA, CANTAB Spatial Working Memory, CANTAB Rapid Visual Processing, CANTAB Delayed Match to Sample, and BRIEF each measure distinct aspects of executive function and each will be treated as independent statistical families for Bonferroni adjustments. All analyses were conducted in the open source statistical package, R (v. 3.3.1). Only participants who completed the at-home period and completed the final assessments were included in the analysis, therefore no missing data was present. Completing the at-home period was defined by completion of 50% of the required treatment sessions. Treatment session data was uploaded electronically through WI-FI and processed through a cloud-based Application Programming Interface (API) to create a daily report to clinical site staff on participant treatment compliance. Clinical staff were instructed to reach out to the participants if there was no documented usage of the intervention for more than 24 hours. Participants that were dropped or did not complete the study were replaced and thus only participants without protocol deviations (a Per-Protocol analysis set) were included in analyses. Four participants from the non-ADHD group were dropped from the study for poor compliance, which represents a replacement rate of 5% of the total study final sample size. An Intent To Treat (ITT) analysis was not considered necessary due to the low drop rate.

In addition to these planned analyses, post-hoc analyses were also conducted to examine outcomes for the subset of the ADHD group with most impairment as defined by greater functional deficits and lower attentional performance. A cut-point analysis was performed [31] to find the optimal cut-points for baseline ADHD-RS and TOVA API scores that would define a subgroup of ADHD participants that showed maximum change on the TOVA API. Sub-samples were selected at combinations of baseline scores (cut-points) for the ADHD-RS (ADHD-RS score range, 24 to 41) with TOVA API (TOVA API score range -6 to 1). The results of this analysis indicated that the greatest change on TOVA API was observed with a sub-sample that had an ADHD-RS baseline score of > = 30 and a TOVA API < -1.8. This sub-sample had 22 participants (mean age = 10.2, 14 males and 8 females), and did not differ in terms of age or gender.

Given the exploratory nature of the study, the sample size was chosen to be sufficiently large to detect significant within subject effects of at least 0.45 (Cohen’s D) for a 2-Tailed test, at anlpha-0.05 with a power of 80%.

## Results

### Participant flow

A total of 87 participants were screened for the study and a total of 3 participants were screen fails. During the intervention period, four participants from the non-ADHD group were dropped from the study for non-compliance with the at-home intervention use. These participants were replaced by recruiting additional participants. All of the ADHD group participants were compliant with the intervention and none of these participants were replaced during the study. A total of 40 ADHD and 40 non-ADHD participants completed the study. See Fig 2 for a graphical representation of the participant flow through the study.

### Safety and acceptability

Across all participants, 84% of all prescribed in-home sessions were completed. This is equal to approximately 10.9 hours of total intervention time per child across the approximately 28-day intervention period. The ADHD group completed an average of 81% (10.8 hours of intervention), the ADHD High Severity subgroup completed an average of 86% (11.1 hours of intervention), while the non-ADHD group completed an average of 87% (11.5 hours of intervention) of the required at-home sessions. There were no significant differences found between the ADHD and non-ADHD groups (p = 0.20), the ADHD and ADHD High Severity subgroup (p = 0.38), or the non-ADHD and the ADHD High Severity subgroup (p = 0.30). Compliance was calculated based on number of exact days at home with the intervention, which varied slightly between groups based on each child’s exit visit.

A total of 9 adverse events were reported over all study phases; however, none of those adverse events were judged by the PIs and site staff as related to EVO (Table 2). None of the adverse events were reported during the practice session at the Screening Visit. Additionally, there were only 4 participants who dropped out of the study, and none of these participants were in the ADHD cohort.

**Table 2:** At-home adverse event descriptions, severity, and relationship to intervention for the ADHD and non-ADHD groups.

Group	Event description	At-home Adverse Event Severity	Relationship to Intervention
ADHD			
	Toothache	Mild	Unrelated
	Headache, moderate severity, 1 day	Moderate	Unrelated
	Viral gastroenteritis	Moderate	Unrelated
	Headache, 1 day	Moderate	Unlikely
non-ADHD			
	Viral gastroenteritis	Mild	Unrelated
	Upper respiratory	Mild	Unrelated
	Foot pain	Mild	Unrelated
	Influenza	Moderate	Unrelated
	Reflux esophagitis	Severe	Unrelated

Results from the questionnaire on intervention appeal showed an average participant-reported enjoyment rating for EVO treatment of 6.9 on a 1–10 scale (ADHD *M* = 6.93; ADHD High Severity subgroup *M* = 7.1; non-ADHD *M* = 6.85). Parent ratings of their child’s enjoyment showed a similar pattern of overall enjoyment (ADHD *M* = 6.1; non-ADHD *M* = 6.6). See Table 3 for additional intervention appeal ratings.

**Table 3:** Intervention appeal questions per parent and child ratings.

	ADHD	ADHD High Severity subgroup	non-ADHD
	*Mean(SD)*	*Mean(SD)*	*Mean(SD)*
*Child Intervention Appeal Questions*	* *	* *	* *
In terms of enjoyment how would you rate playing EVO? (1 = Boring, 10 = Fun)	6.9(2.3)	7.1(2.3)	6.9(2.3)
How challenging was EVO to play? (1 = Easy, 10 = Hard)	6.6(2.3)	6.3(2.3)	6.3(2.4)
*Parent Intervention Appeal Questions*			
In terms of enjoyment how would you rate playing EVO? (1 = Boring, 10 = Fun)	6.1(2.4)	6.3(2.4)	6.6(2.1)
How challenging was EVO to play? (1 = Easy, 10 = Hard)	6.8(2.3)	6.6(2.6)	6.3(1.9)
How would you rate the time your child spent playing EVO?	*Percent*	*Percent*	*Percent*
Very Worthwhile	30%	27%	23%
Somewhat Worthwhile	50%	50%	67%
Waste of Time	10%	10%	5%
No Answer	10%	13%	5%
If EVO was available to you after this study, would you want him/her to play it more?			
Yes	58%	64%	53%
No	40%	36%	43%
I Don't Know	0%	0%	4%
No Answer	2%	0%	0%

### Neurocognitive outcomes

#### Attentional functioning

**Fig 3:**
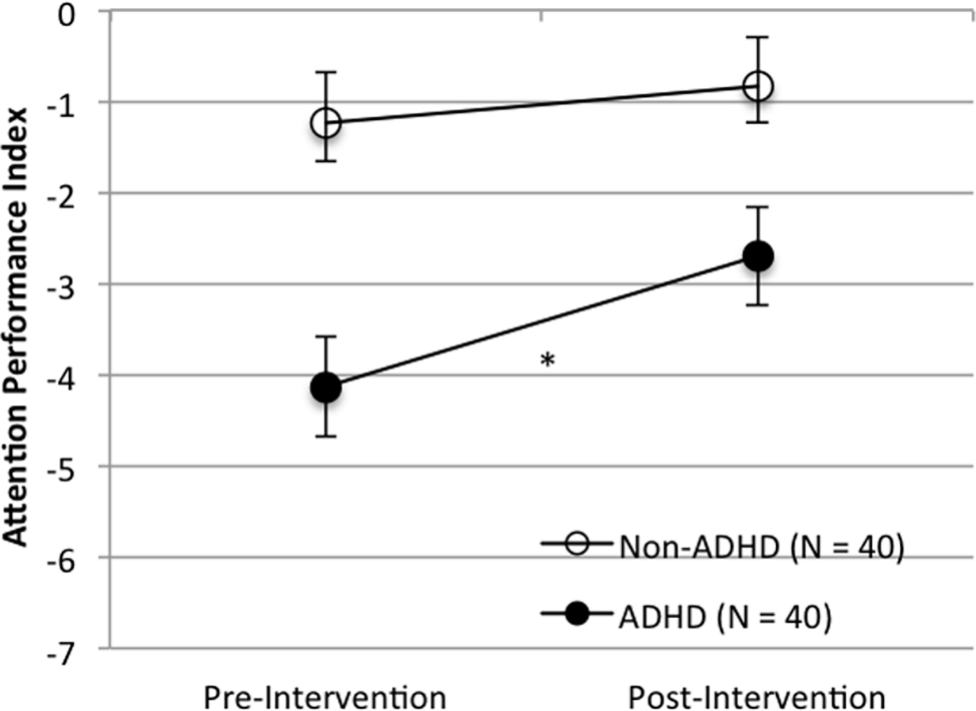
Performance on the TOVA API pre- and post-intervention for the ADHD group and the non-ADHD group. Note: Error bars represent standard error of the mean (*p < 0.05; ** p < 0.005).

**Fig 4:**
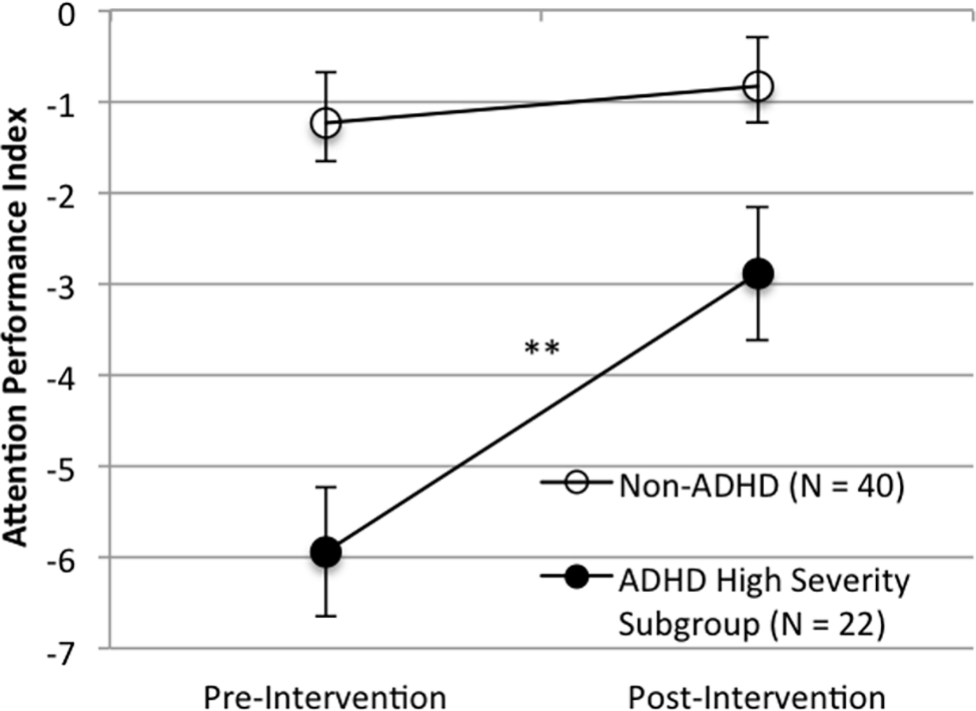
Performance on the TOVA API pre- and post-intervention for the ADHD High Severity subgroup and the non-ADHD group. Note: Error bars represent standard error of the mean (*p < 0.05; ** p < 0.005).

Significant improvements were observed on the TOVA primary composite measure, the Attention Performance Index (API) in the ADHD group (T-Test; *N* = 40, *t* = 2.21, *p* = 0.033, Effect Size (d) = 0.35, mean of differences = -1.43, 95%CI-Lower = -2.75, 95%CI-Upper = -0.12). In addition, a larger effect of the intervention was observed for the subset of ADHD participants with greater functional deficits and lower attentional performance (T-Test; *N* = 22, *t* = 3.33, *p* = 0.003, Effect Size (d) = 0.71, mean of differences = -3.05, 95%CI-Lower = -4.96, 95%CI-Upper = -1.15). There was no change in API scores for the non-ADHD group (T-Test; *N* = 40, *t* = 1.04, *p* = 0.30, Effect Size (d) = 0.17, mean of differences = -0.39, 95%CI-Lower = -1.16, 95%CI-Upper = 0.37). See Figs 3 and 4 for API results.

Several other standardized score measurements from the TOVA were examined. The ADHD High Severity subgroup, but not the full ADHD group, showed significant improvements on two indicators of ADHD severity: Reaction Time Mean Standard Score (T-Test; *N* = 22, *t* = 3.03, *p* = 0.006, Effect Size (d) = 0.65, mean of differences = -13.16, 95%CI-Lower = -22.19, 95%CI-Upper = —4.13), and Reaction Time Variability (Variance) Standard Score (T-Test; *N* = 22, *t* = 2.89, *p* = 0.009, Effect Size (d) = 0.62, mean of differences = -26.42, 95%CI-Lower = -45.42 95%CI-Upper = -7.43). Both the ADHD group (Wilcoxon; *N* = 40, *z* = 2.39, *p* = 0.016, Effect Size (r) = 0.38, mean of differences = 53.27, 95%CI-Lower = 3.34, 95%CI-Upper = 46.02) and the non-ADHD group (Wilcoxon; *N* = 40, *z* = 2.05, *p* = 0.040, Effect Size (r) = 0.32, mean of differences = 40.27, 95%CI-Lower = 0.32, 95%CI-Upper = 33.33) showed a decline in Omission Error Standard Score (i.e., missing targets). D-prime, a measure of signal detection sensitivity, remained relatively constant in both the ADHD group (*p* = 0.718) and the ADHD High Severity subgroup (*p* = 0.406). Analyses of the ExGaussian Tau, an experimental measure derived from the TOVA that may be a sensitive metric for attentional challenges, indicated significant improvement for both the ADHD group (T-Test; *N* = 40, *t* = 2.14, *p* = 0.039, Effect Size (d) = 0.34, mean of differences = 30.56, 95%CI-Lower = 1.7, 95%CI-Upper = 59.43) and the ADHD High Severity subgroup (T-Test; *N* = 22, *t* = 3.21, *p* = 0.004, Effect Size (d) = 0.69, mean of differences = 64.77, 95%CI-Lower = 22.85, 95%CI-Upper = 106.7) A complete table listing of TOVA standardized score results with Bonferroni corrections reported is found in S1 Table.

#### Working memory and other cognitive outcomes

**Fig 5:**
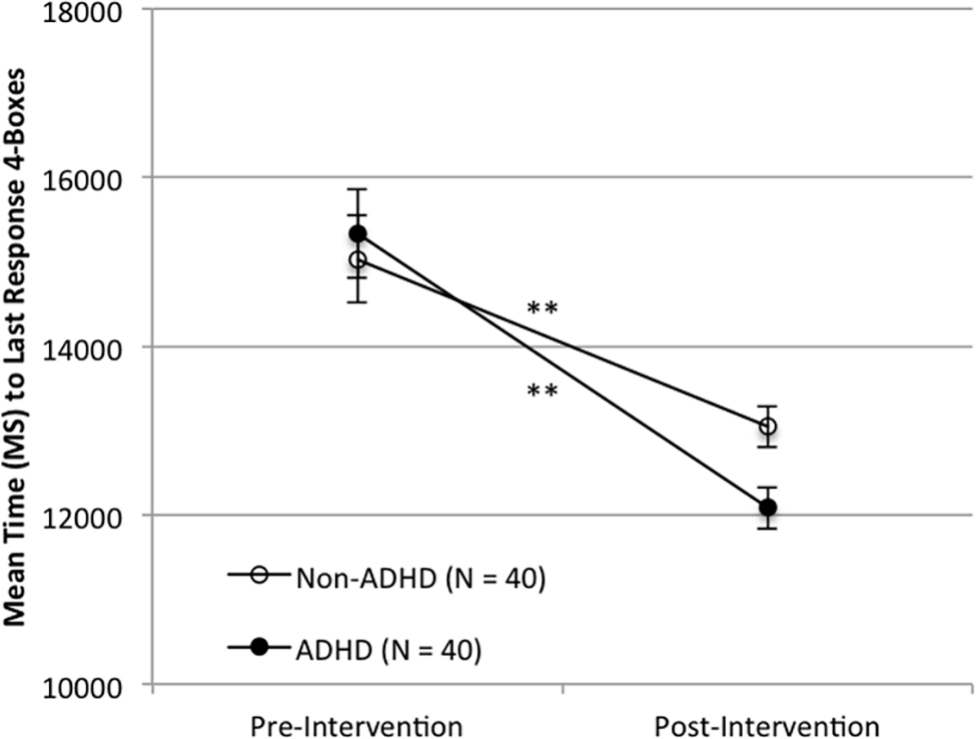
Performance on the spatial working memory task pre- and post-intervention for the ADHD group and the non-ADHD group. Note. Scores represent mean time to last response with 4 boxes. Error bars represent standard error of the mean. (** p < 0.005).

**Fig 6:**
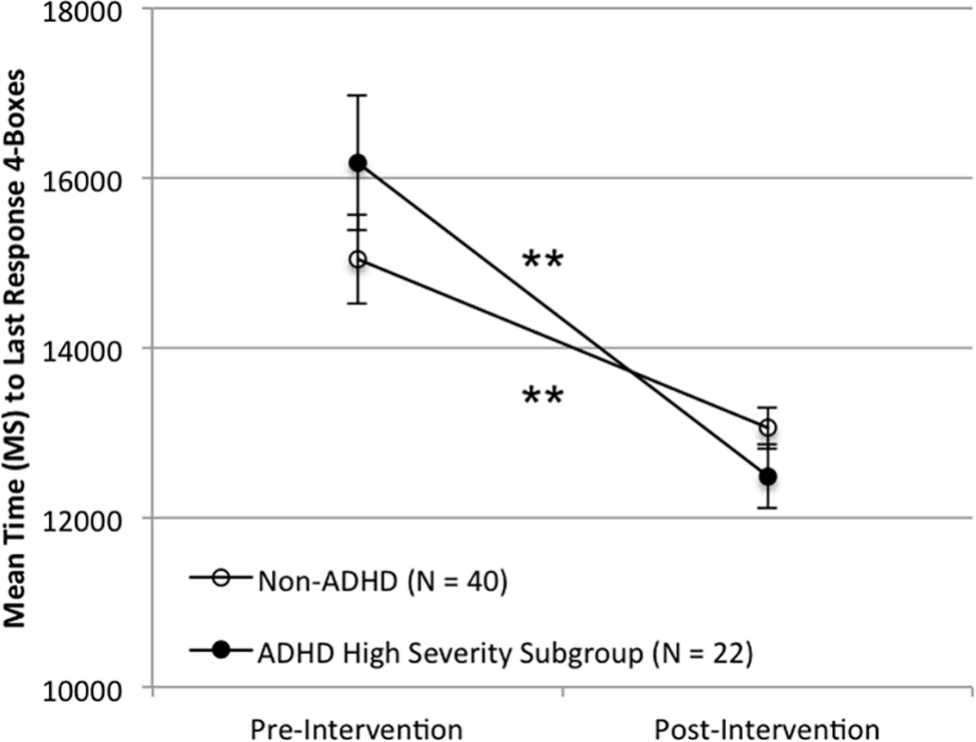
Performance on the spatial working memory task pre- and post-intervention for the ADHD High Severity subgroup and the non-ADHD group. Note. Scores represent mean time to last response with 4 boxes. Error bars represent standard error of the mean. (** p < 0.005).

The ADHD group showed significant improvement (p < 0.05) on 8 of 12 variables within the CANTAB Spatial Working Memory (SWM) test, 3 of 10 variables within the Rapid Visual Processing (RVP) test, and 0 of 16 within the Delayed Match to Sample (DMS) test. Figs 5 and 6 demonstrate one such measure for demonstration purposes. The ADHD High Severity subgroup showed significant improvement (p < 0.05) on 8 of 12 variables within the SWM, 0 of 10 variables within the RVP, and 1 of 16 within the DMS. The non-ADHD group showed significant improvement (p < 0.05) on 5 of 12 variables within the SWM, 6 of 10 variables within the RVP, and 9 of 16 within the DMS. Overall, the results suggest an improvement for Spatial Working Memory (SWM) for the ADHD and ADHD High Severity subgroup and well as improvements for the non-ADHD group on Rapid Visual Processing (RVP) and Delayed Match to Sample (DMS). Please see S2–S4 Tables for a complete table listing of these CANTAB results with Bonferroni corrections reported.

#### Executive functioning

On the Working Memory subscale, percentile scores for the ADHD group improved non-significantly but a trend-level effect was observed (Wilcoxon; *N* = 40, *z* = 1.81, *p* = 0.07, Effect Size (r) = 0.29, mean of differences = 2.48, 95%CI-Lower = 0.0, 95%CI-Upper = 5.5) (Table 4). For the ADHD High Severity subgroup, percentile scores on Working Memory (Wilcoxon; *N* = 22, *z* = 2.40, *p* = 0.014, Effect Size (r) = 0.51, mean of differences = 3.23, 95%CI-Lower = 1, 95%CI-Upper = 7.5) and Inhibition (Wilcoxon; *N* = 22, *z* = 2.19, *p* = 0.027, Effect Size (r) = 0.47, mean of differences = 5.68, 95%CI-Lower = 1, 95%CI-Upper = 15.5) improved significantly. The Working Memory and Inhibit scores did not change significantly for the non-ADHD group. In addition, the BRIEF summary scores (i.e., Metacognition, Behavioral Regulation, Global Executive Composite) did not change significantly over time for any of the groups.

**Table 4:** Parent BRIEF pre-post scores for the ADHD, ADHD High Severity subgroup, and non-ADHD groups.

Outcome	Group	N	Mean-Pre (SD)	Mean-Post(SD)	Test Statistic (T/Z)	P Value	Effect Size d(r)	95% CI (U|L)
Inhibit Percentile	ADHD	40	86.15(17.848)	85.1(16.105)	0.678(Z)	0.504	0.086(0.107)	(-3.5|7.5)
	ADHD High Severity Subgroup	22	92.5(7.812)	86.818(14.447)*	2.187(Z)	0.027	0.584(0.466)	(1|15.5)
	non-ADHD	40	46.1(21.534)	45.125(21.731)	0.402(T)	0.69	0.064	(-3.929|5.879)
Working Memory Percentile	ADHD	40	94.45(6.891)	91.975(9.178)	1.81(Z)	0.07	0.392(0.286)	(0|5.5)
	ADHD High Severity Subgroup	22	95.591(4.982)	92.364(8.375)**	2.404(Z)	0.014	0.475(0.513)	(1|7.5)
	non-ADHD	40	37.475(21.748)	37.25(22.15)	0.085(T)	0.933	0.013	(-5.137|5.587)
Global Executive Composite Percentile	ADHD	40	90(9.894)	87.65(14.107)	0.243(Z)	0.812	0.209(0.038)	(-2.5|5)
	ADHD Subgroup	22	90.955(9.368)	88.636(15.035)	0.199(Z)	0.853	0.206(0.042)	(-3|10.5)
	non-ADHD	40	27.525(24.286)	26.975(22.955)	0.221(T)	0.827	0.035	(-4.492|5.592)
Metacognitive Index Percentile	ADHD	40	78.575(26.06)	81.525(18.4)	-0.612(Z)	0.546	0.097	(-7|2.5)
	ADHD High Severity Subgroup	22	84.318(19.759)	82.136(21.565	0.995(Z)	0.329	0.212	(-2|7)
	non-ADHD	40	32.175(26.521)	30.7(24.154)	0.533(T)	0.597	0.084	(-4.122|7.072)
Behavior Regulation Index	ADHD	40	90.475(10.696)	87.975(14.508)	0.919(Z)	0.363	0.145	(-1.5|4)
	ADHD High Severity Subgroup	22	92.227(8.793)	89.136(14.721)	0.674(Z)	0.513	0.144	(-2.5|11.5)
	non-ADHD	40	28.025(22.342)	28.975(23.456)	0.215(Z)	0.834	0.034	(-5.5|5.5)

* indicates statistical significance at an alpha of 0.05 (2-tailed) for pre- to post-intervention difference within group.

** indicates statistical significance after a Bonferroni correction of 0.05/5 = 0.01.

For each variable and group, the normality assumption for T-Tests was verified using a Shapiro-Wilks test. If the Shapiro-Wilks test indicated that the distribution of scores did not meet normality, a Wilcoxon rank sum test was performed instead. In the Test Statistic column this is indicated by a (T) or (Z) after the test statistic indicating if a T-Test (T) or a Wilcoxon test (Z) was performed. P values were calculated according to the statistical test run. Effect sizes are Cohen’s d with rank-sum correlation in parentheses if appropriate.

## Discussion

The primary aim of this study was to explore the feasibility of using an at-home digital intervention with children diagnosed with ADHD. *Project*: *EVO* was well-tolerated by children with ADHD, was feasibly deployed in the home setting over a treatment period of four weeks, and resulted in positive ratings of acceptability by both parents and children. Notably, there was high compliance and no dropouts in the ADHD cohort. The lack of dropouts was particularly notable considering the time burden required, the fact that no physician or clinician was directly involved in the treatment protocol, and documented dropout rates of >10% in traditional treatment trials as well as real-world settings [15, 32]. Given the risks that must be considered regarding other treatment options for ADHD, this information is of high importance as a first step in developing innovative treatment for this chronic disorder. Specifically, given the high demands on parents’ time and resources, it is critical that available treatments for ADHD can be reasonably incorporated into a daily routine, utilized consistently by children with ADHD, and most critically implemented without the need for a physician, therapist, or psychologist oversight. Furthermore, research on treatment acceptability suggests high acceptability ratings for behavioral as compared to pharmacological treatment [33, 34], yet access to behavioral treatments is limited when interventions can only be given by trained providers in a clinic setting.

Findings from the current study provide preliminary support that this digital therapy intervention may be effective for improving attention, working memory, and inhibition in pediatric ADHD, especially among children with greater symptom severity and impaired attention. As shown in prior work with Neuroracer, the platform upon which *Project*: *EVO* was developed, participants who received the digital intervention demonstrated improvements in attentional functioning and spatial working memory as measured by accepted computerized tests over the one-month intervention period. These improvements were observed on TOVA’s composite Attention Performance Index and hallmark sub-component metrics such as reaction time and reaction time variability (variance), as well as a range of metrics on the CANTAB spatial working memory test. Interestingly, the ADHD groups’ improved Attention Performance index and maintenance of D-prime, despite a decrease in the omission error standard score, may imply attentional improvements that accompany and are not compromised by a shift to a more cautious/withholding response profile.

**Fig 7:**
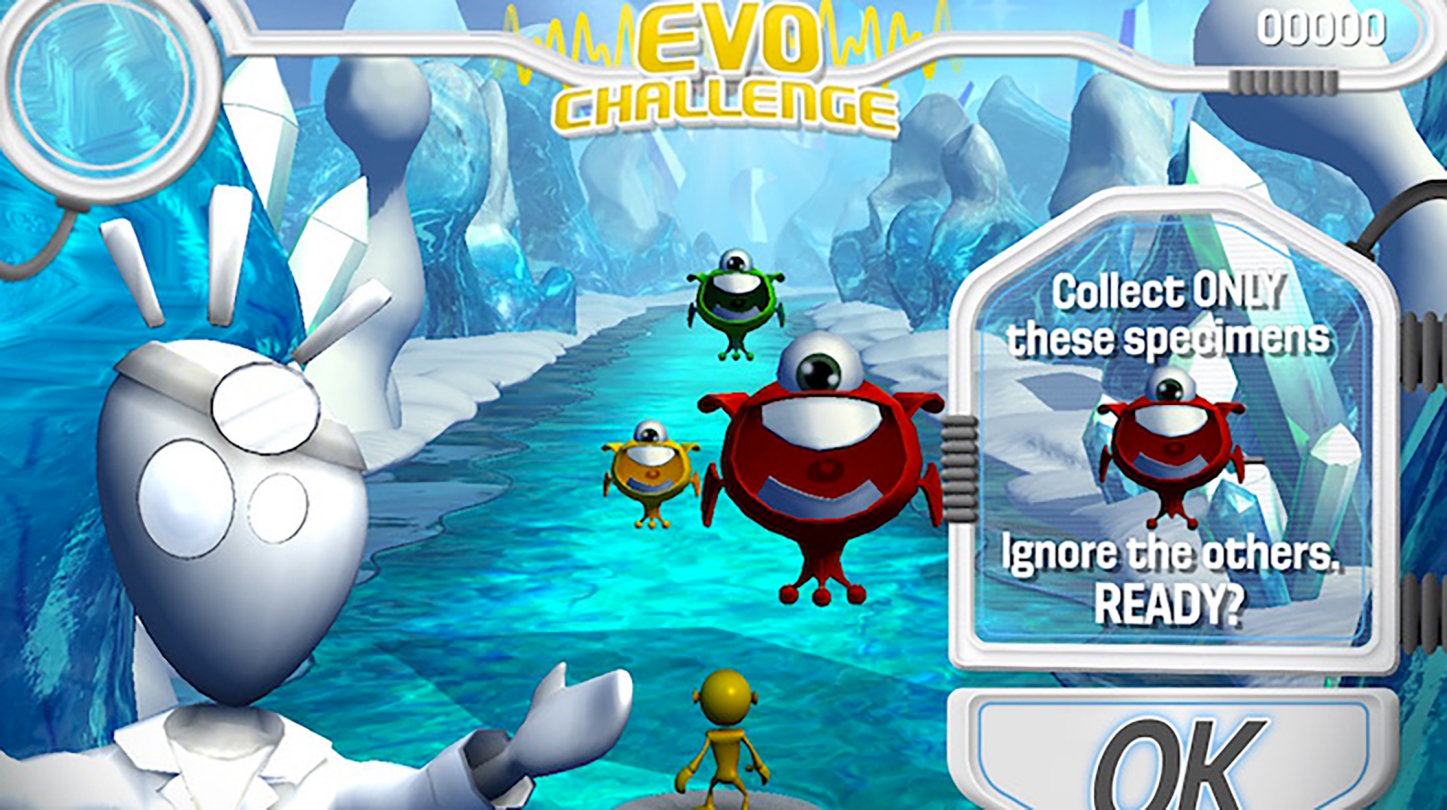
Example of a Project: Evo task.

Results of the study build upon those reported for other computerized cognitive training programs that have demonstrated positive outcomes on executive tasks that are similar to trained tasks but have shown limited generalization to other tasks [35, 36]. In the current work, findings suggest generalizability of benefits to outcome measures that are different in design and that tap domains that differ from the intervention itself. Engaging with Project: EVO’s regimen was associated with statistically significant improvements in attention and impulsivity, as measured by TOVA API and other component TOVA metrics. Both Project: EVO and TOVA contain perceptual discrimination tasks where the participant must respond to a pre-indicated target and not respond to others. However, Project: EVO differs from the TOVA in several ways: 1) EVO contains both single and multitask conditions; 2) EVO’s perceptual discrimination environment and rules change with increasingly complexity throughout treatment; and 3) EVO’s tasks are embedded in a ‘rich’ virtual environment whereas the TOVA involves a uniform task with monochromatic, geometric stimuli. See Fig 7 for a graphic of a Project: EVO targeting task. Taken together, results from TOVA metrics support modest transfer of benefit effects given the noted differences in platforms. Study results also suggested more distal transfer of benefit on the CANTAB working memory test, since the task demands on the CANTAB (e.g., spatial working memory of geometric shapes) are unlike any of the types of perceptual demands in the EVO environment.

Additionally and uniquely, *Project*: *EVO* is designed to appeal to children through a consumer-grade videogame-like format and a user-friendly at-home intervention that can be self-administered, both of which may enhance motivation and acceptability in real-world treatment deployment. Indeed, qualitative ratings provided by parents suggest that the vast majority describe the intervention as having value, and a majority of parents believed it was worthwhile enough to report the desire for access to the intervention after the study period. Importantly, despite 80% of parents indicating that the intervention was somewhat or very worthwhile, 40% of parents did not indicate a desire to continue the intervention. These parent ratings may be influenced by children’s perceptions that the intervention was challenging. As a therapeutic product, EVO attempts to balance children’s experience of both fun and challenge with effective clinical outcomes. Overall, these satisfaction findings are still considered to be very good and are consistent with prior research that parent acceptability of ADHD treatment is influenced by multiple factors [37].

Results of the current study are particularly important given that medication management, the mostly commonly utilized treatment for ADHD, has a very significant effect on core symptoms of ADHD but only equivocal effects on specific executive functions [38–40]. In addition, although the executive function profiles of individuals with ADHD are extremely heterogeneous, the presence of such deficits confers risk for more significant the impairment [41, 42]. Given the limitations of existing therapies in remediating these challenges, the current study demonstrates an important step toward increasing therapeutic options for children with ADHD and executive dysfunction. Specifically, *Project*: *EVO* was developed to address the underlying areas of neurocognitive functioning that are impacted in ADHD. In keeping with the guidelines outlined by the National Institutes of Mental Health experimental medicine initiative, treatments that directly address the underlying disease mechanism in psychiatric conditions are greatly needed [43]. Although our results are preliminary, they constitute a step in this direction.

A key part of the current proof-of-concept study was to gain an understanding of how clinically-relevant outcomes may be assessed using measures that have demonstrated utility in characterizing functioning in individuals with ADHD. For example, the BRIEF parent-report provides important clinical information about functional deficits associated with ADHD, and has been correlated with both neuroanatomy and performance on working memory measures [44, 45]. Although only a subset of BRIEF scales was examined in the current analyses, results suggest that this measure may provide a useful proxy for understanding the effects of EVO on aspects of executive functioning such as working memory and inhibition. At the same time, this parent-report measure may be less sensitive to effects of the intervention than the computer-based measures because the constructs are somewhat distinct. Similarly, deficits observed on the CANTAB among individuals with ADHD improve in the context of stimulant treatment, suggesting these are key targets for other novel interventions for this disorder [38]. The results of this study suggest that moderate improvements across a range of working memory and processing measurements on the CANTAB resulted from EVO treatment.

Several study limitations are important to note. The positive performance trajectories of children with ADHD and non-ADHD participants without ADHD were objectively demonstrated via several standardized outcome measures; however, next studies will need to include additional measures of real-world outcomes in order to determine if the cognitive improvements translate to everyday functioning. In addition, because the current study was an open-label, proof-of-concept trial, it is possible that any improvements observed via parent report were influenced by parent expectations of benefit that would be received from the intervention. However, research on cognitive training methods suggests a much smaller magnitude of improvement [46], and the TOVA test of attentional functioning has traditionally shown little to no placebo response [47], so it is likely that the current study reflects effects that are well above the impact of a placebo or expectation bias. In addition, study participants with ADHD represented a subset of the pediatric general population, but may not be representative of the entire general population of children with ADHD (e.g., not on medication, no significant co-morbid psychiatric disorders) which may affect the generalizability of these preliminary findings. Finally, given that the study’s exploratory goals involved examining efficacy effects of many outcome measures across several domains of executive function, these is concern that some results may be a result of Type I error. The current study reports Bonferroni corrected significance values to address this concern; however, future studies will need to replicate the current findings with a priori hypotheses to confirm these exploratory findings.

Although medication is the front line treatment for children with ADHD, there remains a need for the development of alternative intervention modalities to optimize care for these patients. Delivery of a digital treatment intervention on a mobile device may increase access to treatment, reduce cost of care, and increase compliance by making the treatment enjoyable and comfortable for use in an at-home setting. Future research on Project: EVO will require larger-scale randomized controlled trials to better examine the efficacy of this digital treatment for children with ADHD. These trials will benefit from including outcomes to evaluate the impact of treatment not only on executive functioning tasks and ADHD symptoms but also on the functional impairments (e.g., academic, social) that impact these children in their day-to-day lives. Additional studies that examine the amount of therapy necessary to produce a meaningful effect (i.e., the appropriate “dose” in pharmacological terms) will also be important to identify the most optimal delivery of this intervention.

## Supporting information

S1 Checklist TREND checklist. (PDF)

S1 Table TOVA raw score D prime components for the ADHD and non-ADHD groups. (PDF)

S2 Table CANTAB Spatial Working Memory. (PDF)

S3 Table Rapid Visual Processing. (PDF)

S4 Table CANTAB Delayed Match to Sample. (PDF)

S1 Dataset Study dataset. (CSV)

S1 Fig CONSORT chart. (PDF)

S1 Protocol Study protocol. (PDF)
