# Supplementary material for: Proof-of-concept study of an at-home, engaging, digital intervention for pediatric ADHD
Source: PLoS One. 2018 Jan 11;13(1):e0189749. doi: 10.1371/journal.pone.0189749 (PMC5764249; doi:10.1371/journal.pone.0189749)
Supplement: S1 Table — (PDF) [file pone.0189749.s002.pdf]

S1 Table. TOVA raw score D Prime components for the ADHD and control groups.

Outcome	Group	N	Mean-Pre (SD)	Mean-Post(SD)	Test Statistic (T/Z)	P Value	Effect Size d(r)	95% CI (U|L)
RT Mean Standard Score	ADHD	40	83.418(21.161)	87.649(22.315)	-1.3(T)	0.201	0.206	(-10.815|2.353)
	ADHD Subgroup	22	77.616(17.583)	90.773(21.128)**	-3.031(T)	0.006	0.646	(-22.185|-4.129)
	Control	40	92.709(21.001)	94.391(15.02)	-0.682(T)	0.499	0.108	(-6.668|3.305)
Variance Standard Score	ADHD	40	61.065(34.999)	72.004(28.602)	-1.613(Z)	0.109	0.266(0.255)	(-19.586|1.392)
	ADHD Subgroup	22	46.916(38.65)	73.337(26.947)*	-2.893(T)	0.009	0.617	(-45.416|-7.425)
	Control	40	81.8(21.785)	83.998(21.968)	-0.645(T)	0.523	0.102	(-9.093|4.696)
Comission Error Standard Score	ADHD	40	83.817(21.43)	88.006(22.194)	-1.203(Z)	0.234	0.163(0.19)	(-10.623|2.931)
	ADHD Subgroup	22	81.951(15.706)	86.998(19.361)	-1.251(T)	0.225	0.267	(-13.434|3.341)
	Control	40	90.198(21.553)	91.525(20.623)	-0.429(T)	0.67	0.068	(-7.575|4.923)
Omission Error Standard	ADHD	40	40.951(80.797)	-12.32(207.045)*	2.386(Z)	0.016	0.319(0.377)	(3.335|46.021)
	ADHD Subgroup	22	37.879(88.437)	8.653(128.149)	1.104(Z)	0.283	0.386(0.235)	(-7.892|52.869)

Score	Control	40	58.765(81.347)	18.496(155.73)*	2.05(Z)	0.04	0.331(0.324)	(0.323|32.334)
D Prime Standard Score	ADHD	40	76.368(7.654)	75.825(11.408)	0.364(T)	0.718	0.058	(-2.471|3.557)
	ADHD Subgroup	22	75.036(7.246)	75.396(9.566)	-0.86(Z)	0.406	0.05(0.183)	(-3.858|2.048)
	Control	40	82.42(11.498)	79.985(13.73)*	1.976(Z)	0.048	0.22(0.312)	(0.011|5.803)
ExGaussian TAU	ADHD	40	215.86(75.031)	185.301(66.832)*	2.141(T)	0.039	0.339	(1.691|59.428)
	ADHD Subgroup	22	254.034(72.418)	189.262(71.646)**	3.213(T)	0.004	0.685	(22.848|106.697)
	Control	40	169.806(58.412)	156.193(57.899)	1.505(Z)	0.135	0.241(0.238)	(-3.596|24.868)

[*] indicates statistical significance at an alpha of 0.05 (2-tailed) for pre- to post-intervention difference within group.

[**] indicates statistical significance after a Bonferroni correction of 0.05/5 = 0.01.

For each variable and group, the normality assumption for T-Tests was verified using a Shapiro-Wilks test. If the Shapiro-Wilks test indicated that the distribution of scores did not meet normality, a Wilcoxon rank sum test was performed instead. In the Test Statistic column this is indicated by a (T) or (Z) after the test statistic indicating if a T-Test (T) or a Wilcoxon test (Z) was performed. P values were calculated according to the statistical test run. Effect sizes are Cohen's d with rank-sum correlation in parentheses if appropriate.
