# Supplementary material for: Proof-of-concept study of an at-home, engaging, digital intervention for pediatric ADHD
Source: PLoS One. 2018 Jan 11;13(1):e0189749. doi: 10.1371/journal.pone.0189749 (PMC5764249; doi:10.1371/journal.pone.0189749)
Supplement: S2 Table — (PDF) [file pone.0189749.s003.pdf]

S2 Table. CANTAB Spatial Working Memory

Outcome	Group	N	Mean-Pre (SD)	Mean-Post(SD)	Test Statistic (T/Z)	P Value	Effect Size d(r)	95% CI (U|L)
Between Errors	ADHD	40	18.975(7.921)	17.275(8.527)	1.255(T)	0.217	0.198	(-1.041|4.441)
	ADHD Subgroup	22	19.591(7.805)	18.227(9.263)	0.678(T)	0.505	0.145	(-2.819|5.547)
	Control	40	17.575(9.397)	16.3(9.433)	0.957(T)	0.344	0.151	(-1.419|3.969)
Double Errors	ADHD	40	0.35(0.622)	0.5(0.934)	-0.429(Z)	0.672	0.146(0.068)	(-1|1)
	ADHD Subgroup	22	0.364(0.658)	0.364(0.727)	0.277(Z)	0.984	0(0.059)	(-1.5|1.5)
	Control	40	0.425(0.984)	0.35(0.77)	0.264(Z)	0.809	0.063(0.042)	(-1|1)
Mean time to First Response 4 Boxes	ADHD	40	1894.525(1307.187)	1213.4(314.669)**	4.194(Z)	<0.001	0.574(0.663)	(248.75|678.75)
	ADHD Subgroup	22	2147.5(1521.651)	1291.455(330.797)**	3.587(Z)	<0.001	0.642(0.765)	(288.25|1016.75)
	Control	40	1927.338(1858.711)	1284.175(501.945)**	3.602(Z)	<0.001	0.353(0.57)	(143|514.75)
Mean Time to Last	ADHD	40	15333.15(3287.435)	12084.275(1542.645)**	5.269(Z)	<0.001	1.092(0.833)	(2157.5|3664.5)
	ADHD	22	16175.636(3733.025)	12487.773(1776.035)**	3.912(Z)	<0.001	1.049(0.834)	(2003.5|4905.25)

<table>
<tr><td>Response 4 Boxes</td><td>Subgroup</td><td></td><td></td><td></td><td></td><td></td><td></td><td></td></tr>
<tr><td></td><td>Control</td><td>40</td><td>15035.488(3499.211)</td><td>13052.05(2203.998)**</td><td>4.18(Z)</td><td><0.001</td><td>0.67(0.661)</td><td>(873.75|2527.5)</td></tr>
<tr><td>Mean Token Search Preparation Time 4 Boxes</td><td>ADHD</td><td>40</td><td>1245.756(473.744)</td><td>817.381(188.443)**</td><td>5.256(Z)</td><td><0.001</td><td>1.063(0.831)</td><td>(267.438|510.688)</td></tr>
<tr><td></td><td>ADHD Subgroup</td><td>22</td><td>1337.335(525.314)</td><td>861.522(202.678)**</td><td>3.945(Z)</td><td><0.001</td><td>1.11(0.841)</td><td>(262.253|637.568)</td></tr>
<tr><td></td><td>Control</td><td>40</td><td>1227.375(632.854)</td><td>942.575(260.657)**</td><td>3.676(Z)</td><td><0.001</td><td>0.545(0.581)</td><td>(98.688|344.69)</td></tr>
<tr><td>SD Time to First Response</td><td>ADHD</td><td>40</td><td>758.584(1021.912)</td><td>298.611(307.757)**</td><td>3.844(Z)</td><td><0.001</td><td>0.531(0.608)</td><td>(158.391|518.308)</td></tr>
<tr><td></td><td>ADHD Subgroup</td><td>22</td><td>956.522(1301.225)</td><td>321.027(357.91)**</td><td>3.458(Z)</td><td><0.001</td><td>0.602(0.737)</td><td>(179.959|691.903)</td></tr>
<tr><td></td><td>Control</td><td>40</td><td>912.398(2447.527)</td><td>286.873(360.143)*</td><td>2.07(Z)</td><td>0.038</td><td>0.251(0.327)</td><td>(4.95|299.46)</td></tr>
<tr><td>SD Time to Last Response 4 Boxes</td><td>ADHD</td><td>40</td><td>1708.9(1849.87)</td><td>839.371(921.141)**</td><td>3.589(Z)</td><td><0.001</td><td>0.528(0.567)</td><td>(297.341|937.624)</td></tr>
<tr><td></td><td>ADHD Subgroup</td><td>22</td><td>1930.209(2100.86)</td><td>1048.382(1142.766)*</td><td>2.386(Z)</td><td>0.016</td><td>0.488(0.509)</td><td>(103.592|1129.253)</td></tr>
<tr><td></td><td>Control</td><td>40</td><td>1858.859(2978.088)</td><td>1260.877(1301.018)</td><td>0.753(Z)</td><td>0.46</td><td>0.184(0.119)</td><td>(-323.856|748.471)</td></tr>
<tr><td>SD Token Search Preparation</td><td>ADHD</td><td>40</td><td>721.079(664.592)</td><td>368.823(156.479)**</td><td>4.194(Z)</td><td><0.001</td><td>0.588(0.663)</td><td>(118.996|382.036)</td></tr>
<tr><td></td><td>ADHD Subgroup</td><td>22</td><td>871.935(793.08)</td><td>411.937(155.504)**</td><td>3.587(Z)</td><td><0.001</td><td>0.657(0.765)</td><td>(135.913|578.045)</td></tr>
</table>

Time 4 Boxes	Control	40	769.768(1206.306)	415.354(249.53)**	3.011(Z)	0.002	0.302(0.476)	(50.74|223.006)
Stratagy	ADHD	40	17.925(2.495)	17(2.522)*	2.578(T)	0.014	0.408	(0.199|1.651)
	ADHD Subgroup	22	17.955(2.984)	16.682(2.514)*	2.261(T)	0.034	0.482	(0.102|2.443)
	Control	40	17.35(2.94)	17.15(2.975)	0.403(T)	0.689	0.064	(-0.804|1.204)
Stratagy 4 Boxes	ADHD	40	22.95(2.873)	21.575(2.845)**	3.193(T)	0.003	0.505	(0.504|2.246)
	ADHD Subgroup	22	23.045(3.387)	21.455(3.004)*	2.526(T)	0.02	0.538	(0.281|2.901)
	Control	40	22.65(3.655)	21.8(3.51)	1.407(T)	0.167	0.223	(-0.372|2.072)
Total Errors	ADHD	40	19.15(7.95)	17.575(8.741)	1.156(T)	0.255	0.183	(-1.181|4.331)
	ADHD Subgroup	22	19.864(7.797)	18.364(9.373)	0.738(T)	0.469	0.157	(-2.726|5.726)
	Control	40	17.95(9.648)	16.475(9.568)	1.055(T)	0.298	0.167	(-1.352|4.302)
Within Errors	ADHD	40	0.525(0.905)	0.8(1.4)	-0.831(Z)	0.417	0.177(0.131)	(-1.5|0.5)
	ADHD Subgroup	22	0.636(1.093)	0.5(0.859)	0.554(Z)	0.637	0.106(0.118)	(-1.5|2)
	Control	40	0.8(2.015)	0.525(1.24)	0.761(Z)	0.472	0.117(0.12)	(-1.5|2.5)

* indicates statistical significance at an alpha of 0.05 (2-tailed) for pre- to post-intervention difference within group.

** indicates statistical significance after a Bonferroni correction of 0.05/12 = 0.004.

For each variable and group, the normality assumption for T-Tests was verified using a Shapiro-Wilks test. If the Shapiro-Wilks test indicated that the distribution of scores did not meet normality, a Wilcoxon rank sum test was performed instead. In the Test Statistic column this is indicated by a (T) or (Z) after the test statistic indicating if a T-Test (T) or a Wilcoxon test (Z) was performed. P values were calculated according to the statistical test run. Effect sizes are Cohen's d with rank-sum correlation in parentheses if appropriate.
