# Supplementary material for: Proof-of-concept study of an at-home, engaging, digital intervention for pediatric ADHD
Source: PLoS One. 2018 Jan 11;13(1):e0189749. doi: 10.1371/journal.pone.0189749 (PMC5764249; doi:10.1371/journal.pone.0189749)
Supplement: S3 Table — (PDF) [file pone.0189749.s004.pdf]

S3 Table. Rapid Visual Processing

Outcome	Group	N	Mean-Pre (SD)	Mean-Post(SD)	Test Statistic (T/Z)	P Value	Effect Size d(r)	95% CI (U|L)
A Prime	ADHD	40	0.789(0.05)	0.805(0.055)*	-2.119(T)	0.04	0.335	(-0.031|-0.001)
	ADHD Subgroup	22	0.798(0.044)	0.792(0.058)	0.655(T)	0.52	0.14	(-0.014|0.026)
	Control	40	0.808(0.046)	0.834(0.058)**	-3.848(T)	<0.001	0.608	(-0.039|-0.012)
B Prime	ADHD	40	0.706(0.27)	0.732(0.224)	-0.874(Z)	0.39	0.116(0.138)	(-0.071|0.039)
	ADHD Subgroup	22	0.742(0.249)	0.689(0.236)	1.306(T)	0.206	0.279	(-0.032|0.139)
	Control	40	0.853(0.158)	0.827(0.186)	1.169(Z)	0.248	0.15(0.185)	(-0.012|0.057)
Mean Latency	ADHD	40	671.89(176.507)	607.429(165.438)*	2.678(T)	0.011	0.423	(15.765|113.156)
	ADHD Subgroup	22	668.972(203.718)	617.704(179.596)	1.394(T)	0.178	0.297	(-25.21|127.745)
	Control	40	616.072(163.363)	549.698(140.712)**	3.898(Z)	<0.001	0.498(0.616)	(34.998|95.933)
Probability of	ADHD	40	0.055(0.082)	0.042(0.048)	0.538(Z)	0.599	0.179(0.085)	(-0.007|0.014)

False Alarm	ADHD Subgroup	22	0.043(0.057)	0.049(0.048)	-0.622(T)	0.541	0.133	(-0.026|0.014)
	Control	40	0.018(0.026)	0.023(0.032)	-1.075(Z)	0.289	0.189(0.17)	(-0.006|0.001)
Probability of Hit	ADHD	40	0.334(0.135)	0.371(0.124)	-1.624(T)	0.112	0.257	(-0.083|0.009)
	ADHD Subgroup	22	0.339(0.118)	0.355(0.114)	-0.492(T)	0.628	0.105	(-0.084|0.052)
	Control	40	0.31(0.139)	0.409(0.2)**	-4.369(T)	<0.001	0.691	(-0.146|-0.053)
SD Latency	ADHD	40	316.615(122.399)	308.776(105.853)	0.36(T)	0.721	0.057	(-36.217|51.895)
	ADHD Subgroup	22	320.04(115.998)	314.067(116.257)	0.237(T)	0.815	0.05	(-46.514|58.461)
	Control	40	269.9(115.645)	266.67(115.782)	0.192(T)	0.849	0.03	(-30.867|37.327)
Total Correct Rejections	ADHD	40	440.575(36.409)	451.85(27.58)*	-2.481(Z)	0.012	0.361(0.392)	(-15|-2)
	ADHD Subgroup	22	446.5(26.133)	447.227(28.574)	-0.166(T)	0.87	0.035	(-9.847|8.392)
	Control	40	458(18.383)	466.825(26.952)**	-3.152(T)	0.003	0.498	(-14.487|-3.163)
Total False Alarms	ADHD	40	25.925(39.316)	19.775(22.758)	0.504(Z)	0.62	0.182(0.08)	(-3.5|7)
	ADHD Subgroup	22	20.227(27.041)	22.818(22.187)	-0.576(T)	0.571	0.123	(-11.949|6.767)

<table>
<tr><td></td><td>Control</td><td>40</td><td>8.325(11.93)</td><td>10.725(15.274)</td><td>-1.315(Z)</td><td>0.192</td><td>0.204(0.208)</td><td>(-4.5|1)</td></tr>
<tr><td rowspan="3">Total Hits</td><td>ADHD</td><td>40</td><td>18(7.278)</td><td>20(6.653)</td><td>-1.627(T)</td><td>0.112</td><td>0.257</td><td>(-4.486|0.486)</td></tr>
<tr><td>ADHD Subgroup</td><td>22</td><td>18.273(6.318)</td><td>19.136(6.105)</td><td>-0.493(T)</td><td>0.627</td><td>0.105</td><td>(-4.505|2.778)</td></tr>
<tr><td>Control</td><td>40</td><td>16.725(7.511)</td><td>22.1(10.775)**</td><td>-4.369(T)</td><td><0.001</td><td>0.691</td><td>(-7.864|-2.886)</td></tr>
<tr><td rowspan="3">Total Misses</td><td>ADHD</td><td>40</td><td>35.975(7.308)</td><td>33.975(6.685)</td><td>1.619(T)</td><td>0.114</td><td>0.256</td><td>(-0.499|4.499)</td></tr>
<tr><td>ADHD Subgroup</td><td>22</td><td>35.682(6.38)</td><td>34.818(6.177)</td><td>0.489(T)</td><td>0.63</td><td>0.104</td><td>(-2.811|4.538)</td></tr>
<tr><td>Control</td><td>40</td><td>37.275(7.511)</td><td>31.9(10.775)**</td><td>4.369(T)</td><td><0.001</td><td>0.691</td><td>(2.886|7.864)</td></tr>
</table>

* indicates statistical significance at an alpha of 0.05 (2-tailed) for difference from pre- to post-intervention within group.

** indicates statistical significance after a Bonferroni correction of 0.05/10 = 0.005.

For each variable and group, the normality assumption for T-Tests was verified using a Shapiro-Wilks test. If the Shapiro-Wilks test indicated that the distribution of scores did not meet normality, a Wilcoxon rank sum test was performed instead. In the Test Statistic column this is indicated by a (T) or (Z) after the test statistic indicating if a T-Test (T) or a Wilcoxon test (Z) was performed. P values were calculated according to the statistical test run. Effect sizes are Cohen's d with rank-sum correlation in parentheses if appropriate.
