# Supplementary material for: Proof-of-concept study of an at-home, engaging, digital intervention for pediatric ADHD
Source: PLoS One. 2018 Jan 11;13(1):e0189749. doi: 10.1371/journal.pone.0189749 (PMC5764249; doi:10.1371/journal.pone.0189749)
Supplement: S4 Table — (PDF) [file pone.0189749.s005.pdf]

S4 Table. CANTAB Delayed Match to Sample

Outcome	Group	N	Mean-Pre (SD)	Mean-Post(SD)	Test Statistic (T/Z)	P Value	Effect Size d(r)	95% CI (U|L)
A Prime	ADHD	40	0.289(0.335)	0.229(0.285)	0.918(T)	0.364	0.145	(-0.072|0.191)
	ADHD Subgroup	22	0.28(0.316)	0.22(0.286)	0.647(T)	0.525	0.138	(-0.134|0.255)
	Control	40	0.312(0.318)	0.104(0.362)*	2.491(Z)	0.012	0.403(0.394)	(0.042|0.63)
D Prime	ADHD	40	-0.493(0.553)	-0.547(0.473)	0.497(T)	0.622	0.079	(-0.164|0.271)
	ADHD Subgroup	22	-0.498(0.55)	-0.512(0.473)	0.085(T)	0.933	0.018	(-0.322|0.349)
	Control	40	-0.333(0.518)	-0.598(0.533)*	2.181(T)	0.035	0.345	(0.019|0.511)
Errors Correct Color	ADHD	40	2(1.601)	1.775(1.368)	1.215(Z)	0.233	0.122(0.192)	(-0.5|1.5)
	ADHD Subgroup	22	1.909(1.63)	1.591(1.469)	0.688(T)	0.499	0.147	(-0.643|1.28)
	Control	40	2.25(1.676)	1.675(1.76)*	2.345(T)	0.024	0.371	(0.079|1.071)
Errors Correct	ADHD	40	1.75(1.515)	1.525(1.154)	0.844(T)	0.404	0.133	(-0.314|0.764)
	ADHD	22	2.091(1.601)	1.727(1.162)	0.954(T)	0.351	0.203	(-0.429|1.156)

<table>
<tr><td>Shape</td><td>Subgroup</td><td></td><td></td><td></td><td></td><td></td><td></td><td></td></tr>
<tr><td></td><td>Control</td><td>40</td><td>1.525(1.358)</td><td>1.2(1.018)</td><td>1.407(Z)</td><td>0.169</td><td>0.245(0.223)</td><td>(0|1.5)</td></tr>
<tr><td rowspan="3">Errors Novel Distractor</td><td>ADHD</td><td>40</td><td>0.425(0.636)</td><td>0.725(1.012)</td><td>-1.587(Z)</td><td>0.114</td><td>0.254(0.251)</td><td>(-1|0)</td></tr>
<tr><td>ADHD Subgroup</td><td>22</td><td>0.545(0.671)</td><td>0.682(0.945)</td><td>-0.568(T)</td><td>0.576</td><td>0.121</td><td>(-0.635|0.363)</td></tr>
<tr><td>Control</td><td>40</td><td>0.25(0.494)</td><td>0.325(0.764)</td><td>-0.044(Z)</td><td>1</td><td>0.094(0.007)</td><td>(-1.5|1)</td></tr>
<tr><td rowspan="3">Mean Choices to Correct</td><td>ADHD</td><td>40</td><td>1.309(0.208)</td><td>1.311(0.219)</td><td>-0.063(T)</td><td>0.95</td><td>0.01</td><td>(-0.09|0.084)</td></tr>
<tr><td>ADHD Subgroup</td><td>22</td><td>1.361(0.226)</td><td>1.316(0.218)</td><td>0.702(T)</td><td>0.49</td><td>0.15</td><td>(-0.089|0.179)</td></tr>
<tr><td>Control</td><td>40</td><td>1.285(0.235)</td><td>1.214(0.192)**</td><td>2.941(Z)</td><td>0.003</td><td>0.474(0.465)</td><td>(0.025|0.15)</td></tr>
<tr><td rowspan="3">Mean Correct Latency</td><td>ADHD</td><td>40</td><td>3838.253(1004.022)</td><td>4033.465(1411.669)</td><td>-0.659(Z)</td><td>0.519</td><td>0.176(0.104)</td><td>(-534.77|260.485)</td></tr>
<tr><td>ADHD Subgroup</td><td>22</td><td>3967.934(1056.867)</td><td>4519.585(1463.618)*</td><td>-2.101(T)</td><td>0.048</td><td>0.448</td><td>(-1097.698|-5.604)</td></tr>
<tr><td>Control</td><td>40</td><td>3832.673(1224.693)</td><td>3735.877(1032.766)</td><td>0.571(T)</td><td>0.571</td><td>0.09</td><td>(-245.842|439.435)</td></tr>
<tr><td>Mean Correct</td><td>ADHD</td><td>40</td><td>3975.09(1172.726)</td><td>4069.029(1567.16)</td><td>-0.525(T)</td><td>0.602</td><td>0.083</td><td>(-455.728|267.85)</td></tr>
</table>

Latency All Delays	ADHD Subgroup	22	4045.57(1173.69)	4527.342(1651.793)	-1.87(T)	0.075	0.399	(-1017.506|53.96)
	Control	40	3948.5(1351.799)	3868.191(1168.202)	0.439(T)	0.663	0.069	(-289.589|450.208)
Percent Correct	ADHD	40	79.125(11.706)	79.875(11.463)	-0.327(T)	0.746	0.052	(-5.395|3.895)
	ADHD Subgroup	22	77.273(12.605)	80(11.952)	-0.777(T)	0.446	0.166	(-10.023|4.569)
	Control	40	79.875(12.785)	84(11.613)*	-2.611(T)	0.013	0.413	(-7.321|-0.929)
Percent Correct All Delays	ADHD	40	73.833(15.351)	74.833(14.04)	-0.348(T)	0.729	0.055	(-6.805|4.805)
	ADHD Subgroup	22	71.515(16.353)	74.545(14.926)	-0.685(T)	0.501	0.146	(-12.231|6.17)
	Control	40	75.5(14.822)	80.333(13.499)*	-2.293(T)	0.027	0.363	(-9.097|-0.569)
Probability of Error Given Correct	ADHD	40	0.219(0.135)	0.219(0.122)	-0.037(T)	0.971	0.006	(-0.052|0.05)
	ADHD Subgroup	22	0.231(0.139)	0.216(0.127)	0.416(T)	0.681	0.089	(-0.06|0.09)
	Control	40	0.205(0.143)	0.189(0.175)	1.19(Z)	0.238	0.107(0.188)	(-0.022|0.068)
Probability of Error	ADHD	40	0.2(0.24)	0.152(0.19)	0.548(Z)	0.59	0.163(0.087)	(-0.069|0.214)
	ADHD	22	0.18(0.199)	0.149(0.19)	0.496(T)	0.625	0.106	(-0.098|0.159)

Given Error	Subgroup							
	Control	40	0.191(0.183)	0.091(0.143)*	2.633(Z)	0.007	0.454(0.416)	(0.066|0.306)
SD Correct Latency	ADHD	40	1873.921(921.102)	2139.495(1544.993)	-0.013(Z)	0.995	0.173(0.002)	(-370.6|256.368)
	ADHD Subgroup	22	1975.796(1104.631)	2635.081(1869.989)	-1.153(Z)	0.262	0.344(0.246)	(-1061.905|203.69)
	Control	40	1793.727(866.628)	1752.398(767.867)	0.317(T)	0.753	0.05	(-222.786|305.444)
SD Correct Latency All Delays	ADHD	40	1932.028(950.722)	2093.342(1347.096)	-0.04(Z)	0.973	0.141(0.006)	(-421.11|308.08)
	ADHD Subgroup	22	2010.047(1120.301)	2504.436(1589.51)	-1.38(Z)	0.176	0.362(0.294)	(-914.67|256.37)
	Control	40	1916.356(978.547)	1789.329(825.578)	0.874(T)	0.388	0.138	(-167.046|421.1)
Total Correct	ADHD	40	15.825(2.341)	15.975(2.293)	-0.327(T)	0.746	0.052	(-1.079|0.779)
	ADHD Subgroup	22	15.455(2.521)	16(2.39)	-0.777(T)	0.446	0.166	(-2.005|0.914)
	Control	40	15.975(2.557)	16.8(2.323)*	-2.611(T)	0.013	0.413	(-1.464|-0.186)
Total Correct All Delays	ADHD	40	11.075(2.303)	11.225(2.106)	-0.348(T)	0.729	0.055	(-1.021|0.721)
	ADHD Subgroup	22	10.727(2.453)	11.182(2.239)	-0.685(T)	0.501	0.146	(-1.835|0.926)

	Control	40	11.325(2.223)	12.05(2.025)*	-2.293(T)	0.027	0.363	(-1.365|-0.085)

* indicates statistical significance at an alpha of 0.05 (2-tailed) for difference from pre- to post-intervention within group.

** indicates statistical significance after a Bonferroni correction of 0.05/16 = 0.003.

The above table shows the results for the CANTAB DMS test. For each outcome the results are presented for the ADHD, ADHD Subgroup, and the Control groups. For each variable and group, the normality assumption for T-Tests was verified using a Shapiro-Wilks test. If the Shapiro-Wilks test indicated that the distribution of scores did not meet normality, a Wilcoxon rank sum test was performed instead. In the Test Statistic column this is indicated by a (T) or (Z) after the test statistic indicating if a T-Test (T) or a Wilcoxon test (Z) was performed. P values were calculated according to the statistical test run. Effect sizes are Cohen's d with rank-sum correlation in parentheses if appropriate.
