# Supplementary figures and images for: Proof-of-concept study of an at-home, engaging, digital intervention for pediatric ADHD
Source: PLoS One. 2018 Jan 11;13(1):e0189749. doi: 10.1371/journal.pone.0189749 (PMC5764249; doi:10.1371/journal.pone.0189749)

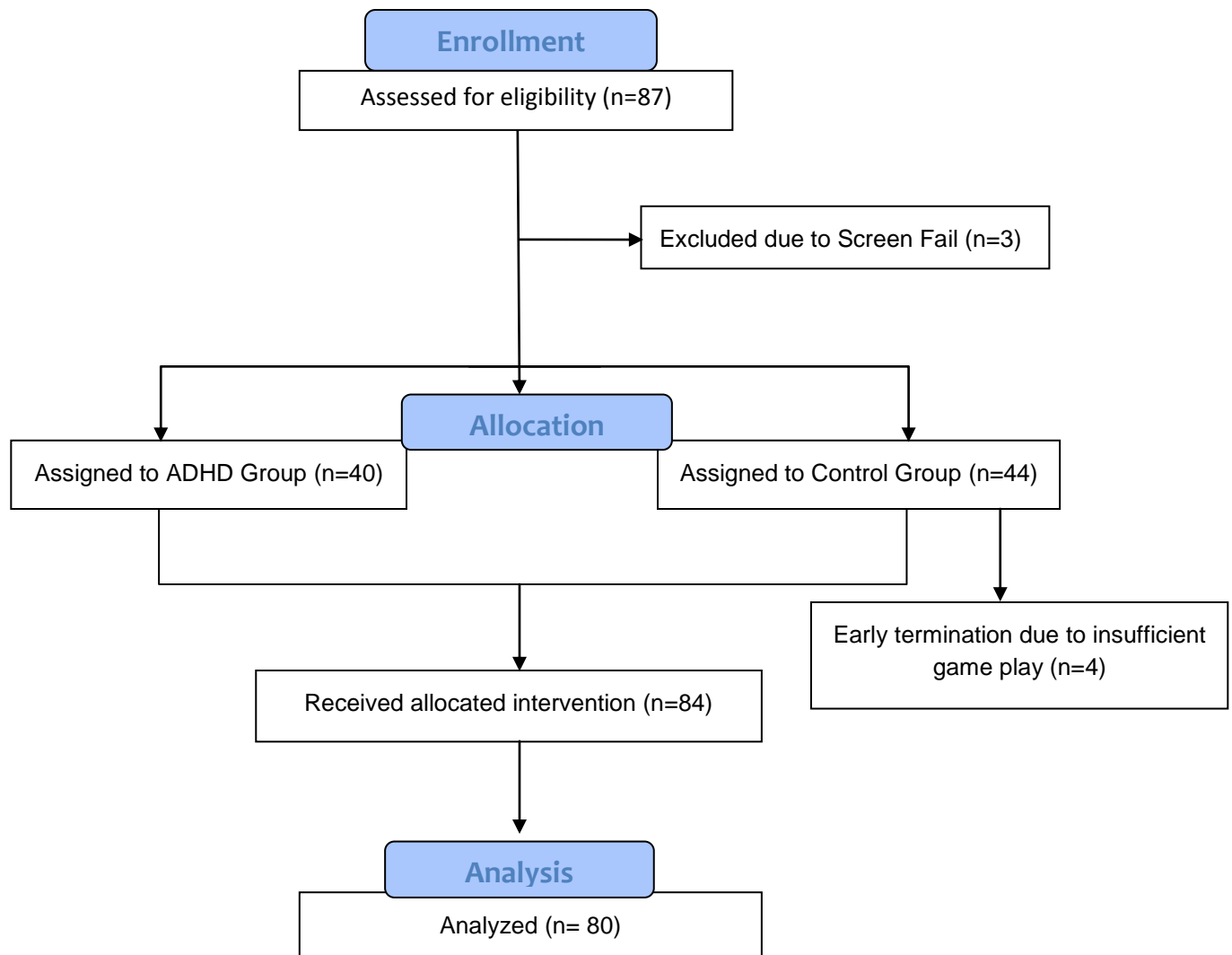

Supplement: S1 Fig — (PDF) [file pone.0189749.s007.pdf]
