# Supplementary material for: Proof-of-concept study of an at-home, engaging, digital intervention for pediatric ADHD
Source: PLoS One. 2018 Jan 11;13(1):e0189749. doi: 10.1371/journal.pone.0189749 (PMC5764249; doi:10.1371/journal.pone.0189749)
Supplement: S1 Protocol — (PDF) [file pone.0189749.s008.pdf]

Long Protocol Title

A study to assess the feasibility of EVO game-play to engage participants with Attention Deficit Hyperactivity Disorder (ADHD) and to evaluate interference cost in children ages 8 to 12 years old with ADHD compared to age-matched controls.

Short Protocol Title

Cognitive ADHD Video Game Exploratory Study (CAVES)

Sponsor

Akili Interactive Labs, Inc., 500 Boylston Street, Suite 1600, Boston, MA 02116
Contact: Scott Kellogg, Vice President, 617-456-0984

Principal Investigator

Andrew J. Cutler, MD
Florida Clinical Research Center, LLC
8043 Cooper Creek Blvd, Suite 107
Bradenton, FL 32401
DAYTIME TELEPHONE NUMBER(S): 941-747-7900
24-HOUR CONTACT NUMBER(S): 941-747-7900

Co-Investigator

Joanne L. Northcutt, PhD
Florida Clinical Research Center, LLC
2300 Maitland Center Parkway, Suite 230
Maitland FL 32751
DAYTIME TELEPHONE NUMBER(S): 407-644-1165
24-HOUR CONTACT NUMBER(S): 407-644-1165

Co-Investigator

Scott H. Kollins, Ph.D.
Professor & Vice-Chair for Research Strategy & Development
Director, Duke ADHD Program
Department of Psychiatry & Behavioral Sciences
Duke University
Durham, NC
DAYTIME TELEPHONE NUMBER(S): (919) 681-0014
24-HOUR CONTACT NUMBER(S): (919) 681-0014

Confidential

The recipient of this document agrees to keep it strictly confidential. The information contained in this document must not be communicated to a third party without prior written approval of Akili.

Signature Page

Sponsor
Scott Kellogg, Vice President, Akili Interactive Labs, Inc.

Signature/Date:___________________________

Principal Investigator
Andrew J. Cutler, MD, Florida Clinical Research Center, LLC

Signature/Date:___________________________

Co-Investigator
Joanne L. Northcutt, PhD, Florida Clinical Research Center, LLC

Signature/Date:__________________________

Co-Investigator
Scott H. Kollins, Ph.D., Professor & Vice-Chair for Research Strategy & Development, Duke University

Signature/Date:__________________________

Table of Contents

Section 1: Information for the Investigator **5**
1.1: Historic Background 5
1.2: Scientific Background 5
1.3: Rationale 7
Section 2: Objectives of the Study **7**
2.1: Study Overview 7
2.2: Primary Objective 7
2.3: Secondary Objective 8
2.4: Exploratory Objective 9
Section 3: Participant Selection **9**
3.1: Inclusion Criteria 9
3.2: Exclusion Criteria 10
3.3: Concomitant Medication and Treatment Criteria 10
3.4: Participant Selection 11
Section 4: Participant Enrollment **11**
4.1: Methods of enrollment 11
4.2: Informed Consent / Child Assent Form (ICF/CAF) 12
4.3: Treatment Assignment 12
4.4: Participant Withdrawal 12
Section 5: Study Procedures **13**
5.1: Study visits and Procedures 13
5.1.1: Day 0 In-clinic Screening & Baseline 14
5.1.2: Day 1 to Day 27 at home EVO play 17
5.1.3: Day 28 In-clinic Baseline and Exit 17
5.2: Study Product 19
5.3: Participant reimbursement 19
Section 6: Data Management and Analysis **19**
6.1: Data Collection and Transfer 19
6.2: Statistical Analysis 21
Section 7: Risk / Benefit **21**
Section 8: Monitoring and Quality Assurance **22**
8.1: Monitoring of Source Data 22
8.2: Safety Monitoring 22
8.3: Outcomes Monitoring 22
8.4: Protection of Participants 23
8.5: Adverse Events (AEs) 23

Appendices
Appendix A: Study Synopsis 26
Appendix B: Example Informed Consent 32
Appendix C: Example Child Assent 40
Appendix D: Participant Questionnaire 44
Appendix E: Akili Executive Summary 46

Appendix F: Parent BRIEF …………………………………………………………………….. 47
Appendix G: CANTAB (ADHD) ……………………………………………………………. 50
Appendix H: ADHD-RS ……………………………………………………………………….. 51
Appendix I: MINI-Kid ………………………………………………………………………….. 52
Appendix J: EVO Game Play Instructions for the Parent and Participant.. 55
Appendix K: EVO Game Play Instructions for the Investigator ……............. 57
Appendix L: Columbia-Suicide Severity Rating Scale (C-SSRS) ……………... 59
Appendix M: Study Staff Observation of Game-play ……………………………. 65
Appendix N: Parent or Guardian Questionnaire ……….……………………… 66

Section 1: Information for the Investigator

1.1: Historic Background

Therapeutic gaming has recently been identified as a promising approach to cognitive therapy (Bavelier and Davidson, Nature (2013)). Recently a mobile video game called "EVO" (developed by Akili Interactive Labs. Inc., Boston, MA) has been designed to measure and improve the ability to process cognitive interference (essentially, interruptions and distractions), a limiting factor across global executive function that is known to be fragile in multiple conditions including ADHD and autism. The basic scientific mechanisms of EVO cognitive game play have been validated by Dr. Adam Gazzaley, M.D. (UCSF) in a controlled, N=203 study of healthy subjects to explore the ability of the game mechanics to precisely measure cognitive interference, and to examine the ability to enhance interference processing. Training on the game mechanics was shown to enhance the ability of aging adults, who are a model population of interference susceptibility, to resolve interference and enhance their general cognitive function including sustained attention, impulsivity, and working memory. Importantly, follow-ups 6 months after the study showed maintenance of benefits in the training group that correlated with statistically-significant brain changes as measured by EEG.
While the Gazzaley study demonstrated improvement in healthy populations along specific cognitive axes that are considered to be weakened in ADHD and ADS disability populations, this proposed study will aim to directly explore the feasibility of this approach in children diagnosed with ADHD. This study will be an early step in the development of an empirically-validated novel therapeutic tool for adolescent and adult cognitive conditions.

For additional information on Akili Interactive Labs, Inc. see Appendix E (Akili Executive Summary).

1.2: Scientific Background

<u>Overview</u>
EVO is the first gaming engine that is entirely designed based on validated neuroscience. The game is focused directly on assessing and enhancing executive function abilities through the targeting of the player's ability to process cognitive interference, a fragile pathway that underlies multiple top-level cognitive domains, including working memory, attention, and motor abilities (Gazzaley et al., *Nature Neuroscience (2005), Gazzaley et al., PNAS (2008), Chadick & Gazzaley, Nature Neuroscience (2011), Clapp et al., PNAS (2011), Wais et al., Cereb Cortex (2012)*).
EVO is designed as part of an adaptive gaming engine; it adapts real-time and between sessions for a unique training experience, automatically tailored to each player, and includes real-time feedback necessary for rapid learning. This pushes the player precisely at pre-defined cognitive bounds relative to the player – a feature critical for cognitive enhancement.

<u>The Science Behind EVO</u>
EVO was designed based on the seminal work of two leading cognitive neuroscientists: Dr. Adam Gazzaley M.D., Founding Director of the Neuroscience Imaging Center at UCSF and leader in understanding the neural mechanisms of interference processing; and Dr. Daphne Bavelier, Professor of Brain and Cognitive Sciences at University of Rochester and arguably the world's leading authority on the beneficial cognitive effects of action video games. Both Dr. Gazzaley and Dr. Bavelier have

published their studies on human participants in top-tier biomedical journals (Green and Bavelier, *Nature (2003),* Gazzaley et al., *Nature Neuroscience (2005),* Gazzaley et al*., PNAS (2008),* Bavelier et al., *Nat Rev Neursci (2011)*).

EVO has incorporated a novel, soon-to-be-published interference training program developed by Dr. Gazzaley, which has been validated in a training study at UCSF, into a mobile adaptive platform game. The proof of concept study showed the ability to enhance healthy individuals' interference processing, a cognitive benefit that also transferred to improved performance on untrained, gold-standard cognitive tests of attention, impulsivity, and multitasking. (Anguera et al, *in revision at Nature*)

The first prototype of EVO was tested on 203 individuals from 8-80 years old, showing the ability of the video game to precisely measure susceptibility to interference (Figure 1). Significantly, the mechanics can detect "elite" and "severely underperforming" individuals within a generally well-performing healthy group of young healthy adults (Figure 1 – *Inset*). More importantly, the effects of cognitive enhancement with these mechanics were validated in the group with the largest deficit, aging adults (who are a model population of interference susceptibility), and showed the ability to improve interference processing to that of a healthy 20 year-old. The cognitive benefits of training appear to transfer to untrained tasks beyond the game environment, as the participants who received the proprietary training technology showed statistically-significant improvements on gold-standard tests of multitasking (PRP dual task), sustained attention and impulsivity (Tests of variables of attention, or TOVA), and working memory (facial recognition task) compared with controls (Figure 2). Finally, follow-ups after 6 months of no training showed maintenance of benefits in the training group, and these benefits correlated with statistically significant brain changes as measured by EEG, demonstrating a true physiological effect of training on the human brain.

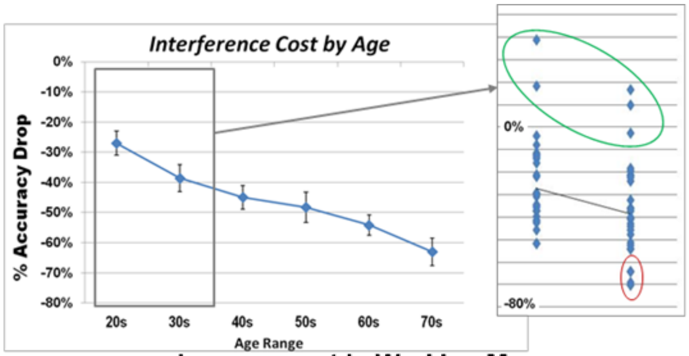

Figure 1: Interference Susceptibility by Age as determined by Akili paradigm
Inset: distribution highlighting elite (green) and low-tier (red) individual performers from 20s and 30s

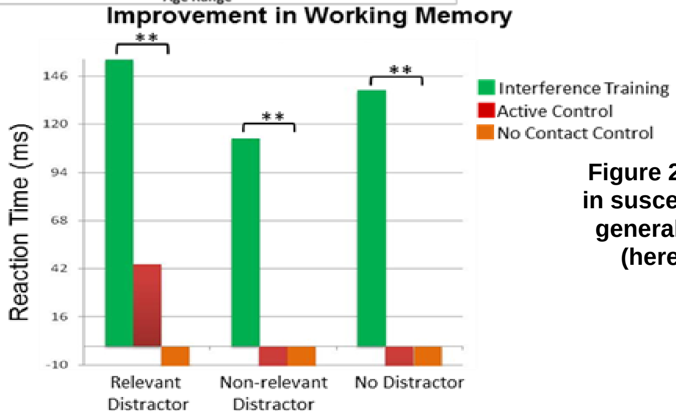

Figure 2: Interference Training benefits in susceptible individuals (aging adults) generalize to core cognitive functions (here: Delayed-Recognition Task)

Significance

While the Gazzaley study was tested in neuro-typical volunteers, interference susceptibility ("distractibility") leading to decreased real-world performance is an accepted deficit in individuals with an ADHD and autism classification. Also, the cognitive domains that showed marked improvement after training in the academic study are those that most closely related to these neuro-atypical populations (sustained attention, impulsivity, and working memory).

The proposed study will be one of a collection of respected academic studies that will make the first strides towards bringing an exciting, non-invasive video game paradigm into specific neuro-atypical populations in a clinically-rigorous validation pathway.

1.3: Rationale

Currently available treatment options for ADHD are limited, thus there is a great demand for new treatment options. This study will build on previous work establishing that cognitive deficits can be improved by therapeutic gaming. Therapeutic gaming could ultimately be prescribed as adjunctive therapy or stand-alone therapy to traditional pharmacologic practice. Gaming has the potential to provide benefit without the traditional side effects of drug therapy. This study is a first step in examining the effects of EVO game play on children with ADHD and comparing those effects to that of neuro-typical and age-matched participants. In this study we aim to show that EVO will be playable and interesting to ADHD children, shown by compliance during a short training phase; and that the interference processing ability and accompanying cognitive measures in ADHD children, as measured by EVO gameplay, will show a statistically significant deficit compared to age-matched neuro-typical children.

Section 2: Objective of the Study

2.1: Study Overview

This is an open-label study in children ages 8 to 12 to assess EVO cognitive game play. The study has two arms, children diagnosed with ADHD (ADHD who are off of medication) and neuro-typical children (controls). We plan to evaluate 80 participants (N = 40 per arm) in three site locations over a 29 day study period. The 29 day study period includes 2 in-clinic sessions and 27 days of out-patient game play. During the 27 days of out-patient game play, the participants will be instructed to play EVO 5 days per week for typically 30 minutes per day.

2.2: Primary Objective

The primary objective of this study is to demonstrate a statistically-meaningful difference in EVO cognitive measures between participants with ADHD diagnosis and participants without ADHD diagnosis. In other words, the study aims to show that EVO cognitive measures can demonstrate

sensitivity between ADHD and age-matched non-ADHD children. EVO cognitive measurements used to differentiate populations will be derived from the 20-minute gameplay in-clinic, as well as the gameplay measurements made in the first few days of at-home play, which may represent a more natural environment for collection of cognitive data.

When a participant plays the EVO game, the participant performs targeting and navigation tasks, which are similar in design to many commercial video games. EVO then determines the participant's ability to perform these individual tasks. The participant then engages in multitasking where the participant navigates and targets simultaneously. EVO then calculates the degradation in targeting ability due to navigation and the degradation in navigation ability due to targeting. These "Interference Costs/Deficits" are the basis of the cognitive measures. These measures along with play patterns are automatically captured by the game and wirelessly reported to an external data server.

The participants will play EVO in the lab on Day 0 and then take EVO home and be instructed to play it for typically 30 minutes per day for the next 27 days. On day 28 the participants will return to the lab and play EVO one last time. A co-primary objective of this study is to evaluate the safety of EVO game play based on treatment-emergent adverse events (TEAEs) that may occur during this 29 day period of time.

2.3: Secondary Objective

It's reasonable to assume that in order for EVO to ultimately have positive cognitive effects it must be engaging and interesting to the participants. A secondary objective of this study is to demonstrate that the participants play EVO throughout the 29 day study period. Specifically we want to demonstrate:

- Ability of ADHD and neuro-typical children to complete EVO in-clinic diagnostic assessment on day 0 and day 28
- Compliance (measure behavioral play patterns) of ADHD and neuro-typical children during out-patient EVO game training period, day 1 to day 27. ***Note: During the out-patient portion of the study, parents of participants that haven't played EVO over a 24 hour period will receive a communication from the study staff reminding them to ask their child to play the game.***

Also, as a co-secondary objective, we will collect qualitative game play feedback during the participant interviews on day 0 and day 28, in the in-clinic sessions. The questionnaire shown in Appendix D (Participant Questionnaire) will be used to capture the overall game experience. I.e. what did the participants like about EVO; what they disliked about EVO; and what they would suggest changing. This will allow us to create future game versions for larger scale studies that are optimized for engagement in this clinical population.

2.4: Exploratory Objectives

Throughout the study, EVO will be capturing and reporting the participants' data as they progress through the game, including participants' interference cost and single-task performance (reaction and visuomotor tracking tasks). During the in-clinic sessions on Day 0 and Day 28, in addition to playing EVO the participants will also complete a supplemental cognitive battery provided by Cambridge Cognition. This CANTAB ADHD Battery, as shown in Appendix G (CANTAB – ADHD), is a computer based assessment that provides motor control tasks, rapid visual information processing, spatial working memory, delayed matching, and stop signal tasks. Combined all five assessments take approximately 30 minutes to complete. After completing the CANTAB assessment, the participants will complete an additional 30 minute computer-based test for test of variable attention (TOVA). Finally, while the participants are performing the CANTAB or TOVA, during in-clinic sessions on Day 0 and Day 28, the parent / guardian will be asked to complete the Parent-Brief (See Appendix F). An exploratory objective of this study is to investigate any improvements in cognitive function (as measured by EVO, CANTAB, TOVA, and the BRIEF) that result from the 4 weeks of game training.

An additional exploratory endpoint is to perform multivariate data analysis on the host of EVO measures as well as CANTAB/TOVA/BRIEF supplemental cognitive measures to determine if any signatures may correlate with diagnostic status between ADHD and neuro-typical controls.

Section 3: Participant Selection

3.1: Inclusion Criteria

1) Age 8 to 12 at the time of parental informed consent.
2) Confirmed ADHD diagnosis at clinic (ADHD Cohort) per MINI-KID (See Appendix I, MINI-KID).
3) Baseline ADHD-RS-IV score >= 24 (ADHD Cohort), obtained at clinic (See Appendix H, ADHD-RS).
4) Baseline ADHD-RS-IV score <=13 (Neuro-typical Cohort), obtained at clinic (See Appendix H, ADHD-RS).
5) Consistently off ADHD drug for 1 week. Drugs include: Pre specified, oral psychostimulants (ADDERALL XR® [mixed salts of a single-entity amphetamine product], VYVANSE® [lisdexamfetamine dimesylate], CONCERTA® [methylphenidate HCl], FOCALIN XR® [dexmethylphenidate HCl], RITALIN LA® [methylphenidate HCl extended-release], METADATE CD® [methylphenidate HCl, USP], Strattera® (atomoxetine HCl), or FDA approved generic equivalents.
6) Consistently off Psychotropic drug for 1 month (Other than ADHD drug noted above).
7) Ability to follow written and verbal instructions (English).
8) Girls or Boys (Gender-matched – 30% girls minimum).
9) Functioning at an age-appropriate level intellectually.
10) Ability to comply with all the testing and requirements.

3.2: Exclusion Criteria

1) Current, controlled (requiring a restricted medication) or uncontrolled, comorbid psychiatric diagnosis with significant symptoms such as post-traumatic stress disorder, psychosis, bipolar illness, pervasive developmental disorder, severe obsessive compulsive disorder, severe depressive or severe anxiety disorder, conduct disorder, or other symptomatic manifestations that in the opinion of the Investigator that may confound study data/assessments (See Appendix I, MINI-KID).
2) Within the last 4 weeks, participant has entered or exited behavioral therapy. The participant should inform the Investigator if they intend to change their behavioral therapy during the 4 weeks of the study. Participants planning to enter therapy during the course of the study will be excluded.
3) Participant is currently considered a suicide risk in the opinion of the Investigator, has previously made a suicide attempt, or has a prior history of, or is currently demonstrating active suicidal ideation or self-injurious behavior (See Appendix L, C-SSRS).
4) History of failure to respond to an adequate trial of 2 treatments for ADHD (consisting of an appropriate dose and adequate duration of therapy and failure in efficacy in the opinion of the Investigator).
5) Motor condition that prevents game playing, as reported by parent or observed by investigator.
6) Recent history (within the past 6 months) of suspected substance abuse or dependence.
7) History of seizures (exclusive of febrile seizures), a tic disorder, significant tics, a current diagnosis of Tourette's Disorder.
8) Taken part in a clinical trial within 30 days prior to screening.
9) Diagnosis of or parent-reported color blindness.
10) Regular use of psychoactive drugs that in the opinion of the Investigator may confound study data/assessments.
11) Any other medical condition that in the opinion of the investigator may confound study data/assessments.

3.3: Concomitant Medication and Treatment Criteria

1) No use of psychotropic drugs.
2) No concomitant medications are permitted during the study with the exception of common over the counter (OTC) (eg. ibuprofen, acetaminophen) and prescription medications (eg. antibiotics) for minor transient ailments. Non-sedating antihistamines are allowed. Families should advise the study staff if it becomes necessary to use other types of medication. Study investigators can approve short-term use of other medications that are not anticipated to confound study assessments.
3) No initiation or change in psychotherapy or any other non-pharmacological treatment for ADHD or mental health.

3.4: Participant Selection

Recruitment of participants will be managed by the Investigators upon approval from the Institutional Review Board (IRB). The study may be posted in newspapers (including free ones), and on Internet websites, to reach an economically and socially diverse population. Radio advertising is also acceptable.

44 children ages 8 to 12 will be recruited into each of the two arms of the study to achieve 40 evaluable participants per arm (assuming a 10% drop-out rate) and 88 participants in total. Of the 88 participants recruited in total, 66 participants will be recruited by the Florida Clinical Research Center, LLC and 22 participants will be recruited by Duke University.

The neuro-typical (control) cohort should have no prior diagnosis of ADHD. The ADHD cohort with a diagnosis of ADHD should report that that they have been consistently off of ADHD medication for no less than 1 week. Any medication changes during the trial should be discussed with the Investigator in advance

The study sites (Florida Clinical Research Center, LLC and Duke University) will use their best efforts to gender-match and enroll a minimum of 30% girls. The sites will maintain enrollment logs and direct recruitment to try to achieve this goal. However no procedure will be implemented to pause or suspend the recruitment of boys if the 30% goal for girls isn't achieved.

The participant age range will be divided into two groups. The first group comprises children 8 to 10 years of age (at time of informed consent) and second group comprises children 11 to 12 years of age (at time of informed consent). The study sites (Florida Clinical Research, LLC and Duke University) will maintain enrollment logs that will direct recruitment to achieve an age-match balance between the two groups. An imbalance of more than 70% (or less than 30%) will be proactively corrected as follows:

> Once 60% of the participants are enrolled in the study (36th participant FLCRC / 12th participant Duke), the sites will implement a one-time corrective action if there is an imbalance greater than 30% / 70% between the two groups. To correct an imbalance greater than 30% / 70%, the site(s) will exclusively recruit into the minority group until the minimum 30% (18 minority group participants FLCRC / 6 minority group participants Duke) level is achieved.

Section 4: Participant Enrollment

4.1: Methods of enrollment

All participants who sign a Child Assent Form (See Appendix C, CAF) and whose parent or legal guardian sign an Informed Consent Form (See Appendix B, ICF) and are screened will be documented on a screening log. All participants who qualify at the screening portion of the Day 0 visit and who are

enrolled in the study will be assigned a participant number and documented on the enrollment log. A note will be made in the source documentation verifying that the participant and parent have willingly signed the CAF and ICF, respectively, prior to participation in any study procedures.

Estimated sample size is 80 evaluable participants as defined by participants who complete the screening process and meet the inclusion and exclusion criteria on the Day 0 visit. Anticipating screen failures and dropouts, we will recruit 88 participants.

4.2: Informed Consent / Child Assent Form (ICF/CAF)

The Investigator or designee will inform the potential study participant and their parent/legal guardian of all aspects of the study and answer their questions. If the participant and parent/legal guardian agree to study participation they will document their consent in writing by signing the CAF and ICF respectively (See Examples in Appendix B, ICF and Appendix C, CAF). The Investigator is responsible for using CAF and ICF forms that have been approved by the IRB and are the most current version. If new versions of these forms are approved by the IRB, while a participant is in the treatment portion of the study, then the Investigator or designee must inform the participant and parent/legal guardian of the changes and, if the participant and parent/legal guardian agrees to continue treatment, they should sign the updated forms.

4.3: Treatment Assignment

Eligible participants will be recruited into the trial without randomization. All participants in both cohorts (ADHD and Neuro-typical/controls) will receive the same assessments (ADHD-RS, C-SSRS, MINI-Kid, CANTAB ADHD Battery, TOVA, and Parent-BRIEF) and play EVO over the 29 day study period (2 in-clinic play sessions and typically 30 minutes per day, 5 days per week, for 27 days at home).

4.4: Participant Withdrawal

Participants who end participation in this study for reasons other than those defined by this protocol will be considered withdrawals. Participant will be discontinued from the study if a breach of set inclusion/exclusion criteria and/or protocol procedures occurs. At any time after enrollment, a participant may be discontinued. Reasons for discontinuation of a participant from the study will include, but may not be limited to, the following:

1) Intolerance to a required study procedure at any time point.
2) Noncompliance with protocol restrictions and requirements.
3) The occurrence of a serious adverse experience at any time point.
4) Intercurrent illness that would, in the judgment of the investigator, affect assessments of clinical status to a significant degree.
5) Participant or parent/legal guardian's request to withdraw.

When a participant or parent/legal guardian decides to end participation in the study, attempts will be made to have the participant come back for the scheduled end-of-study observations (Day 28 in-clinic

visit). If that is not possible, attempts will be made to contact the participant's parent/legal guardian to obtain information about the reasons for discontinuation and any adverse events and to arrange for the return of the iPad.

Section 5: Study Procedures

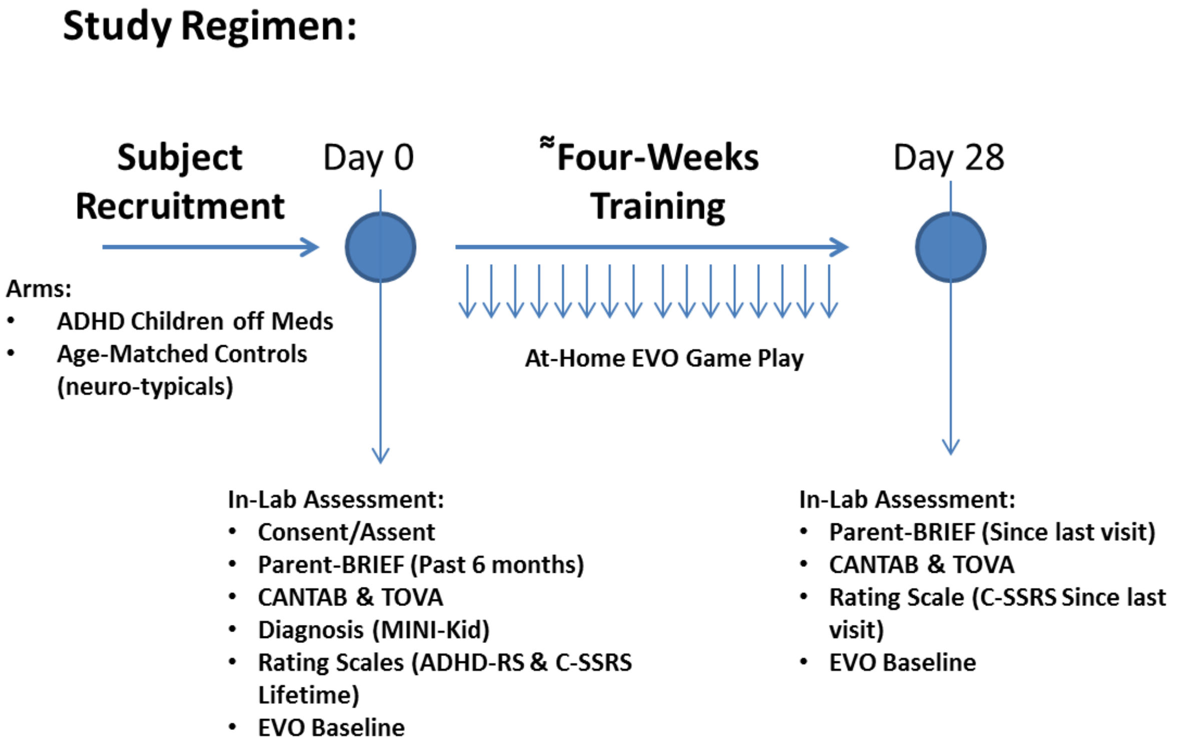

5.1: Study Visits and Procedures

Study procedures will take place at the Screening Visit & Baseline Visit (Day 0), the At-Home Play Period (Day 1 to Day 27), and at the Baseline & Exit Visit (Day 28). After the participant signs the child assent form (See Appendix C, CAF) and the parent/legal guardian signs the informed consent form (See Appendix B, ICF) and the following procedures will take place:

Study Flow Chart

Period	Screening & Baseline	At-Home Training	Exit
Visit	1	-	2
Day	0	1 - 27	28
Informed consent and assent	X		
Demographic & contact information	X		
Parent BRIEF	X		X
Supplemental cognitive battery (CANTAB & TOVA)	X		X
MINI-Kid diagnosis	X		
ADHD-RS rating	X		
C-SSRS rating	X		X
EVO iPad baseline cognitive data	X		X
At-home EVO iPad cognitive training		X	
Feedback sessions and questionnaires	X		X
Safety assessments /AEs and concomitant medication / therapy	X		X
Measure behavioral play patterns		X	
Collect iPads			X

5.1.1: Day 0 In-clinic Screening & Baseline Procedure

In order to minimize noise in the resulting data, all participants are to be screened in the afternoon and in general during the time that corresponds to the end of the school day.

Parental informed consent and child assent (See Examples in Appendix B, ICF and Appendix C, CAF) must first be obtained prior to any further evaluation.

If the parent/legal guardian or a potential participant inquires about EVO and Akili Interactive Labs, Inc. the inquiring party may receive a copy of the company executive summary (See Appendix E).

<u>For participants electing to participate in the study the following screening and baseline assessments will be performed:</u>

1) The participant will be assigned a participant number as follows:
Akili Study Number = “Akili-001”
Akili Participant Numbers = “001” to “066” (reserved for Florida Clinical Research Center)
Akili Participant Numbers = “101” to “122” (reserved for Duke University

2) Parent/legal guardian contact information will be collected. This includes parent name, participant name, home address, home phone number, cell phone number, and email address. This information

will remain at the study site as source documentation only and will not be entered into the case report form. This information will only be used by the clinical site in the event the site needs to contact the parent/legal guardian due to lack of game play compliance or to remind them return to the clinic for the Day 28 exit visit and/or to return the iPad.

3) Demographic information will be collected including: Age, Gender, Ethnicity (optional), Grade Level, and Grade Point Average (If available). All demographic information will be recorded in the case report form; no participant identifiable data will be recorded.

4) Instruct the participants to perform the CANTAB-ADHD supplemental cognitive battery for approximately 30 minutes to establish their baseline performance. The study staff will need to interact with the participants to ensure that they execute the CANTAB battery correctly. ***Note: Refer to the CANTAB training materials provided with the CANTAB tablet based systems.*** **Also:**

- ***Note: It's important NOT to connect the CANTAB tablet based systems to Wi-Fi. This will negatively impact system performance.***
- Following completion of the CANTAB-ADHD battery follow the CANTAB download instructions and save the participants baseline assessment data on a memory stick device. The two downloaded files should be named: "**001_###_A_S.csv"** (where **"001"** refers to the study number, **"###"** refers to the participant number, **"A"** refers to the fact that this is the Day 0 baseline assessment, and **"S"** refers to the CANTAB summary data sheet. AND "**001_###_A_D.csv"** (where **"001"** refers to the study number, **"###"** refers to the participant number, **"A"** refers to the fact that this is the Day 0 baseline assessment, and **"D"** refers to the CANTAB detailed data sheet.
- Backup the memory stick files to a dedicated Akili-001 folder on an in-clinic computer/server at the end of the day.
- Indicate in the case report form that the CANTAB assessment has been successfully completed.

5) Instruct the participants to perform the TOVA supplemental cognitive battery for approximately 30 minutes to establish their baseline performance. The investigator or study staff will need to interact with the participants to ensure that they execute the TOVA battery correctly. ***Note: Refer to the TOVA training materials provided with the TOVA software.***

- Following completion of the TOVA assessment, follow the TOVA download instructions and save the participants baseline assessment data on a memory stick device.
- Backup the memory stick files to a dedicated Akili-001 folder on an in-clinic computer/server at the end of the day.
- Indicate in the case report form that the TOVA assessment has been successfully completed.

6) While the participant is conducting CANTAB or TOVA assessments, instruct the parent/legal guardian to complete the Parent-BRIEF survey. See Appendix F (Parent-BRIEF). The study staff should complete the Parent Form Scoring Summary (Provided with the Parent-BRIEF materials) including, the raw score, the T score, the %ile, the negativity score, and the inconsistency score. Record in the case report form the raw score, T score, and %ile for Inhibit, BRI, Working Memory, MI and GEC. Also record the negativity and inconsistency scores.

7) A clinical diagnosis per the MINI-Kid assessment (See Appendix J) will be performed and medication status will be collected. The diagnosis will verify onset, impairment, and pervasiveness of ADHD for the participants in the ADHD cohort. The resulting diagnostic output will be recorded in the case report form; no participant identifiable data will be recorded. ***Note: Separate instructions will be provided with the MINI-Kid assessment questionnaire. Also sections B, V, W, and X are omitted.***

8) The ADHD-RS-IV assessment (See Appendix H) will be performed along with the C-SSRS lifetime assessment (See Appendix L). The resulting scores will be recorded in the case report form; no participant identifiable data will be recorded. Neuro-typical control participants with ADHD-RS scores <=13 will be eligible to be enrolled into the study. ADHD participants with ADHD-RS scores >= 24 will be eligible to be enrolled into the study.

9) The investigator will determine if all inclusion and exclusion are met and record this finding in the case report form; no participant identifiable data will be recorded.

<u>For participants continuing in the study the following will be performed:</u>

1) Allow the participant to take a 15 minute break with an optional snack. ***Note: No caffeine containing beverages are allowed.***

2) Instruct the participant to play EVO for approximately 20 minutes to establish the participant's baseline performance. The participant will perform:

- One EVO practice session
- One EVO diagnostic/assessment session
- One EVO training session

The investigator or study staff will need to interact with the participant to ensure the participant understands the rules and instructions of the game. ***Note: Follow the instructions in Appendix K (EVO Game Play Instructions for the Investigator) to reset and run EVO on the iPad for a new participant.* Also:**

- Record the Akili serial number (printed on the back of the device) in the case report form.
- Indicate in the case report form that the EVO baseline sessions have been successfully completed.

3) Throughout the Day 0 in-lab visit perform a safety assessment and capture any Adverse Events (AEs) in the case report form. In the event of an AE immediately notify the investigator.

4) Study staff will complete the participant game experience questionnaire (See Appendix D) and his/hers' observation questionnaire (See Appendix M). Indicate in the case report form that the questionnaires are completed.

5) Instruct the parent/legal guardians that during the next 27 days (<u>and starting tomorrow</u>) their child should play EVO at least <u>5 days a week</u> for typically <u>30 minutes a day</u>. And that this is accomplished by playing 7 consecutive runs of approximately 4 minutes each. Notify them that sometimes the game may ask their child to repeat an internal diagnostic/assessment

which may take an additional 15 minutes. Notify them that the game will automatically "lock" after 7 runs in any given day, and that participants will not be able to play until the following day. ***Note: If their child becomes fatigued or frustrated with the game after 30 minutes of play, in any given day, he or she can turn the game off and it will automatically pick up where it left off during the next play session.***

6) Send the parent/legal guardian and participant home with the following:

- A copy of their signed informed consent (See Example in Appendix B)
- A copy of their signed child assent (See Example in Appendix C)
- The iPad device with EVO loaded on it
- A copy of the EVO Game Play Instructions for the Parent and Participant (See Appendix J)

5.1.2: Day 1 to Day 27 at home EVO play procedure

During the next 27 days, and at a time of day preferable to the participant and parent, the participant will play EVO at least 5 days a week for typically 30 minutes a day. This will be accomplished by playing 7 consecutive runs of approximately 4 minutes each.

Compliance will be tracked as all game data is automatically collected real-time on a central cloud server that preserves participant anonymity.

Akili will notify the study site as to any participant number that failed to play EVO over a 24-hour period. To further protect anonymity, only the study site will then reach out to participants and parents to remind them to play (by email and/or text message). The message will read "Subject: EVO Game-play Follow-up. Text: Dear parent or guardian, Your child who is currently participating in the EVO game play clinical study, has either not played EVO for over 24 hours or the iPad provide to you is not connected to your home WIFI router. Please check your wireless connection and remind your child to play EVO. If you have any questions, please respond directly to this message. Sincerely, <Study Staff Name>"

On day 21 the study site should remind the parents by phone, preferably (email and/or text are also acceptable), that their child is entering the last week of the study. The message will read "Subject: EVO Game-play Follow-up. Text: Dear parent or guardian, Tomorrow your child is entering the final week of the EVO game play clinical study. It's important that your child plays EVO for at least 5 more days prior to returning to the clinic on <Insert Date>. Please remember to bring your iPad with you when you return to our clinic. If you have any questions, please respond directly to this message. Sincerely, <Study Staff Name>. If a phone call is made the same message should be conveyed.

5.1.3: Day 28 In-clinic Baseline & Exit Procedure

In order to minimize noise in the resulting data, all participants returning to the clinic for their exit visit should do so in the afternoon and in general during the time that corresponds to the end of the school day. ***Note: If returning exactly on Day 28 is not possible for the parent /legal guardian, Day 28 +3 days is acceptable.***

<u>The following baseline assessments will be performed:</u>

1) Instruct the participants to perform the CANTAB-ADHD supplemental cognitive battery for approximately 30 minutes to establish their baseline performance. The study staff will need to interact with the participants to ensure that they execute the CANTAB battery correctly. ***Note: Refer to the CANTAB training materials provided with the CANTAB tablet based systems.*** **Also:**

- ***Note: It's important <u>NOT</u> to connect the CANTAB tablet based systems to Wi-Fi. This will negatively impact system performance.***
- Following completion of the CANTAB-ADHD battery follow the CANTAB download instructions and save the participants baseline assessment data on a memory stick device. The two downloaded files should be named: "**001_###_B_S.csv"** (where **"001"** refers to the study number, **"###"** refers to the participant number, **"B"** refers to the fact that this is the Day 28 baseline assessment, and **"S"** refers to the CANTAB summary data sheet. AND "**001_###_B_D.csv"** (where **"001"** refers to the study number, **"###"** refers to the participant number, **"B"** refers to the fact that this is the Day 28 baseline assessment, and **"D"** refers to the CANTAB detailed data sheet.
- Backup the memory stick files to a dedicated Akili-001 folder on an in-clinic computer/server at the end of the day.
- Indicate in the case report form that the CANTAB assessment has been successfully completed.

2) Instruct the participants to perform the TOVA supplemental cognitive battery for approximately 30 minutes to establish their baseline performance. The investigator or study staff will need to interact with the participants to ensure that they execute the TOVA battery correctly. ***Note: Refer to the TOVA training materials provided with the TOVA software.***

- Following completion of the TOVA assessment, follow the TOVA download instructions and save the participants baseline assessment data on a memory stick device.
- Backup the memory stick files to a dedicated Akili-001 folder on an in-clinic computer/server at the end of the day.
- Indicate in the case report form that the TOVA assessment has been successfully completed.

3) While the participant is conducting CANTAB or TOVA assessments, instruct the parent/legal guardian to complete the Parent-BRIEF survey. ***Note: The parent/legal guardian should focus his/hers' assessment on the <u>past month's</u> behavior***. See Appendix F (Parent-BRIEF). The study staff should complete the Parent Form Scoring Summary (Provided with the Parent-BRIEF materials) including, the raw score, the T score, the %ile, the negativity score, and the inconsistency score. Record in the case report form the raw score, T score, and %ile for Inhibit, BRI, Working Memory, MI and GEC. Also record the negativity and inconsistency scores.

4) The C-SSRS since last visit exit assessment (See Appendix L) will be performed. The resulting score will be recorded in the case report form; no participant identifiable data will be recorded.

5) Allow the participant to take a 15 minute break with an optional snack. ***Note: No caffeine containing beverages are allowed.***

6) Instruct the participants to play EVO for approximately 20 minutes to establish their baseline performance. ***Note: Follow the instructions in Appendix K (EVO Game Play Instructions for the Investigator) to run EVO during the Day 28 visit***. Indicate in the case report form that EVO has been successfully played for 20 minutes.

7) Study staff will complete the participant game experience questionnaire (See Appendix D). Indicate in the case form that the questionnaire is completed.

8) Instruct the parent/legal guardians to complete the parent/guardian game experience questionnaire (See Appendix N). Indicate in the case form that the questionnaire is completed.

9) Throughout the Day 28 in-lab visit perform a safety assessment (For the take home period and Day 28 visit) and capture any Adverse Events (AEs) in the case report form. In the event of an AE immediately notify the investigator. Also document in the case report form any reported concomitant medications or non-pharmacological treatment for ADHD or mental health.

10) Collect the iPad and give the parent/legal guardian their stipend check.

5.2: Study Product

EVO Cognitive Training Mobile Video Application developed by Akili Interactive Labs, 500 Boylston Street, Suite 1600, Boston MA, 02116.

5.3: Participant Reimbursement

We do not expect there to be any costs to the participant for participating in this study. The participant will not be paid to participate in this research study. The parent/legal guardian will be provided with $300 in maximum total compensation for time and travel to the study clinic ($50 at Screening/Baseline visit, $200-maximum for the at home game play and $50 for Exit visit). If the iPad isn't returned at the end of the study, the compensation will be forfeited.

Section 6: Data Management and Analysis

6.1: Data Collection and Transfer

Study data to be shared with Akili will be collected at the Screening Visit & Baseline Visit (Day 0), the At-Home Play Period (Day 1 to Day 27), and at the Baseline & Exit Visit (Day 28). All shared data will be delivered electronically to Akili.

Note: Data collected on the CANTAB systems should be backed up to a memory stick on a weekly basis. Follow the CANTAB instructions for creating a backup ".zip" file.

Note: Data collected with the TOVA software should be backed up to a memory stick on a weekly basis. Follow the TOVA instructions for backing up files.

After the participant signs the child assent form (See Appendix C, CAF) and the parent/legal guardian signs the informed consent form (See Appendix B, ICF) the following data will be collected:

<u>Day 0 In-clinic Screening & Baseline Data</u>

- Demographic information including: Age, Gender, Ethnicity (optional), Grade Level, and Grade Point Average (If available)
- CANTB-ADHD supplemental cognitive battery assessment
  Note: Following the Day 0 visit, the downloaded CANTAB files named: "001_###_A_S.csv" AND "001_###_A_D.csv" should be emailed to Akili (address TBD)
- TOVA supplemental cognitive battery assessment (Files TBD)
  Note: Following the Day 0 visit, the downloaded TOVA flies should be emailed to Akili (address TBD)
- Parent-BRIEF survey (See Appendix F)
- Clinical diagnosis per MINI-Kid (See Appendix I) and medication status
- Clinical assessments per ADHD-RS-IV (See Appendix H) and C-SSRS lifetime (See Appendix L)
- EVO play data
- Safety assessment and any Adverse Events (AEs)
  Note: Any AEs should be reported to Akili within 24 hours
- Completed participant questionnaire (See Appendix D) and study staff observation of game-play questionnaire (See Appendix M)
  Note: Following the Day 0 visit, a scanned copy of the two questionnaires should be emailed to Akili (address TBD)

<u>Day 1 to Day 27 at home EVO play data</u>

- EVO play data

<u>Day 28 In-clinic Baseline & Exit Procedure</u>

- CANTB-ADHD supplemental cognitive battery assessment
  Note: Following the Day 28 visit, the downloaded CANTAB file named: "001_###_B_S.csv" AND "001_###_B_D.csv" should be emailed to Akili (address TBD)
- TOVA supplemental cognitive battery assessment (Files TBD)
  Note: Following the Day 28 visit, the downloaded TOVA flies should be emailed to Akili (address TBD)

- Parent-BRIEF survey (See Appendix F)
- Clinical assessments per C-SSRS since last visit (See Appendix L)
- EVO play data
- Safety assessment and any Adverse Events (AEs) along with any reported concomitant medications or non-pharmacological treatment for ADHD or mental health. ***Note: Any AEs should be reported to Akili within 24 hours***
- Completed participant questionnaire (See Appendix D) and the parent or guardian questionnaire (See Appendix N)
  Note: Following the Day 28 visit, a scanned copy of the two questionnaires should be emailed to Akili (address TBD)
- Completed case report form
  Note: Following the Day 28 visit, a scanned copy of the case report form should be emailed to Akili (address TBD)

6.2: Statistical Analysis

This study will be powered to detect a difference in EVO derived cognitive measures (interference cost) between neuro-typicals and ADHD participants. Power calculations (0.9, alpha = 0.05) are designed to detect a prevalence of impairment in an ADHD population of at least 40%, assuming a prevalence of impairment in the control group of 10% (assuming normal distribution for each group). Difference between groups will be determined using a two-sided t-test to compare specific groups with a threshold of $p < 0.05$, and group by variable interactions will be assessed with standard ANOVA.

An additional objective/outcome of this study is to demonstrate that EVO is "playable" by the ADHD population. This is a qualitative measure and no statistics will be applied.

As an exploratory measure, multivariate analysis will be used to compare and explore ADHD cognitive signatures, as measured by EVO (Day 0 to Day 28) to that of neuro-typical controls.

Section 7: Risk/Benefit

The risks to a participant participating in this study are very small. The study involves completing some computerized tests and games and answering some questions. It is possible that the participant could become frustrated by some of the tasks. The participant could become fatigued by the computer tests or game play. If the participant becomes frustrated or fatigued, they may stop at any time. There are no other risks to taking part in this study of which we are aware.

The participant will not receive any direct benefit from participating in this study. No promise can be made concerning the study outcome, because results from a clinical research study cannot be predicted.

Section 8: Monitoring and Quality Assurance

8.1: Monitoring of Source Data

The study monitor will review the progress of the study to ensure proper study conduct and accurate data collections. Periodic reviews (monthly) of case report forms, clinical records, and administrative documents will take place. Additionally, Akili or its representative will conduct monitoring visits at site initiation, two or three times during study conduct, and at study closeout. The study's data will also be made available to Akili, or its representative. As this is and open label study, Akili will perform data analysis concurrently with study conduct.

8.2: Safety Monitoring

Participants will be screened for eligibility at which time a clinical diagnosis (MINI-Kid) along with medication status will be collected. The diagnosis will verify onset, impairment, and pervasiveness of ADHD for the participants in the ADHD cohort. Additionally, the ADHD-RS and C-SSRS assessments (See Appendix H and L respectively) will be performed. Participants who do not fulfill the inclusion criteria will not be enrolled. Participants whose histories are outlined in the exclusion criteria will also not be enrolled. Evaluations will be ongoing throughout the study to detect adverse events and changes in existing medical conditions.

At any time after enrollment, a participant may be discontinued. Reasons for discontinuation of a participant from the study are described in Section 4.4 of this protocol.

Substantive changes in the protocol include alterations that affect the safety of participants or that alter the scope of the investigation, the scientific quality of the study, the experimental design, study product, the number of participants treated, or the participant selection criteria. If needed, these changes will be initiated via formal written protocol amendment. This protocol amendment must be reviewed and approved by Akili and the investigators prior to implementation. If a protocol amendment results in changes to the informed consent form, the revised form must be approved prior to implementation by Akili. Emergency departures from the protocol that eliminate an apparent immediate hazard to a particular participant and that are deemed crucial for the safety and well-being of that participant may be instituted for that participant only. The investigator will contact Akili as soon as possible in the case of such a departure and in no case will notification be withheld more than 3 days. Although these departures do not require pre approval by Akili, Akili must be notified in writing according to specified guidelines after the departure has been made. Furthermore, the investigator will document in the participant's case report form the reasons for the departure from the protocol and the ensuing events.

8.3: Outcomes Monitoring

The study will be monitored by the principal investigator and members of the study staff, as well as Akili. Given the short duration of the study and the extremely low risk associated with this protocol, no Data Safety Monitoring Board will be convened. All data relevant to the assessments outlined in this protocol will be recorded in the case report form.

8.4: Protection of Participants

This study will be governed by the following directives and guidelines:

- Declaration of Helsinki (Edinburgh 2000)
- ICH Guidelines for Good Clinical Practice (ICG-E6)
- The Code of Federal Regulations Title 21 Parts 50, 54,56, 312, 314

In addition, the study protocol and any protocol amendments will be reviewed by the appropriate local ethics committee. Participants will be informed both verbally and in writing about the nature of the study, the anticipated benefits and risks involved. They will be instructed about their right to discontinue their participation at any time without prejudice or jeopardy to future medical care. They must confirm their consent (parents/legal guardian) and assent (participant) to participate in writing prior to the initiation of any screening procedures. The ICF and CAF must be reviewed and approved by the IRB prior to use.

8.5: Adverse Events (AEs)

Adverse Event (AE) is any untoward medical occurrence in a participant that does not necessarily have a causal relationship with this treatment. An AE can therefore be any unfavorable and unintended sign, symptom, or disease temporally associated with the use of a study product, whether or not related to the study product.

Serious Adverse Event (SAE) is any untoward medical occurrence that:

- Results in death
- Is life-threatening
- Requires inpatient hospitalization or prolongation of existing hospitalization
- Results in persistent or significant disability or incapacity
- Is another medically important condition

All adverse events that occur after informed consent should be documented as an AE. The Investigator should ensure that all adverse events that occur during the study period are recorded. All AEs should be followed until resolution or until, in the Investigator’s judgment, they are chronic and stable. If an emergency situation should occur, appropriate medical measures should be taken to stabilize the participant.

Documentation of AEs includes: date and time of onset and resolution of AE, intensity, frequency, seriousness, related interventions and outcome. The Investigator must also evaluate the probability of a causal relationship of the AE to the study treatment as being: “definite, probable, possible,

unlikely, or unrelated." Intensity of adverse events will be graded as mild, moderate, or severe according to the following criteria:

- Mild: symptoms that are easily tolerated and transient in nature with minimal or no impairment of normal activity
- Moderate: symptoms that are poorly tolerated, are sustained, and interfere with normal activity
- Severe: symptoms that are incapacitating and render the participant unable to go to school or participate in many or all usual activities

All SAEs will be reported to the IRB according to the IRB's requirements, regardless of causality. They will also be reported to Akili according to regulatory guidelines.

This page is intentionally left blank

Appendix A (Protocol Synopsis)

ADHD Pilot Feasibility Study - Protocol Synopsis
July, 2013

Study title	A study to assess the feasibility of EVO, game-play, to engage participants with Attention Deficit Hyperactivity Disorder (ADHD) and to evaluate interference cost in children ages 8 to 12 years old with ADHD compared to age-matched controls.
Sponsor	Akili Interactive Labs, Inc., 500 Boylston Street, Suite 1600, Boston, MA 02116
Project Code	Akili-001
Study Phase	Exploratory Feasibility Pilot
Study Product	"EVO" Revision 01, Cognitive Assessment and Measurement Game
Study Start Date	August, 2013
Study Centers	Number of Centers = 3
Background	Akili's therapeutic game, EVO, is based on the work of Prof. Adam Gazzaley from UCSF, who has identified a way to measure and improve the ability to process cognitive interference (essentially, interruptions and distractions). Interference susceptibility (measured by a proprietary "Interference Cost") is recognized as a limiting factor across global cognitive function (including attention and memory) and is known to be fragile in multiple diseases. The basic scientific mechanisms underlying our first game have been tested in controlled (N=203) studies of healthy participants across a wide age range (8 to 70 years of age) to validate the ability to detect differences between age groups and to enhance interference processing in older adults. In this pilot study, Akili desires to determine if EVO is playable and engaging to the disease cohorts; and the degree to which children with a confirmed ADHD diagnosis show cognitive deficits, as measured by EVO, as compared to an age-matched neuro-typical cohort.
Hypothesis	EVO will be playable and interesting to ADHD children, shown by compliance during a short training phase. The interference processing ability and accompanying cognitive measures in ADHD children, as measured by EVO gameplay, will show a statistically significant deficit compared to age-matched neuro-typical children.
Primary Objective<br><br><br><br><br><br>Secondary Objective	<u>Interference Cost Comparison (Degree to which children with ADHD show cognitive deficits as measured by EVO as compared to age-matched controls</u><br>• Demonstrate a difference of ADHD participants' initial interference deficit, as measured by EVO from that of neuro-typicals.<br><u>Safety</u><br>• Evaluate the safety of EVO game play based on treatment-emergent adverse events (TEAEs)<br><br><u>EVO Play Feasibility (Is EVO playable and engaging to the disease cohorts?)</u><br>• Demonstrate the ability of ADHD and neuro-typical children to complete

	EVO in-clinic diagnostic assessment (DAY 0)<br>• Demonstrate compliance (measure behavioral play patterns) of ADHD and neuro-typical children during out-patient EVO game training period (DAY 1 to DAY 27)<br>• Demonstrate the ability of ADHD and neuro-typical children to complete in-clinic EVO diagnostic assessment (DAY 28)<br>• Collect qualitative game feedback during assessment and training (DAY 0 to DAY 28)
Exploratory Objectives	<u>Comparison of Interference Cost detected by EVO to Supplemental Cognitive Battery Results</u><br>• Assess the relationship between the CANTAB (ADHD Battery), TOVA, Parent-BRIEF, and EVO play cognitive measurements<br>• investigate any improvements in cognitive function (as measured by EVO, CANTAB, TOVA, and the BRIEF) that result from the 4 weeks of game training<br><br><u>Multivariate / Combinational Signatures TBD</u><br>• Compare ADHD cognitive signatures, as measured by EVO (DAY 0 to DAY 28) to that of neuro-typical controls
Study Design<br><br><br><br><br><br><br>Criteria	<u>Overview</u><br>• Open-label<br>• 2-arm study (neuro-typical controls & ADHD and off-medication)<br>• N = 80 evaluable participant (N = 40 per arm)<br>• Number of sites = 3<br>• Duration = 29 days<br><br><u>Inclusion</u><br>• Age 8 to 12 at the time of parental informed consent.<br>• Confirmed ADHD diagnosis (ADHD Cohort) at clinic (per MINI-KID)<br>• Baseline ADHD-RS-IV score >= 24, obtained at clinic (ADHD Cohort)<br>• Baseline ADHD-RS-IV score <=13, obtained at clinic (Neuro-typical Cohort)<br>• Consistently Off ADHD drug for 1 week. Drugs include: Pre specified, oral psychostimulants (ADDERALL XR® [mixed salts of a single-entity amphetamine product], VYVANSE® [lisdexamfetamine dimesylate], CONCERTA® [methylphenidate HCl], FOCALIN XR® [dexmethylphenidate HCl], RITALIN LA® [methylphenidate HCl extended-release], METADATE CD® [methylphenidate HCl, USP], Strattera® (atomoxetine HCl), or FDA-approved generic equivalents)<br>• Consistently off Psychotropic drug for 1 month (Other than ADHD drug noted above)<br>• Ability to follow written and verbal instructions (English)

	• Girls or Boys (Target = Gender-matched – 30% girls minimum)<br>• Functioning at an age-appropriate level intellectually.<br>• Ability to comply with all the testing and requirements.<br><br><u>Exclusion</u><br>• Current, controlled (requiring a restricted medication) or uncontrolled, comorbid psychiatric diagnosis with significant symptoms such as post-traumatic stress disorder, psychosis, bipolar illness, pervasive developmental disorder, severe obsessive compulsive disorder, severe depressive or severe anxiety disorder, conduct disorder, or other symptomatic manifestations that in the opinion of the Investigator that may confound study data/assessments.<br>• Within the last 4 weeks, participant has entered or exited behavioral therapy. The participant should inform the Investigator if they intend to change their behavioral therapy during the 4 weeks of the study. Participants planning on entering therapy during the course of the study will be excluded.<br>• Participant is currently considered a suicide risk in the opinion of the Investigator, has previously made a suicide attempt, or has a prior history of, or is currently demonstrating active suicidal ideation or self-injurious behavior.<br>• History of failure to respond to an adequate trial of 2 treatments for ADHD (consisting of an appropriate dose and adequate duration of therapy and failure in efficacy in the opinion of the Investigator).<br>• Motor condition that prevents game playing as reported by the parent or observed by the investigator.<br>• Recent history (within the past 6 months) of suspected substance abuse or dependence.<br>• History of seizures (exclusive of febrile seizures), a tic disorder, significant tics, a current diagnosis of Tourette's Disorder.<br>• Taken part in a clinical trial within 30 days prior to screening.<br>• Diagnosis of or parent-reported color blindness<br>• Regular use of psychoactive drugs that in the opinion of the Investigator may confound study data/assessments.<br>• Any other medical condition that in the opinion of the investigator may confound study data/assessments.<br><br><u>Concomitant Medication and Treatment</u><br>• No use of psychotropic drugs.<br>• No concomitant medications are permitted during the study with the exception of common over the counter (OTC) (eg. ibuprofen, acetaminophen) and prescription medications (eg. antibiotics) for minor transient ailments. Non-sedating antihistamines are allowed. Families should advise the study staff if it becomes necessary to use other types of medication. Study investigators can approve short-term use of other medications that are not anticipated to confound study assessments.<br>• No initiation or change in psychotherapy or any other non-pharmacological treatment for ADHD or mental health.

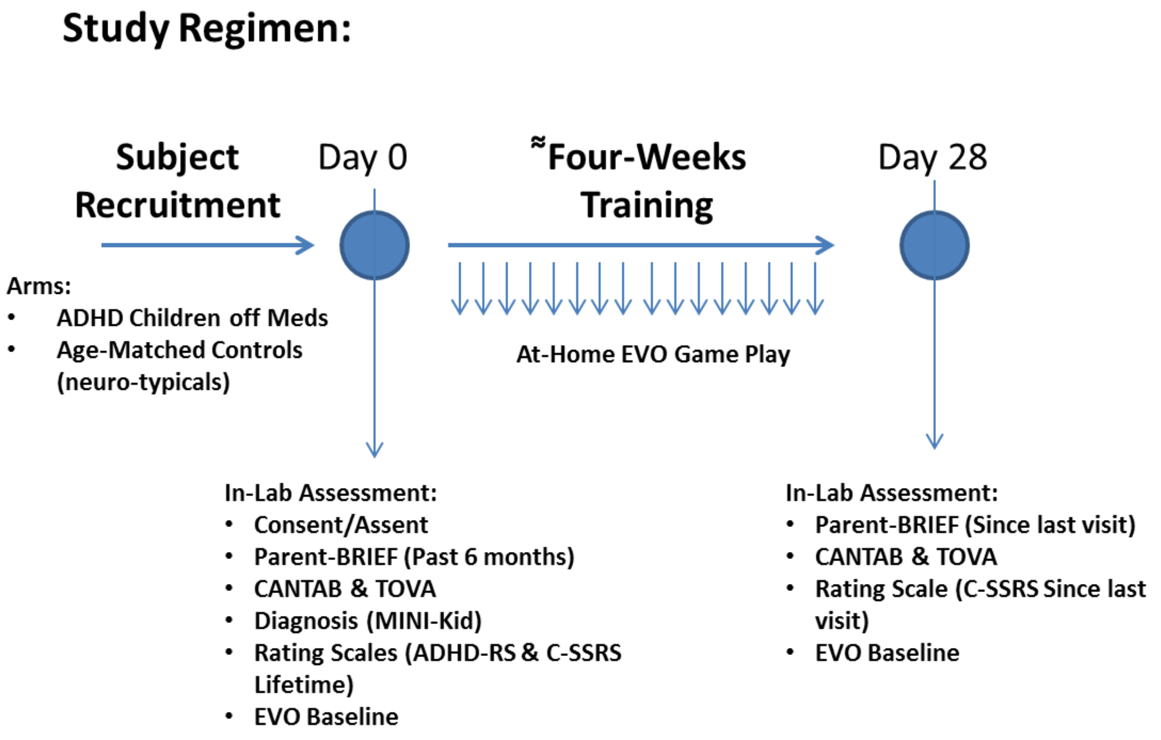

Study Protocol

Recruitment

44 children ages 8 to 12 will be recruited into each of the two arms of the study to achieve 40 evaluable participants per arm (assuming a 10% drop-out rate) and 88 participants in total.

The neuro-typical (control) cohort should have no prior diagnosis of ADHD. The ADHD cohort should have a physician diagnosis of ADHD and they should report that that they have been consistently off of ADHD medication, and stable for no less than 1 month. Any medication changes during the trial should be discussed with the Investigator in advance.

Afternoon Screening & Baseline – In-clinic (DAY 0)

- Parental informed consent will be obtained and demographic, diagnosis status (MINI-Kid), and medication status will be collected in a questionnaire. ADHD-RS and C-SSRS assessments will be performed on the study participants. Neuro-typical control participants with ADHD-RS scores <=13 will be eligible to be enrolled into the study. ADHD participants with ADHD-RS scores >= 24 will be eligible to be enrolled into the study.
- The participants will play EVO for approximately 20 minutes in the clinic to establish their baseline performance. In addition a supplemental cognitive battery (CANTAB) and TOVA will be conducted along with the Parent-

	BRIEF survey.<br>• A safety assessment (AEs) and will be recorded.<br>• Questionnaire will be completed to collect participant feedback on their game experience and the study staff's observations.<br>• Participants will be sent home with EVO on an iPad and they will be instructed to play EVO over the next four weeks for 30 minutes per day and at least 5 days per week during the training period.<br><br>*Anytime* Training – At-Home (DAY 1 to DAY 27)<br>• The participants will play EVO for 30 minutes per day and a minimum of 5 days per week. Compliance will be tracked as all game data is automatically collected real-time on a central cloud server that preserves participant anonymity.<br>• The clinical site will be notified as to any participant number that failed to play EVO over a 24-hour period. To further protect anonymity, only the clinical site will then reach out to participants and parents to remind them to play (by email and/or text message).<br><br>*Afternoon* Exit – In-clinic (DAY 28)<br>• C-SSRS, since last visit, assessment will be conducted.<br>• The participants will play EVO for approximately 20 minutes in the clinic to re-establish their baseline performance. In addition a supplemental cognitive battery (CANTAB) and TOVA will be conducted along with the Parent-BRIEF survey.<br>• A safety assessment (AEs) and documentation of any reported concomitant medications or non-pharmacological treatment for ADHD or mental health.<br>• Questionnaires will be completed to collect participant feedback and parent feedback on the game experience.<br>• The iPad is returned.
Statistical Analysis	As a primary end-point, the study will be powered to detect a difference in EVO cognitive measures (interference cost) between neuro-typicals and ADHD participants.<br>• Power calculations (0.9, alpha = 0.05) are designed to detect a prevalence of impairment in an ADHD population of at least 40%, assuming a prevalence of impairment in the control group of 10% (assuming normal distribution for each group).<br>• Difference between groups will be determined using a two-sided t-test to compare specific groups with a threshold of $p < 0.05$, and group by variable interactions will be assessed with standard ANOVA.<br><br>The secondary objective/outcome to demonstrate that EVO is "playable" by the ADHD population is qualitative and no statistics will be applied.

Study Flow Chart

Period	Screening & Baseline	At-Home Training	Exit
Visit	1	-	2
Day	0	1 - 27	28
Informed consent and assent	X		
Demographic & contact information	X		
Parent BRIEF	X		X
Supplemental cognitive battery (CANTAB & TOVA)	X		X
MINI-Kid diagnosis	X		
ADHD-RS rating	X		
C-SSRS rating	X		X
EVO iPad baseline cognitive data	X		X
At-home EVO iPad cognitive training		X	
Feedback sessions and questionnaires	X		X
Safety assessments /AEs and concomitant medication / therapy	X		X
Measure behavioral play patterns		X	
Collect iPads			X

Appendix B (Example Informed Consent)

PARENT / CAREGIVER INFORMATION AND CONSENT FORM AND HIPAA AUTHORIZATION

**TITLE:**	A study to assess the feasibility of EVO, game-play, to engage participants with Attention Deficit Hyperactivity Disorder (ADHD) and to evaluate interference in children ages 8 to 12 year old with ADHD compared to age-matched controls
**PROTOCOL:**	Akili-001
**SPONSOR:**	Akili Interactive Labs, Inc.<br>500 Boylston Street, Suite 1600,<br>Boston, MA 02116
**PRINCIPAL INVESTIGATOR:**	Andrew J. Cutler, MD
**RESEARCH SITE ADDRESS(S):**	Florida Clinical Research Center, LLC<br>8043 Cooper Creek Blvd, Suite 107<br>Bradenton, FL 32401
**DAYTIME TELEPHONE NUMBER(S):**	941-747-7900
**24-HOUR CONTACT NUMBER(S):**	941-747-7900
**RESEARCH SITE ADDRESS(S):**	Florida Clinical Research Center, LLC<br>2300 Maitland Center Parkway, Suite 230<br>Maitland FL 32751
**DAYTIME TELEPHONE NUMBER(S):**	407-644-1165
**24-HOUR CONTACT NUMBER(S):**	407-644-1165

INTRODUCTION

The purpose of this research study is to assess the effectiveness of a game-play called EVO. When your child is a research participant, the study doctor (principal investigator) and the study staff will follow the rules of the research study (protocol).

Your child is being asked to voluntarily take part in a research study. You do not have to allow your child to participate if you do not want your child to participate. Before choosing to allow your child to be a part of this study, you need to read this Information and Consent Form.

This form tells you what will happen during the study and the risks and benefits for your child if you choose to allow him/her to take part in this study. It explains the other choices your child has besides taking part in this study. The form also explains you and your child's right to stop taking part in the study at any time. If you agree to allow your child to participate in this study, assent (agreement) will also be obtained from your child.

This consent form may contain words that you do not understand. Please ask the study doctor or study staff to explain any words or information that you do not clearly understand. Your questions should be answered clearly and to your satisfaction. Before you make a decision to allow your child to participate, we want you to understand the information in this form.

Sometimes, during a study, we may learn of new information which may make a difference in whether you want your child to continue to participate. If we learn of any information, we will let you and your child know as soon as possible.

SPONSOR

Akili Interactive Labs, Inc., is providing financial support and material for this study. Akili Interactive Labs, Inc., is paying the study doctor and study staff to do this study.

PURPOSE

The purpose of this study is to assess the feasibility of EVO, a game-play, to engage children with Attention Deficit Hyperactivity Disorder (ADHD) and to measure cognitive deficits compared to age-matched healthy children without ADHD. Approximately 80 children aged between 8 and 12 years will take part in this study, in 3 study centers in the U.S. 40 children will be children diagnosed with ADHD and not currently taking ADHD medications, and 40 children will be age-matched controls with no diagnosis of ADHD.

PROCEDURES AND PARTICIPANT RESPONSIBILITIES

If you decide to allow your child to participate in this study, he/she will be enrolled in the study for up to 29 days. Your child will have to come to the study center twice, accompanied by yourself. The study staff will tell you about the visit schedule and how long each visit will last. Your child will also play EVO at home for 30 minutes per day for a minimum of 5 days a week for 27 days while participating in the study**.** Note: It's very important that only the specified participant (child) plays EVO. No other family members or friends may play the game at any time during the 27 days.

Screening and Baseline visit procedures – In clinic:

- Demographic questions: at the first visit (Screening visit), you and your child will be asked to give identifying information about himself/herself, such as name, date of birth, and race.
- Diagnosis: you will be asked about your child's history of symptoms and behavior to confirm diagnosis of ADHD or not.
- Medication and safety questions: you will be asked about your child's medication status, a safety assessment and medication compliance will be recorded.
- EVO game-play and cognitive battery: your child will play EVO for approximately 20 minutes in the clinic and two cognitive batteries (CANTAB & TOVA) will be conducted with your child.
- Questionnaires: your child will be asked for feedback on their game experience, and you will be asked to complete the PARENT-BRIEF survey. You and your child will also be interviewed by the study doctor using a diagnostic measure (MINI-Kid) and a study staff-rated questionnaire (ADHD-RS-IV & C-SSRS).
- An iPad will be loaned to the participant for the duration of the study. EVO will be downloaded on to the iPad.

Training procedures – At home

- EVO game-play: your child will be asked to play EVO for approximately 30 minutes a day (with a maximum of 45 minutes) for a minimum of 5 days a week for 27 days.
- The study staff will be notified if your child fails to play EVO over a 24-hour period, and then they will contact you and/or your child by email or text message to remind your child to play EVO.

Exit visit procedures – In clinic

- You and your child will be interviewed by the study staff per a rated questionnaire (C-SSRS).
- EVO game-play and cognitive battery: your child will play EVO for approximately 20 minutes in the clinic and a two cognitive batteries (CANTAB & TOVA) will be conducted with your child.

- Questionnaires: you and your child will be asked for feedback on the game experience, and you will be asked to complete the PARENT-BRIEF survey.
- Medication compliance and safety questions: you will be asked some safety assessment questions and medication compliance will be recorded.
- The iPad will be returned.

RISKS

The risks of your child participating in this study are very small. The study involves completing some computerized tests and games and answering some questions. It is possible that your child could become frustrated by some of the tasks. Your child could become fatigued by the computer tests or game play. If your child becomes frustrated or fatigued, they may stop at any time. There are no other risks to taking part in this study of which we are aware.

POTENTIAL BENEFITS

Your child will not receive any direct benefit from participating in this study. No promise can be made concerning the study outcome, because results from a clinical research study cannot be predicted.

ALTERNATIVE METHODS/TREATMENTS

This study is voluntary. You may decide at any time to withdraw your child from participation in this study.

COSTS/PAYMENTS FOR PARTICIPATION IN RESEARCH

We do not expect there to be any costs to you or your child for participating in this study. Your child will not be paid to participate in this research study. You will be provided with $300 in total as compensation for time and travel to the study clinic ($50 at Screening/Baseline visit, $200-maximum for the at home game play & $50 for Exit visit). Compensation for at home play will be paid at the exit visit upon return of the IPad, if applicable. In the event a study participant fails to return the IPad their compensation for home play will be forfeited.

QUESTIONS ABOUT YOUR RIGHTS

By signing this form, you have not given up any of the legal rights that you or your child otherwise would have been entitled to as a participant in a research study.

VOLUNTARY PARTICIPATION AND WITHDRAWAL FROM THE RESEARCH

Your child's participation is voluntary. You may refuse to allow your child to participate for any reason at any time, without penalty or loss of benefits to which your child is otherwise entitled. If your child withdraws from the study you must return all study-related supplies to the study staff. You may withdraw your child from the study by contacting the study staff. If your child leaves the study before the regularly scheduled visit, you may be asked by the study staff to make a final visit for some end of study procedures. This is to make sure that there are no safety concerns to discuss with the study doctor and to maintain the integrity of the research study.

Your child's participation in this study may be stopped at any time by the study doctor or the sponsor without your consent because:

- the study doctor thinks it is necessary for your child's health or safety;
- your child or you have not followed study instructions;
- the sponsor has stopped the study;
- the IRB has decided to stop the study;
- administrative reasons require your child's withdrawal.

CONFIDENTIALITY

Your child's identity and your child's personal records will be kept confidential to the extent permitted by the applicable laws and/or regulations and will not be made publicly available. If results of this study are published or presented at a conference, your identity will not be revealed. Confidentiality will be maintained during and after your participation in this study.

Government agencies oversee research studies involving people. Your child's records may be reviewed by such agencies if your child takes party in this research study. These agencies include the Food and Drug Administration (FDA). By singing this consent form, you are allowing your child's records to be reviewed by these persons. It may be necessary to check parts of your child's record to be sure that the study data are correct and complete. No information other than what is needed for the study is recorded. Every effect is made to protect your child's privacy.

PHYSICIAN NOTIFICATION OPTION

Please place your initials beside your choice about notifying your child's regular doctor of your child's participation in this study.

_______ Yes, I want the study doctor to inform my child's regular doctor of his/her participation in this study.

_______ No, I do not want the study doctor to inform my child's regular doctor of his/her participation in this study.

_______ My child does not have a regular doctor.

STATEMENT OF CONSENT

I have been informed about this study's purpose, procedures, possible benefits and risks, and the use and disclosure of my child's health care information from this research. I have read and understood this consent form, and have been given the opportunity to ask any questions I may have. All my questions have been answered to my satisfaction. I freely give my consent for my child to participate in this research study. I authorize the use and disclosure of my child's health information to the parties listed in the authorization section of this consent for the purposes described above. By signing this consent form I have not waived any of the legal rights to which my child is otherwise entitled. You will be provided with a signed copy of this form.

CONSENT SIGNATURE

____	_______________________________
Printed name of Child	Child's Date of Birth (dd-MMM-yyyy)
____	_______________________________
Printed name of Parent/Legal Guardian	Relationship to Child
____	_______________________________________
Signature of Parent/Legal Guardian	Date (dd-MMM-yyyy)

PERSON OBTAINING CONSENT

I attest that the requirements for informed consent for this research project described in this form have been satisfied – that I have discussed the research project with the participant's parent or guardian and explained to him or her in nontechnical terms all of the information contained in this informed consent form, including any risks or adverse reactions that may reasonably be expected to occur. I certify that the information provided was given in a language that was understandable to the participant's parent or guardian. I further certify that I encouraged the parent or legal guardian to ask questions and that all questions asked were answered.

____
Printed name of Person Obtaining Consent

____ ____________________________________
Signature of Person Obtaining Consent Date (dd-MMM-yyyy)

____
Printed Name of Witness to the Consent Process
(*if applicable)

____ ______________________________
Signature of Witness to the Consent Process Date (dd-MMM-yyyy)
(*if applicable)

*Impartial Witness: If the participant's parent/legal guardian cannot read, the signature of an Impartial Witness is needed. An impartial witness is a person who independent of the trial, who cannot be unfairly influenced by people involved with the trial, who attends the informed consent process, and who reads the informed consent form and any other written information supplied to the participant's parent/legal guardian.

HIPAA AUTHORIZATION TO RELEASE INFORMATION FOR RESEARCH

The United States government has issued a privacy rule to protect the privacy rights of participants. This rule was issues under a law called the Health Insurance Portability and Accountability Act of 1996 (HIPAA). The Privacy Rule is designed to protect the confidentiality of your child's personal health information. If you agree for your child to be in this study, privacy laws require you to sign this Authorization that describes your rights and explains how your child's Protected Health Information (PHI) will be used and disclosed for this research study.

By signing this informed consent/HIPAA Authorization, you will be authorizing the principal investigator, his/her research staff, the sponsor, the Institutional Review Board (IRB), the U.S. Food and Drug Administration (FDA), and other regulatory agencies to use and disclose your child's PHI for the purposes described below.

This Authorization does not have an expiration date. This means the researchers and others associated with this study may use and disclose your child's PHI for as long as necessary to complete the study. If you do not withdraw this authorization in writing, it will remain in effect indefinitely. You are free to withdraw your Authorization regarding the use and disclosure of your child's PHI at any time. After any revocation, your child's PHI will no longer be used or disclosed in the study, except to the extent that the law allows the study staff to continue using your child's information (e.g., information necessary to maintain the integrity or reliability of the research). If you wish to revoke your Authorization for the research use or disclosure of your child's PHI in this study, you must write to:

Andrew J. Cutler, MD
Florida Clinical Research Center, LLC
8043 Cooper Creek Blvd, Suite 107
Bradenton, FL 32401

If you agree for your child to take part in this research study, your child's health information related to this study may be used or disclosed in connection with this research study, including, but not limited to:

- Demographic information
- Health and medication information
- Questionnaires and cognitive performance information

Florida Clinical Research Center, LLC is required by law to protect your child's PHI. By signing this document, you authorize Florida Clinical Research Center, LLC to use and/or disclose (release) your child's PHI for this research study. You acknowledge that you have received a copy of this form.

______________________________	______________________________
Printed name of Child	Child's Date of Birth (dd-MMM-yyyy)
______________________________	______________________________
Printed name of Parent/Legal Guardian	Relationship to Child
______________________________	______________________________
Signature of Parent/Legal Guardian	Date (dd-MMM-yyyy)
______________________________	
Printed name of Person Obtaining Authorization	
______________________________	______________________________
Signature of Person Obtaining Authorization	Date (dd-MMM-yyyy)

Appendix C (Example Child Assent)

CHILD ASSENT FORM

**TITLE:**	A study to assess the feasibility of EVO, game-play, to engage participants with Attention Deficit Hyperactivity Disorder (ADHD) and to evaluate interference in children ages 8 to 12 year old with ADHD compared to age-matched controls
**PROTOCOL:**	Akili-001
**SPONSOR:**	Akili Interactive Labs, Inc.<br>500 Boylston Street, Suite 1600,<br>Boston, MA 02116
**PRINCIPAL INVESTIGATOR:**	Andrew J. Cutler, MD
**RESEARCH SITE ADDRESS(S):**	Florida Clinical Research Center, LLC<br>8043 Cooper Creek Blvd, Suite 107<br>Bradenton, FL 32401
**DAYTIME TELEPHONE NUMBER(S):**	941-747-7900
**24-HOUR CONTACT NUMBER(S):**	941-747-7900
**RESEARCH SITE ADDRESS(S):**	Florida Clinical Research Center, LLC<br>2300 Maitland Center Parkway, Suite 230<br>Maitland FL 32751
**DAYTIME TELEPHONE NUMBER(S):**	407-644-1165
**24-HOUR CONTACT NUMBER(S):**	407-644-1165

INTRODUCTION

You are being asked to be in a research study. You can choose to be in the study or not to be in the study. You don't have to be in the study if you don't want to. If you decide not to be in the study, no one will be angry with you and you will not get into trouble. We will tell you about the study and what we will ask you to do if you want to be in the study. You can ask questions at any time. You can ask the study doctor or staff questions before you make up your mind. You can also talk to your mom or dad or anyone else to ask any questions you may have.

PURPOSE

In this study we want to find out if EVO, a game you play on an IPad, will measure differences in abilities in children with Attention Deficit Hyperactivity Disorder (ADHD) and children without ADHD.

PROCEDURES AND RESPONSIBILITIES

If you decide to take part in the study, you will be in the study for up to 29 days. You will have to come to the study center twice, with your parent / caregiver.

Here are some things the study staff will do at your two study visits. The study staff can tell you what will happen at each visit.

- Ask you and your parent/legal guardian questions about yourself, such as your name and birth date.
- Ask you and your parent/legal guardian about your behavior, your health and about any medications you may be taking.
- You will play EVO game-play on an IPad for 20 minutes, and also complete a cognitive test with study staff.
- You will be asked to tell us what you thought of the EVO game, and your parent/legal guardian will fill out a questionnaire about your behavior at home.

You will also play EVO at home for approximately 30 minutes a day (but up to 45 minutes) for 5 days a week for 27 days while you are in the study. The study staff will contact you to remind you to play EVO if you haven't played EVO for 24 hours. Note: It's very important that <u>only</u> you play EVO. <u>No</u> other family members or friends may play the game at any time during the 27 days.

RISKS & POTENTIAL BENEFITS

This study involves asking some questions and completing some computer tests and a game on an IPad. You could become frustrated with some of the tasks, or get tired. You can stop at any time if this happens. There are no other risks to taking part in this study. There is no direct benefit from participating in this study.

VOLUNTARY PARTICIPATION AND WITHDRAWAL FROM RESEARCH

It is up to you if you take part in this study. Even if you do agree to take part, you may stop at any time for any reason. If you don't want to be in the study or if you start the study and later decide to not be

in this study that is fine. If you change your mind later, tell your parent/ legal guardian and the study staff.

Your parent/ legal guardian may also choose to have you stop the study at any time for any reason. The study doctor or the company that runs this study may stop this study at any time. If this happens, you will no longer be in this study.

You can ask questions about the study at any time. You can call the study doctor any time. The study doctor's phone number is one the first page.

PRIVACY

Other people will not know if you are in the study. We will put things we learn about you together with things we learn about other children. No one can tell what things came from you. When we tell other people about our research, we will not use your name, so no one can tell who we are talking about.

The information we take about you during this study will be kept locked up. No one will know it except for the people doing the research. We will not tell anyone any of your information unless:

- We found out you were in serious danger, or
- We found out someone else was in serious danger.

STATEMENT OF ASSENT

I have read or have had read the assent form to me by someone I trust. I agree to be in this research study. I know that my parent/legal guardian must also agree to let me be in this study.

I have asked questions about the study. My questions have been answered so far. I can continue to ask any questions at any time during the study. I know that being in the research study is up to me. I will get a copy of this form after I sign it.

Please sign below if you want to be in this study, but remember: You do not have to be in this study if you don't want to. You can stop at any time and no one will be upset with you.

ASSENT SIGNATURE

____ ________________________

Printed name of Child Date of Birth (dd-MMM-yyyy)

____ ______________________________

Signature of Participant Date (dd-MMM-yyyy)

I certify that the participant had enough time to consider this information, had an opportunity to ask questions, and voluntarily agreed to be in this study.

____

Printed name of Person Obtaining Assent

____ ______________________________

Signature of Person Obtaining Assent Date (dd-MMM-yyyy)

Appendix D (Participant Questionnaire)

**EVO Gameplay**<br><br>**Completed by Study Staff**<br><br>**DAY 0**<br><br>**and**<br><br>**DAY 28**<br><br>**(circle above)**<br><br>**Participant #:______**	In terms of enjoyment how would you rate playing EVO?<br>**1 2 3 4 5 6 7 8 9 10 (circle)**<br>**Boring OK Fun**<br><br>How challenging was EVO to play?<br>**1 2 3 4 5 6 7 8 9 10 (circle)**<br>**Easy Moderate Hard**<br><br>Was there anything you really liked about playing EVO?<br><br>Was there anything you really disliked about playing EVO?<br>(If you stopped playing EVO please explain why)<br><br>What would make you want to play even more?<br><br>Did you feel like your performance improved?<br><br>What individual skills did you get better at?<br><br>What did you hope you got even better at?<br><br>Do you felt like you improved in anything in the real world?<br>**YES / NO (circle)**<br>What, specifically?<br><br>Did you like trying to get stars? **YES / NO (circle)**<br>Was it easy or hard? **EASY / HARD (circle)**

**EVO Gameplay**<br><br>**Completed by Study Staff**<br><br>**DAY 0**<br><br>**and**<br><br>**DAY 28**<br><br>**(circle above)**<br><br>**Participant #:______**	Would you play EVO instead of your regular games?<br>**NO / MAYBE / YES (circle)**<br><br>How often do you play video games?<br>**NEVER / ONCE A MONTH / ONCE A WEEK / EVERY DAY (circle)**<br><br>How many hours a week do you play?<br>**Less than 1 HOUR / 1–5 HOURS / 5–10 HOURS / Greater than 10 HOURS (circle)**<br><br>What types of games do you like to play?<br>**CONSOLE / PHONE / TABLET / COMPUTER / OTHER (describe) (circle)**<br><br>What are some of your favorite games?

Appendix E (Executive Summary)

AKILI
interactive labs

Akili Interactive Labs is creating the first clinically-validated medical products delivered in an immersive "video game" format. We believe that in the near future, we will see a clinician prescribe a mobile therapeutic or deploy remote diagnostics that exist totally as mobile apps. Akili's overall strategy is to become known as the leading company in this new emerging field of medicine, by combining the best neuroscience with top-tier engagement. Akili has received recent recognition for this pioneering approach, including highlights in the Wall Street Journal and Nature.

Background

Akili's first product is based on the work of Prof. Adam Gazzaley from UCSF, who has identified a way to measure and improve the ability to process cognitive interference (essentially, how a patient responds to interruptions and distractions). Interference susceptibility is recognized as a limiting factor across global cognitive function and is known to be fragile in multiple diseases. The basic scientific mechanisms underlying our first game have been tested in an N = 203 study to validate the ability to measure interference processing ability from 8 to 80 years of age, and in a controlled N = 60 study to validate the ability to improve this ability. The cognitive benefits of training appear to generalize beyond the game environment, as subjects showed improvements on independent executive function tests of sustained attention, impulsivity, and working memory – and robust physiological brain changes - compared with controls.

There are multiple important clinical populations that have, as hallmarks of the disease, impairments in executive function. Akili is targeting two of these, ADHD and autism, as its first high-priority clinical markets. Akili is also interested to explore multiple other conditions (e.g., depression and age-related disorders) that would benefit from improvements or ongoing tracking of executive function.

Product Development and Studies

Akili has developed an adaptive cognitive gaming engine that automatically customizes difficulty to the subject. This platform enables two main product types: personalized therapeutic games, and a high-resolution tool to remotely distinguish and track disease progress. Importantly, our game environment is a key to get repeated, remote engagement by the patient. Our first clinical product has been through multiple rounds of testing and refinement in both norms and clinical populations. Akili has launched (or is in the process of initiating) a total of 7 pilot studies in our highest-priority clinical populations, including multiple studies with leading academics and clinics. Early data from our most recent game has shown promising ability to separate populations based on their gameplay signatures.

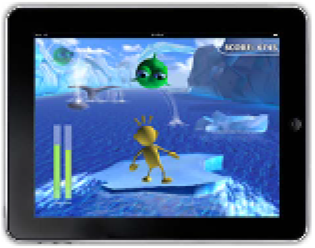

Akili Prototype Screenshot

Company Background

Akili was founded by PureTech Ventures, a leading health-focused venture creation company. Akili's game design team includes Matt Omernick, the former Executive Art Director at LucasArts and Cinematics Director at Microsoft, and Adam Piper who previously led the rapid prototyping group at LucasArts. Akili's co-founding Chief Science Advisor Prof. Adam Gazzaley of UCSF has led the research into the mechanisms behind Akili's first game. His work has been published in top tier academic journals and featured in the popular press including CNN and the New York Times. Other co-founders include U. Rochester's Daphne Bavelier, who has published pioneering research on the active components of action video games, and prominent technology thought-leaders (including video game design veteran Noah Falstein and Second Life Founder and former RealNetworks CTO Philip Rosedale).

500 Boylston Street, Suite 1600, Boston, Massachusetts
tel: 617-456-0597
www.akiliinteractive.com

Appendix F (Parent BRIEF)

BRIEF

Behavior Rating Inventory of Executive Function™

PARENT FORM

Gerard A. Gioia, PhD, Peter K. Isquith, PhD, Steven C. Guy, PhD, and Lauren Kenworthy, PhD

Instructions

On the following pages is a list of statements that describe children. We would like to know if your child has had problems with these behaviors over the past 6 months. Please answer all the items the best that you can. Please DO NOT SKIP ANY ITEMS. Think about your child as you read each statement and circle your response:

N	if the behavior is	**Never** a problem
S	if the behavior is	**Sometimes** a problem
O	if the behavior is	**Often** a problem

For example, if your child **never** has trouble completing homework on time, you would circle N for this item:

Has trouble completing homework on time (N) S O

If you make a mistake or want to change your answer, DO NOT ERASE. Draw an "X" through the answer you want to change, and then circle the correct answer:

Has trouble completing homework on time (X) (S) O

Before you begin answering the items, please fill in your child's name, gender, grade, age, birth date, your name, your relationship to the child, and today's date in the spaces provided at the top of the next page.

Copyright © 1996, 1998, 2000 by Psychological Assessment Resources, Inc. All rights reserved. May not be reproduced in whole or in part in any form or by any means without written permission of Psychological Assessment Resources, Inc. This form is printed in green ink on white paper. Any other version is unauthorized.

9 8 7 6 Reorder #RO-4467 Printed in the U.S.A

Child's Name ______________ Gender _____ Grade _____ Age _____ Birth Date___/___/___

Your Name ______________ Relationship to Child______________ Today's Date___/___/___

N = Never S = Sometimes O = Often

1.	Overreacts to small problems	N	S	O
2.	When given three things to do, remembers only the first or last	N	S	O
3.	Is not a self-starter	N	S	O
4.	Leaves playroom a mess	N	S	O
5.	Resists or has trouble accepting a different way to solve a problem with schoolwork, friends, chores, etc.	N	S	O
6.	Becomes upset with new situations	N	S	O
7.	Has explosive, angry outbursts	N	S	O
8.	Tries the same approach to a problem over and over even when it does not work	N	S	O
9.	Has a short attention span	N	S	O
10.	Needs to be told to begin a task even when willing	N	S	O
11.	Does not bring home homework, assignment sheets, materials, etc.	N	S	O
12.	Acts upset by a change in plans	N	S	O
13.	Is disturbed by change of teacher or class	N	S	O
14.	Does not check work for mistakes	N	S	O
15.	Has good ideas but cannot get them on paper	N	S	O
16.	Has trouble coming up with ideas for what to do in play or free time	N	S	O
17.	Has trouble concentrating on chores, schoolwork, etc.	N	S	O
18.	Does not connect doing tonight's homework with grades	N	S	O
19.	Is easily distracted by noises, activity, sights, etc.	N	S	O
20.	Becomes tearful easily	N	S	O
21.	Makes careless errors	N	S	O
22.	Forgets to hand in homework, even when completed	N	S	O
23.	Resists change of routine, foods, places, etc.	N	S	O
24.	Has trouble with chores or tasks that have more than one step	N	S	O
25.	Has outbursts for little reason	N	S	O
26.	Mood changes frequently	N	S	O
27.	Needs help from an adult to stay on task	N	S	O
28.	Gets caught up in details and misses the big picture	N	S	O
29.	Keeps room messy	N	S	O
30.	Has trouble getting used to new situations (classes, groups, friends)	N	S	O
31.	Has poor handwriting	N	S	O
32.	Forgets what he/she was doing	N	S	O
33.	When sent to get something, forgets what he/she is supposed to get	N	S	O
34.	Is unaware of how his/her behavior affects or bothers others	N	S	O
35.	Has good ideas but does not get job done (lacks follow-through)	N	S	O
36.	Becomes overwhelmed by large assignments	N	S	O
37.	Has trouble finishing tasks (chores, homework)	N	S	O
38.	Acts wilder or sillier than others in groups (birthday parties, recess)	N	S	O
39.	Thinks too much about the same topic	N	S	O
40.	Underestimates time needed to finish tasks	N	S	O
41.	Interrupts others	N	S	O
42.	Does not notice when his/her behavior causes negative reactions	N	S	O
43.	Gets out of seat at the wrong times	N	S	O
44.	Gets out of control more than friends	N	S	O

N = Never S = Sometimes O = Often

45. Reacts more strongly to situations than other children	N	S	O
46. Starts assignments or chores at the last minute	N	S	O
47. Has trouble getting started on homework or chores	N	S	O
48. Has trouble organizing activities with friends	N	S	O
49. Blurts things out	N	S	O
50. Mood is easily influenced by the situation	N	S	O
51. Does not plan ahead for school assignments	N	S	O
52. Has poor understanding of own strengths and weaknesses	N	S	O
53. Written work is poorly organized	N	S	O
54. Acts too wild or "out of control"	N	S	O
55. Has trouble putting the brakes on his/her actions	N	S	O
56. Gets in trouble if not supervised by an adult	N	S	O
57. Has trouble remembering things, even for a few minutes	N	S	O
58. Has trouble carrying out the actions needed to reach goals (saving money for special item, studying to get a good grade)	N	S	O
59. Becomes too silly	N	S	O
60. Work is sloppy	N	S	O
61. Does not take initiative	N	S	O
62. Angry or tearful outbursts are intense but end suddenly	N	S	O
63. Does not realize that certain actions bother others	N	S	O
64. Small events trigger big reactions	N	S	O
65. Talks at the wrong time	N	S	O
66. Complains there is nothing to do	N	S	O
67. Cannot find things in room or school desk	N	S	O
68. Leaves a trail of belongings wherever he/she goes	N	S	O
69. Leaves messes that others have to clean up	N	S	O
70. Becomes upset too easily	N	S	O
71. Lies around the house a lot ("couch potato")	N	S	O
72. Has a messy closet	N	S	O
73. Has trouble waiting for turn	N	S	O
74. Loses lunch box, lunch money, permission slips, homework, etc.	N	S	O
75. Cannot find clothes, glasses, shoes, toys, books, pencils, etc.	N	S	O
76. Tests poorly even when knows correct answers	N	S	O
77. Does not finish long-term projects	N	S	O
78. Has to be closely supervised	N	S	O
79. Does not think before doing	N	S	O
80. Has trouble moving from one activity to another	N	S	O
81. Is fidgety	N	S	O
82. Is impulsive	N	S	O
83. Cannot stay on the same topic when talking	N	S	O
84. Gets stuck on one topic or activity	N	S	O
85. Says the same things over and over	N	S	O
86. Has trouble getting through morning routine in getting ready for school	N	S	O

Appendix G (CANTAB)

cambridge cognition

Cambridge Cognition Ltd develops and markets CANTAB®. Based in Cambridge, UK and Cambridge, Massachusetts, Cambridge Cognition supplies its products and services to the pharmaceutical industry and academic researchers across the globe.

CANTAB® | ADHD™

Attention Deficit Hyperactivity Disorder (ADHD) in both children and adults is characterised at the neurobehavioural level by inattention and impulsivity. These manifestations are underpinned by cognitive deficits in sustained attention, working memory and response inhibition. The **CANTAB** touch screen allows these neurobehavioural manifestations to be assessed in a quick and standard fashion, using a single instrument in a straightforward and user-friendly manner.

Completed in around 30 minutes, the **CANTAB ADHD** battery consists of four tests designed to assess executive functioning, working memory, higher-order attention (sustained attention over time) and response impulsivity. The battery is a reflection of our experience in assessing these cognitive domains in adult and paediatric ADHD, in case-control and clinical trial research, alongside the broader use of these measures in a range of CNS domains.

- **SENSITIVE** to the essential features of ADHD, validated through careful academic case-control studies.
- **THOROUGH** repeatable assessment, including measurements of attention, impulsivity, working memory and executive function.
- **RAPID** deployment to sites for use.
- **OBJECTIVE** testing, minimising rater and site variability.
- **MEASURES** are extremely responsive to detecting drug effects over short time periods, enabling you to run smaller, faster trials.
- **TRANSLATIONAL** platform for drug development with validation through preclinical and neuroimaging variants.
- Gives you an **EFFICIENT** means of showing proof of concept.

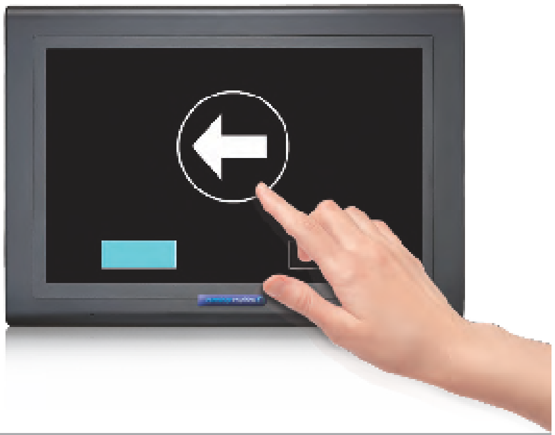

SST Test →

www.cambridgecognition.com

Appendix H (ADHD-RS)

Subject Number: ___ ___ ___-___ ___ __ Visit _____

ADHD-RS-IV: Clinician Version (ADHD-RS)

Date of Assessment: ____/_______/______ Rater's Initials: ___ / ___ / ___
DD / MMM / YYYY

**Circle the number that *best describes* this subject's behaviour since the last visit.**	Never or rarely	Sometimes	Often	Very Often
1. Fails to give close attention to details or makes careless mistakes in schoolwork	0	1	2	3
2. Fidgets with hands or feet or squirms in seat	0	1	2	3
3. Has difficulty sustaining attention in tasks or play activities	0	1	2	3
4. Leaves seat in classroom or in other situations in which remaining seating is expected	0	1	2	3
5. Does not seem to listen when spoken to directly	0	1	2	3
6. Runs about or climbs excessively in situations in which it is inappropriate	0	1	2	3
7. Does not follow through on instructions and fails to finish work	0	1	2	3
8. Has difficulty playing or engaging in leisure activities quietly	0	1	2	3
9. Has difficulty organizing tasks and activities	0	1	2	3
10. Is "on the go" or acts as if "driven by a motor"	0	1	2	3
11. Avoids tasks (e.g., schoolwork, homework) that require sustained mental effort	0	1	2	3
12. Talks excessively	0	1	2	3
13. Loses things necessary for tasks or activities	0	1	2	3
14. Blurts out answers before questions have been completed	0	1	2	3
15. Is easily distracted	0	1	2	3
16. Has difficulty awaiting turn	0	1	2	3
17. Is forgetful in daily activities	0	1	2	3
18. Interrupts or intrudes on others	0	1	2	3

From **ADHD Rating Scale-IV: Checklists, Norms and Clinical Interpretation**. Reprinted with permission of The Guilford Press: New York. © 1998 George J. DuPaul, Thomas J. Power, Arthur A. Anastopoulos and Robert Reid.

Signature (of clinician completing assessment): ______________________ Date:__________

Appendix I (MINI – Kid)

M.I.N.I. KID

MINI INTERNATIONAL NEUROPSYCHIATRIC INTERVIEW
For Children and Adolescents

English Version 6.0

DSM-IV

USA: **D. Sheehan, D. Shytle, K. Milo, J. Janavs**
University of South Florida College of Medicine - Tampa, USA

FRANCE: **Y. Lecrubier**
Centre Hôpitalier Sainte-Anne - Paris, France

© Copyright 1994-2010 Sheehan DV and Lecrubier Y.

All rights reserved. No part of this document may be reproduced or transmitted in any form, or by any means, electronic or mechanical, including photocopying, or by any information storage or retrieval system, without permission in writing from Dr. Sheehan. Researchers and study staffs working in nonprofit or publicly owned settings (including universities, nonprofit hospitals, and government institutions) may make paper copies of a M.I.N.I. KID instrument for their own clinical and research use.

DISCLAIMER

Our aim is to assist in the assessment and tracking of patients with greater efficiency and accuracy. Before action is taken on any data collected and processed by this program, it should be reviewed and interpreted by a licensed study staff. This program is not designed or intended to be used in the place of a full medical and psychiatric evaluation by a qualified licensed physician – psychiatrist. It is intended only as a tool to facilitate accurate data collection and processing of symptoms elicited by trained personnel.

Patient Name:		***Patient Number:***	
Date of Birth:		***Time Interview Began:***	
Interviewer's Name:		***Time Interview Ended:***	
Date of Interview:		***Total Time:***	

	MODULES	TIME FRAME	MEETS CRITERIA	DSM-IV	ICD-10	
A	MAJOR DEPRESSIVE EPISODE	Current (Past 2 weeks)	⑦			
		Past	⑦			
		Recurrent	⑦			
	MAJOR DEPRESSIVE DISORDER	Current (Past 2 weeks)	⑦	296.20-296.26 Single	F32.x	⑦
		Past	⑦	296.20-296.26 Single	F33.x	⑦
		Recurrent	⑦	296.30-296.36 Recurrent	F33.x	⑦
B	SUICIDALITY	Current (Past Month)	⑦	N/A	N/A	
		⑦ Low ⑦ Moderate ⑦ High				
C	DYSTHYMIA	Current (Past 1 year)	⑦	300.4	F34.1	⑦
D	MANIC EPISODE	Current	⑦			
		Past	⑦			
	HYPOMANIC EPISODE	Current	⑦			
		Past	⑦	⑦ Not Explored		
	BIPOLAR I DISORDER	Current	⑦	296.0x-296.6x	F30.x- F31.9	⑦
		Past	⑦	296.0x-296.6x	F30.x- F31.9	⑦
	BIPOLAR II DISORDER	Current	⑦	296.89	F31.8	⑦
		Past	⑦	296.89	F31.8	⑦
	BIPOLAR DISORDER NOS	Current	⑦	296.80	F31.9	⑦
		Past	⑦	296.80	F31.9	⑦
E	PANIC DISORDER	Current (Past Month)	⑦	300.01/300.21	F40.01-F41.0	⑦
		Lifetime	⑦	300.01/300.21	F40.01-	
F41.0		⑦				
F	AGORAPHOBIA	Current	⑦	300.22	F40.00	⑦
G	SEPARATION ANXIETY DISORDER	Current (Past Month)	⑦	309.21	F93.0	⑦
H	SOCIAL PHOBIA (Social Anxiety Disorder)	Current (Past Month)				
		Generalized	⑦	300.23	F40.1	⑦
		Non-Generalized	⑦	300.23	F40.1	⑦
I	SPECIFIC PHOBIA	Current (Past Month)	⑦	300.29	N/A	⑦
J	OBSESSIVE COMPULSIVE DISORDER	Current (Past Month)	⑦	300.3	F42.8	⑦
K	POST TRAUMATIC STRESS DISORDER	Current (Past Month)	⑦	309.81	F43.1	⑦
L	ALCOHOL DEPENDENCE	Past 12 Months	⑦	303.9	F10.2x	⑦
L	ALCOHOL ABUSE	Past 12 Months	⑦	305.00	F10.1	⑦
M	SUBSTANCE DEPENDENCE (Non-alcohol)	Past 12 Months	⑦	304.00-.90/305.20-.90	F11.2X-F19.2X	⑦
M	SUBSTANCE ABUSE (Non-alcohol)	Past 12 Months	⑦	304.00-.90/305.20-.90	F11.1-F19.1	⑦
N	TOURETTE'S DISORDER	Current	⑦	307.23	F95.2	⑦

	MOTOR TIC DISORDER		Current	⑦	307.22	F95.1	⑦
	VOCAL TIC DISORDER		Current	⑦	307.22	F95.1	⑦
	TRANSIENT TIC DISORDER		Current	⑦	307.21	F95.0	⑦
O	ADHD	COMBINED	Past 6 Months	⑦	314.01	F90.0	⑦
	ADHD	INATTENTIVE	Past 6 Months	⑦	314.00	F98.8	⑦
	ADHD	HYPERACTIVE/IMPULSIVE	Past 6 Months	⑦	314.01	F90.0	⑦
P	CONDUCT DISORDER		Past 12 Months	⑦	312.8	F91.x	⑦
Q	OPPOSITIONAL DEFIANT DISORDER		Past 6 Months	⑦	313.81	F91.3	⑦
R	PSYCHOTIC DISORDERS		Lifetime	⑦	295.10-295.90/297.1/	F20.xx-F29	⑦
			Current	⑦	297.3/293.81/293.82/<br>293.89/298.8/298.9	F20.xx-F29	⑦
	MOOD DISORDER WITH PSYCHOTIC FEATURES		Lifetime	⑦	296.24/296.04-296.94	F30.2/F31.2/F31.5/<br>F31.65/F32.3/F33.3	⑦
			Current	⑦	296.24/296.04-296.94		
	F30.2/F31.2/F31.5/<br>F31.65/F32.3/F33.3		⑦				
S	ANOREXIA NERVOSA		Current (Past 3 Months)	⑦	307.1	F50.0	⑦
T	BULIMIA NERVOSA		Current (Past 3 Months)	⑦	307.51	F50.2	⑦
	ANOREXIA NERVOSA, BINGE EATING/PURGING TYPE		Current	⑦	307.1	F50.0	⑦
U	GENERALIZED ANXIETY DISORDER		Current (Past 6 Months)	⑦	300.02	F41.1	⑦
V	ADJUSTMENT DISORDERS		Current	⑦	309.24/309.28<br>309.3/309.4	F43.xx	⑦
W	MEDICAL, ORGANIC, DRUG CAUSE RULED OUT			⑦ No	⑦ Yes	⑦ Uncertain	
X	PERVASIVE DEVELOPMENTAL DISORDER		Current	⑦	299.00/299.10/299.80	F84.0/.2/.3/.5/.9	⑦

PRIMARY DISORDER

IDENTIFY THE PRIMARY DIAGNOSIS BY CHECKING THE APPROPRIATE CHECK BOX.

Which problem troubles him/her the most or dominates the others or came first in the natural history?

DISCLAIMER

Our aim is to assist in the assessment and tracking of patients with greater efficiency and accuracy. Before action is taken on any data collected and processed by this program, it should be reviewed and interpreted by a licensed study staff.

This program is not designed or intended to be used in the place of a full medical and psychiatric evaluation by a qualified licensed physician – psychiatrist. It is intended only as a tool to facilitate accurate data collection and processing of symptoms elicited by trained personnel

Appendix J (EVO Game Play Instructions for the Parent/Participant)

EVO Game Play At-Home Instructions

Now that you have returned home with the EVO video application loaded on an iPad the following instructions will help you over the next 27 days of at home game play.

1) If at any time during the 27 day at-home play session you have any questions or concerns please contact the Investigator identified on your copy of the signed informed consent form.

2) Prior to playing EVO it is important to <u>one-time</u> configure the iPad to connect to your home's wireless router. Connecting to your router is easy and allows the child's play data to be analyzed real-time. **To do this:**

- Turn on the iPad and **"slide"** the tab at the bottom to unlock the device
- Tap on the **"Setting's"** icon
- From the left-hand side of the screen tap on **"Wi-Fi"**
- Make sure the Wi-Fi setting is selected as **"ON"**
- Under **"Choose a Network"** tap on the network corresponding to your home wireless router
- When prompted, **enter the password** to your wireless router and tap on **"Join"** Remember this step only needs to be done once. If you don't have a wireless router at home, please connect the iPad to the internet periodically at an alternate point of connectivity.

3) During the next 27 days the child should play EVO at least <u>5 days a week</u> for typically <u>30 minutes a day</u>. This is accomplished by playing 7 consecutive runs of approximately 4 minutes each. Sometimes the game may ask the child to repeat an internal diagnostic which may take an additional 15 minutes.

Note: The game will automatically "lock" after 7 runs in any given day, and that the child will not be able to play until the following day.

Note: If the child becomes fatigued or frustrated with the game after 30 minutes of play, in any given day, he or she can turn the game off and it will automatically pick up where it left off during the next play session.

To play EVO:

- Tap on the **"EVO"** icon
  (*When you were at the lab, the child was assigned a subject identifier number which should be displayed on the middle of the right side of the screen. If the number is <u>**not**</u> there contact the investigator for instructions.)*
- Tap the **"EVOLVE"** button.
- Tap on the world that is highlighted. This could be "**Frozen Solid," "Melty Mania," "Deep Freeze," or "Snow Blind**" depending on the child's progress in the game.

- Follow the instructions described by the animated character in the laboratory coat.

4) Note: It's very important that only the specified participant (child) plays EVO. No other family members or friends may play the game at any time during the 27 days!

5) Note: The iPad provided in this study is locked from internet access and from uploading other applications. It is important to return this iPad when you return for the in-clinic exit visit.

Appendix K (EVO Game Play Instructions for the Investigator)

EVO Game Instructions for the Investigator

Day 0 and Day 28 Instructions

Making sure the iPad is connected to the clinic's router

Make sure the iPad is configured to connect to the wireless router in your clinic. EVO needs to upload data real time to the Akili server during the participant's game play. **To connect to the router:**

- Turn on the iPad and **"slide"** the tab at the bottom to unlock the device
- Tap on the **"Setting's"** icon
- From the left-hand side of the screen tap on **"Wi-Fi"**
- Make sure the Wi-Fi setting is selected as **"ON"**
- Under **"Choose a Network"** tap on the network corresponding to your clinic's wireless router
- When prompted, **enter the password** to your wireless router and tap on **"Join"**.

Day 0 Only Instructions

Resetting EVO & Adding a New Participant for In-clinic Session

1) EVO needs to be reset to clear out any data captured corresponding to previous participants. **To erase EVO's memory:**

- Tap on the **"EVO"** icon
- Tap on **"NEW USER"**
- In the Subject Number field enter **"321Admin" (case sensitive)**
- Tap on "**ENABLE/DISABLE**" until Multi-Users is Disabled
- Tap on **"ERASE ALL" and "YES"**
- Tap on **"BACK"**

2) Adding a new participant. **To enter a new participant's information:**

- Tap on **"NEW USER"**
- In the Subject Number field enter the 3-digit number assigned to this participant **"###-P"** (The "P" denotes that this is the first in-clinic practice and assessment sessions)
- In the Study Number field enter **"Akili-001"**
- Tap on **"Login"**

Playing EVO for the First Time

1) To play EVO**:**

- Have your participant tap on the **"EVO"** icon.
  (*Verify the correct participant identifier number is displayed on the middle of the right side of the screen. Followed by "-P" denoting the first in-clinic practice and assessment*

*sessions. If the number is **not** correct repeat the resetting and adding a new participant steps.*

- Have participant tap the **"EVOLVE"** button.
- Have participant tap the **"START"** button to execute EVO.
- Have participant follow the instructions described by the animated character in the laboratory coat.

2) Make sure the instructions are understood and have the participant perform:

- One EVO practice session
- One EVO diagnostic/assessment session
- One EVO training session

Setting up EVO for Take Home

1) Prior to taking the iPad home, EVO needs to be reset one more time to clear out the in-clinic practice and assessment sessions and to ready it for home play. **To erase EVO's memory:**

- Tap on the **"EVO"** icon
- Tap on **"NEW USER"**
- In the Subject Number field enter **"321Admin" (case sensitive)**
- Tap on "**ENABLE/DISABLE**" until Multi-Users is Disabled
- Tap on **"ERASE ALL" and "YES"**
- Tap on **"BACK"**

2) Re-enter the participant number without the "-P" extension. **To re-enter the participant's information:**

- Tap on **"NEW USER"**
- In the Subject Number field enter the 3-digit number assigned to this participant **"###"**
- In the Study Number field enter **"Akili-001"**
- Tap on **"Login"**

Day 28 Only Instructions

Playing EVO for the Last Time

1) To play EVO:

- Have your participant tap on the **"EVO"** icon
- Have participant tap the **"EVOLVE"** button.
- Have participant tap the **"START"** button to execute EVO.
- Have participant play EVO for approximately 20 minutes.

2) Collect the iPad

Appendix L (Columbia-Suicide Severity Rating Scale)

COLUMBIA-SUICIDE SEVERITY RATING SCALE (C-SSRS)

Lifetime/Recent Version

Version 1/14/09

Posner, K.; Brent, D.; Lucas, C.; Gould, M.; Stanley, B.; Brown, G.; Fisher, P.; Zelazny, J.; Burke, A.; Oquendo, M.; Mann, J.

Disclaimer:

This scale is intended to be used by individuals who have received training in its administration. The questions contained in the Columbia-Suicide Severity Rating Scale are suggested probes. Ultimately, the determination of the presence of suicidal ideation or behavior depends on the judgment of the individual administering the scale.

Definitions of behavioral suicidal events in this scale are based on those used in **The Columbia Suicide History Form**, *developed by John Mann, MD and Maria Oquendo, MD, Conte Center for the Neuroscience of Mental Disorders (CCNMD), New York State Psychiatric Institute, 1051 Riverside Drive, New York, NY, 10032. (Oquendo M. A., Halberstam B. & Mann J. J., Risk factors for suicidal behavior: utility and limitations of research instruments. In M.B. First [Ed.]* Standardized Evaluation in Clinical Practice, *pp. 103 -130, 2003.)*

For reprints of the C-SSRS contact Kelly Posner, Ph.D., New York State Psychiatric Institute, 1051 Riverside Drive, New York, New York, 10032; inquiries and training requirements contact posnerk@nyspi.columbia.edu

© 2008 The Research Foundation for Mental Hygiene, Inc.

SUICIDAL IDEATION		
Ask questions 1 and 2. If both are negative, proceed to "Suicidal Behavior" section. If the answer to question 2 is "yes", ask questions 3, 4 and 5. If the answer to question 1 and/or 2 is "yes", complete "Intensity of Ideation" section below.	**Lifetime: Time He/She Felt Most Suicidal**	**Past 1 month**
**1. Wish to be Dead**<br>Subject endorses thoughts about a wish to be dead or not alive anymore, or wish to fall asleep and not wake up.<br>***Have you wished you were dead or wished you could go to sleep and not wake up?***<br>If yes, describe:	Yes ☐ No ☐	Yes ☐ No ☐
**2. Non-Specific Active Suicidal Thoughts**<br>General non-specific thoughts of wanting to end one's life/commit suicide (e.g., "*I've thought about killing myself*") without thoughts of ways to kill oneself/associated methods, intent, or plan during the assessment period.<br>***Have you actually had any thoughts of killing yourself?***<br>If yes, describe:	Yes ☐ No ☐	Yes ☐ No ☐
**3. Active Suicidal Ideation with Any Methods (Not Plan) without Intent to Act**<br>Subject endorses thoughts of suicide and has thought of at least one method during the assessment period. This is different than a specific plan with time, place or method details worked out (e.g., thought of method to kill self but not a specific plan). Includes person who would say, "*I thought about taking an overdose but I never made a specific plan as to when, where or how I would actually do it....and I would never go through with it.*"<br>***Have you been thinking about how you might do this?***<br>If yes, describe:	Yes ☐ No ☐	Yes ☐ No ☐
**4. Active Suicidal Ideation with Some Intent to Act, without Specific Plan**<br>Active suicidal thoughts of killing oneself and subject reports having <u>some intent to act on such thoughts</u>, as opposed to "*I have the thoughts but I definitely will not do anything about them.*"<br>***Have you had these thoughts and had some intention of acting on them?***<br>If yes, describe:	Yes ☐ No ☐	Yes ☐ No ☐
**5. Active Suicidal Ideation with Specific Plan and Intent**<br>Thoughts of killing oneself with details of plan fully or partially worked out and subject has some intent to carry it out.<br>***Have you started to work out or worked out the details of how to kill yourself? Do you intend to carry out this plan?***<br>If yes, describe:	Yes ☐ No ☐	Yes ☐ No ☐
INTENSITY OF IDEATION		
The following features should be rated with respect to the most severe type of ideation (i.e., 1-5 from above, with 1 being the least severe and 5 being the most severe). Ask about time he/she was feeling the most suicidal.<br><u>Lifetime</u> - ***Most Severe Ideation:*** ______ (*Type # (1-5)*) ____________ (*Description of Ideation*)<br><u>Recent</u> - ***Most Severe Ideation:*** ______ (*Type # (1-5)*) ____________ (*Description of Ideation*)	Most Severe	Most Severe
**Frequency**<br>***How many times have you had these thoughts?***<br>(1) Less than once a week (2) Once a week (3) 2-5 times in week (4) Daily or almost daily (5) Many times each day	___	___
**Duration**<br>***When you have the thoughts how long do they last?***<br>(1) Fleeting - few seconds or minutes<br>(2) Less than 1 hour/some of the time<br>(3) 1-4 hours/a lot of time<br>(4) 4-8 hours/most of day<br>(5) More than 8 hours/persistent or continuous	___	___
**Controllability**<br>***Could/can you stop thinking about killing yourself or wanting to die if you want to?***<br>(1) Easily able to control thoughts<br>(2) Can control thoughts with little difficulty<br>(3) Can control thoughts with some difficulty<br>(4) Can control thoughts with a lot of difficulty<br>(5) Unable to control thoughts<br>(0) Does not attempt to control thoughts	___	___
**Deterrents**<br>***Are there things - anyone or anything (e.g., family, religion, pain of death) - that stopped you from wanting to die or acting on thoughts of committing suicide?***<br>(1) Deterrents definitely stopped you from attempting suicide<br>(2) Deterrents probably stopped you<br>(3) Uncertain that deterrents stopped you<br>(4) Deterrents most likely did not stop you<br>(5) Deterrents definitely did not stop you<br>(0) Does not apply	___	___
**Reasons for Ideation**<br>***What sort of reasons did you have for thinking about wanting to die or killing yourself? Was it to end the pain or stop the way you were feeling (in other words you couldn't go on living with this pain or how you were feeling) or was it to get attention, revenge or a reaction from others? Or both?***<br>(1) Completely to get attention, revenge or a reaction from others<br>(2) Mostly to get attention, revenge or a reaction from others<br>(3) Equally to get attention, revenge or a reaction from others and to end/stop the pain<br>(4) Mostly to end or stop the pain (you couldn't go on living with the pain or how you were feeling)<br>(5) Completely to end or stop the pain (you couldn't go on living with the pain or how you were feeling)<br>(0) Does not apply	___	

SUICIDAL BEHAVIOR<br>*(Check all that apply, so long as these are separate events; must ask about all types)*	**Lifetime**	**Past 3 months**
**Actual Attempt:**<br>A potentially self-injurious act committed with at least some wish to die, *as a result of act*. Behavior was in part thought of as method to kill oneself. Intent does not have to be 100%. If there is **any** intent/desire to die associated with the act, then it can be considered an actual suicide attempt. ***There does not have to be any injury or harm***, just the potential for injury or harm. If person pulls trigger while gun is in mouth but gun is broken so no injury results, this is considered an attempt.<br>Inferring Intent: Even if an individual denies intent/wish to die, it may be inferred clinically from the behavior or circumstances. For example, a highly lethal act that is clearly not an accident so no other intent but suicide can be inferred (e.g., gunshot to head, jumping from window of a high floor/story). Also, if someone denies intent to die, but they thought that what they did could be lethal, intent may be inferred.<br>***Have you made a suicide attempt?***<br>***Have you done anything to harm yourself?***<br>***Have you done anything dangerous where you could have died?***<br>***What did you do?***<br>***Did you______ as a way to end your life?***<br>***Did you want to die (even a little) when you_____?***<br>***Were you trying to end your life when you_____?***<br>***Or Did you think it was possible you could have died from_____?***<br>***Or did you do it purely for other reasons / without ANY intention of killing yourself (like to relieve stress, feel better, get sympathy, or get something else to happen)?*** (Self-Injurious Behavior without suicidal intent)<br>If yes, describe:	Yes ☐ No ☐<br><br>Total # of Attempts<br>______	Yes ☐ No ☐<br><br>Total # of Attempts<br>______
**Has subject engaged in Non-Suicidal Self-Injurious Behavior?**	Yes ☐ No ☐	Yes ☐ No ☐
**Interrupted Attempt:**<br>When the person is interrupted (by an outside circumstance) from starting the potentially self-injurious act *(if not for that, actual attempt would have occurred)*.<br>Overdose: Person has pills in hand but is stopped from ingesting. Once they ingest any pills, this becomes an attempt rather than an interrupted attempt. Shooting: Person has gun pointed toward self, gun is taken away by someone else, or is somehow prevented from pulling trigger. Once they pull the trigger, even if the gun fails to fire, it is an attempt. Jumping: Person is poised to jump, is grabbed and taken down from ledge. Hanging: Person has noose around neck but has not yet started to hang - is stopped from doing so.<br>***Has there been a time when you started to do something to end your life but someone or something stopped you before you actually did anything?***<br>If yes, describe:	Yes ☐ No ☐<br><br>Total # of interrupted<br>______	Yes ☐ No ☐<br><br>Total # of interrupted<br>______
**Aborted or Self-Interrupted Attempt:**<br>When person begins to take steps toward making a suicide attempt, but stops themselves before they actually have engaged in any self-destructive behavior. Examples are similar to interrupted attempts, except that the individual stops him/herself, instead of being stopped by something else.<br>***Has there been a time when you started to do something to try to end your life but you stopped yourself before you actually did anything?***<br>If yes, describe:	Yes ☐ No ☐<br><br>Total # of aborted or self-interrupted	Yes ☐ No ☐<br><br>Total # of aborted or self-interrupted<br>______
**Preparatory Acts or Behavior:**<br>Acts or preparation towards imminently making a suicide attempt. This can include anything beyond a verbalization or thought, such as assembling a specific method (e.g., buying pills, purchasing a gun) or preparing for one's death by suicide (e.g., giving things away, writing a suicide note).<br>***Have you taken any steps towards making a suicide attempt or preparing to kill yourself (such as collecting pills, getting a gun, giving valuables away or writing a suicide note)?***<br>If yes, describe:	Yes ☐ No ☐	Yes ☐ No ☐
**Suicidal Behavior:**<br>Suicidal behavior was present during the assessment period?	Yes ☐ No ☐	Yes ☐ No ☐

	Most Recent Attempt<br>Date:	Most Lethal Attempt<br>Date:	Initial/First Attempt<br>Date:
**Actual Lethality/Medical Damage:**<br>0. No physical damage or very minor physical damage (e.g., surface scratches).<br>1. Minor physical damage (e.g., lethargic speech; first-degree burns; mild bleeding; sprains).<br>2. Moderate physical damage; medical attention needed (e.g., conscious but sleepy, somewhat responsive; second-degree burns; bleeding of major vessel).<br>3. Moderately severe physical damage; *medical* hospitalization and likely intensive care required (e.g., comatose with reflexes intact; third-degree burns less than 20% of body; extensive blood loss but can recover; major fractures).<br>4. Severe physical damage; *medical* hospitalization with intensive care required (e.g., comatose without reflexes; third-degree burns over 20% of body; extensive blood loss with unstable vital signs; major damage to a vital area).<br>5. Death	*Enter Code*<br><br>______	*Enter Code*<br><br>______	*Enter Code*<br><br>______
**Potential Lethality: Only Answer if Actual Lethality=0**<br>Likely lethality of actual attempt if no medical damage (the following examples, while having no actual medical damage, had potential for very serious lethality: put gun in mouth and pulled the trigger but gun fails to fire so no medical damage; laying on train tracks with oncoming train but pulled away before run over).<br><br>0 = Behavior not likely to result in injury<br>1 = Behavior likely to result in injury but not likely to cause death<br>2 = Behavior likely to result in death despite available medical care	*Enter Code*<br><br>______	*Enter Code*<br><br>______	*Enter Code*<br><br>______

COLUMBIA-SUICIDE SEVERITY RATING SCALE (C-SSRS)

Since Last Visit

Version 1/14/09

Posner, K.; Brent, D.; Lucas, C.; Gould, M.; Stanley, B.; Brown, G.; Fisher, P.; Zelazny, J.; Burke, A.; Oquendo, M.; Mann, J.

Disclaimer:

This scale is intended to be used by individuals who have received training in its administration. The questions contained in the Columbia-Suicide Severity Rating Scale are suggested probes. Ultimately, the determination of the presence of suicidal ideation or behavior depends on the judgment of the individual administering the scale.

Definitions of behavioral suicidal events in this scale are based on those used in **The Columbia Suicide History Form**, *developed by John Mann, MD and Maria Oquendo, MD, Conte Center for the Neuroscience of Mental Disorders (CCNMD), New York State Psychiatric Institute, 1051 Riverside Drive, New York, NY, 10032. (Oquendo M. A., Halberstam B. & Mann J. J., Risk factors for suicidal behavior: utility and limitations of research instruments. In M.B. First [Ed.]* Standardized Evaluation in Clinical Practice, *pp. 103 -130, 2003.)*

For reprints of the C-SSRS contact Kelly Posner, Ph.D., New York State Psychiatric Institute, 1051 Riverside Drive, New York, New York, 10032; inquiries and training requirements contact posnerk@nyspi.columbia.edu

© 2008 The Research Foundation for Mental Hygiene, Inc.

SUICIDAL IDEATION	
Ask questions 1 and 2. If both are negative, proceed to "Suicidal Behavior" section. If the answer to question 2 is "yes", ask questions 3, 4 and 5. If the answer to question 1 and/or 2 is "yes", complete "Intensity of Ideation" section below.	Since Last Visit
**1. Wish to be Dead**<br>Subject endorses thoughts about a wish to be dead or not alive anymore, or wish to fall asleep and not wake up.<br>***Have you wished you were dead or wished you could go to sleep and not wake up?***<br><br>If yes, describe:	Yes No<br>☐ ☐
**2. Non-Specific Active Suicidal Thoughts**<br>General, non-specific thoughts of wanting to end one's life/commit suicide (e.g., *"I've thought about killing myself"*) without thoughts of ways to kill oneself/associated methods, intent, or plan during the assessment period.<br>***Have you actually had any thoughts of killing yourself?***<br><br>If yes, describe:	Yes No<br>☐ ☐
**3. Active Suicidal Ideation with Any Methods (Not Plan) without Intent to Act**<br>Subject endorses thoughts of suicide and has thought of at least one method during the assessment period. This is different than a specific plan with time, place or method details worked out (e.g., thought of method to kill self but not a specific plan). Includes person who would say, *"I thought about taking an overdose but I never made a specific plan as to when, where or how I would actually do it....and I would never go through with it."*<br>***Have you been thinking about how you might do this?***<br><br>If yes, describe:	Yes No<br>☐ ☐
**4. Active Suicidal Ideation with Some Intent to Act, without Specific Plan**<br>Active suicidal thoughts of killing oneself and subject reports having <u>some intent to act on such thoughts</u>, as opposed to *"I have the thoughts but I definitely will not do anything about them."*<br>***Have you had these thoughts and had some intention of acting on them?***<br><br>If yes, describe:	Yes No<br>☐ ☐
**5. Active Suicidal Ideation with Specific Plan and Intent**<br>Thoughts of killing oneself with details of plan fully or partially worked out and subject has some intent to carry it out.<br>***Have you started to work out or worked out the details of how to kill yourself? Do you intend to carry out this plan?***<br><br>If yes, describe:	Yes No<br>☐ ☐

INTENSITY OF IDEATION	
The following features should be rated with respect to the most severe type of ideation (i.e., 1-5 from above, with 1 being the least severe and 5 being the most severe).<br><br>***Most Severe Ideation:*** ____ ________________<br>*Type # (1-5)* *Description of Ideation*	Most Severe
**Frequency**<br>***How many times have you had these thoughts?***<br>(1) Less than once a week (2) Once a week (3) 2-5 times in week (4) Daily or almost daily (5) Many times each day	____
**Duration**<br>***When you have the thoughts, how long do they last?***<br>(1) Fleeting - few seconds or minutes<br>(2) Less than 1 hour/some of the time<br>(3) 1-4 hours/a lot of time<br>(4) 4-8 hours/most of day<br>(5) More than 8 hours/persistent or continuous	____
**Controllability**<br>***Could/can you stop thinking about killing yourself or wanting to die if you want to?***<br>(1) Easily able to control thoughts<br>(2) Can control thoughts with little difficulty<br>(3) Can control thoughts with some difficulty<br>(4) Can control thoughts with a lot of difficulty<br>(5) Unable to control thoughts<br>(0) Does not attempt to control thoughts	____
**Deterrents**<br>***Are there things - anyone or anything (e.g., family, religion, pain of death) - that stopped you from wanting to die or acting on thoughts of commiting suicide?***<br>(1) Deterrents definitely stopped you from attempting suicide<br>(2) Deterrents probably stopped you<br>(3) Uncertain that deterrents stopped you<br>(4) Deterrents most likely did not stop you<br>(5) Deterrents definitely did not stop you<br>(0) Does not apply	____
**Reasons for Ideation**<br>***What sort of reasons did you have for thinking about wanting to die or killing yourself? Was it to end the pain or stop the way you were feeling (in other words you couldn't go on living with this pain or how you were feeling) or was it to get attention, revenge or a reaction from others? Or both?***<br>(1) Completely to get attention, revenge or a reaction from others<br>(2) Mostly to get attention, revenge or a reaction from others<br>(3) Equally to get attention, revenge or a reaction from others and to end/stop the pain<br>(4) Mostly to end or stop the pain (you couldn't go on living with the pain or how you were feeling)<br>(5) Completely to end or stop the pain (you couldn't go on living with the pain or how you were feeling)<br>(0) Does not apply	____

Version 1/14/09

SUICIDAL BEHAVIOR<br>*(Check all that apply, so long as these are separate events; must ask about all types)*	Since Last Visit
**Actual Attempt:**<br>A potentially self-injurious act committed with at least some wish to die, *as a result of* act. Behavior was in part thought of as method to kill oneself. Intent does not have to be 100%. If there is **any** intent/desire to die associated with the act, then it can be considered an actual suicide attempt. ***There does not have to be any injury or harm***, just the potential for injury or harm. If person pulls trigger while gun is in mouth but gun is broken so no injury results, this is considered an attempt.<br>Inferring Intent: Even if an individual denies intent/wish to die, it may be inferred clinically from the behavior or circumstances. For example, a highly lethal act that is clearly not an accident so no other intent but suicide can be inferred (e.g., gunshot to head, jumping from window of a high floor/story). Also, if someone denies intent to die, but they thought that what they did could be lethal, intent may be inferred.<br>***Have you made a suicide attempt?***<br>***Have you done anything to harm yourself?***<br>***Have you done anything dangerous where you could have died?***<br>***What did you do?***<br>***Did you______ as a way to end your life?***<br>***Did you want to die (even a little) when you_____?***<br>***Were you trying to end your life when you _____?***<br>***Or did you think it was possible you could have died from_____?***<br>***Or did you do it purely for other reasons / without ANY intention of killing yourself (like to relieve stress, feel better, get sympathy, or get something else to happen)?*** (Self-Injurious Behavior without suicidal intent)<br>If yes, describe:<br>**Has subject engaged in Non-Suicidal Self-Injurious Behavior?**	Yes No<br>☐ ☐<br>Total # of Attempts<br>_____<br>Yes No<br>☐ ☐
**Interrupted Attempt:**<br>When the person is interrupted (by an outside circumstance) from starting the potentially self-injurious act *(if not for that, actual attempt would have occurred).*<br>Overdose: Person has pills in hand but is stopped from ingesting. Once they ingest any pills, this becomes an attempt rather than an interrupted attempt. Shooting: Person has gun pointed toward self, gun is taken away by someone else, or is somehow prevented from pulling trigger. Once they pull the trigger, even if the gun fails to fire, it is an attempt. Jumping: Person is poised to jump, is grabbed and taken down from ledge. Hanging: Person has noose around neck but has not yet started to hang - is stopped from doing so.<br>***Has there been a time when you started to do something to end your life but someone or something stopped you before you actually did anything?***<br>If yes, describe:	Yes No<br>☐ ☐<br>Total # of interrupted<br>_____
**Aborted or Self-Interrupted Attempt:**<br>When person begins to take steps toward making a suicide attempt, but stops themselves before they actually have engaged in any self-destructive behavior. Examples are similar to interrupted attempts, except that the individual stops him/herself, instead of being stopped by something else.<br>***Has there been a time when you started to do something to try to end your life but you stopped yourself before you actually did anything?***<br>If yes, describe:	Yes No<br>☐ ☐<br>Total # of aborted or self-interrupted<br>_____
**Preparatory Acts or Behavior:**<br>Acts or preparation towards imminently making a suicide attempt. This can include anything beyond a verbalization or thought, such as assembling a specific method (e.g., buying pills, purchasing a gun) or preparing for one's death by suicide (e.g., giving things away, writing a suicide note).<br>***Have you taken any steps towards making a suicide attempt or preparing to kill yourself (such as collecting pills, getting a gun, giving valuables away or writing a suicide note)?***<br>If yes, describe:	Yes No<br>☐ ☐
**Suicidal Behavior:**<br>Suicidal behavior was present during the assessment period?	Yes No<br>☐ ☐
**Suicide:**	Yes No<br>☐ ☐
Answer for Actual Attempts Only	Most Lethal Attempt Date:
**Actual Lethality/Medical Damage:**<br>0. No physical damage or very minor physical damage (e.g., surface scratches).<br>1. Minor physical damage (e.g., lethargic speech; first-degree burns; mild bleeding; sprains).<br>2. Moderate physical damage; medical attention needed (e.g., conscious but sleepy, somewhat responsive; second-degree burns; bleeding of major vessel).<br>3. Moderately severe physical damage; *medical* hospitalization and likely intensive care required (e.g., comatose with reflexes intact; third-degree burns less than 20% of body; extensive blood loss but can recover; major fractures).<br>4. Severe physical damage; *medical* hospitalization with intensive care required (e.g., comatose without reflexes; third-degree burns over 20% of body; extensive blood loss with unstable vital signs; major damage to a vital area).<br>5. Death	*Enter Code*<br>_____
**Potential Lethality: Only Answer if Actual Lethality=0**<br>Likely lethality of actual attempt if no medical damage (the following examples, while having no actual medical damage, had potential for very serious lethality: put gun in mouth and pulled the trigger but gun fails to fire so no medical damage; laying on train tracks with oncoming train but pulled away before run over).<br>0 = Behavior not likely to result in injury<br>1 = Behavior likely to result in injury but not likely to cause death<br>2 = Behavior likely to result in death despite available medical care	*Enter Code*<br>_____

Appendix M (Initial Gameplay Observations)

**EVO Gameplay**<br><br>**Completed by Study Staff on**<br><br>**DAY 0**<br><br><br>**Participant #:______**	Did the participant understand the task by the time the EVO practice phase was over? **YES / NO (circle)**<br><br><br>Did the participant understand the task by the time the EVO assessment was over? **YES / NO (circle)**<br><br><br>Did the participant seem to enjoy the EVO assessment?<br>**YES / NO (circle)**<br><br><br>Did the participant seem excited to take the EVO game home?<br>**YES / NO (circle)**<br><br><br>Please use the below space to capture your observations:<br>*(observations can include participant reactions, quotes, body language, or other behaviors that you think are relevant)*

Appendix N (Parent/Guardian Questionnaire)

**EVO Gameplay**<br><br>**Completed by Parent on**<br><br>**DAY 28**<br><br>**Participant #:______**	How would you rate your child's enjoyment while playing EVO?<br>**1 2 3 4 5 6 7 8 9 10 (circle)**<br>**Boring OK Fun**<br><br>How challenging do you think EVO was for your child to play?<br>**1 2 3 4 5 6 7 8 9 10 (circle)**<br>**Easy Moderate Hard**<br><br>How would you rate the time your child spent playing EVO?<br>**WASTE OF TIME / SOMEWHAT WORTHWHILE / VERY WORTHWHILE (circle)**<br><br>Did your child exhibit any frustration with the game? **YES / NO (circle)**<br>Please explain:<br><br>Did you notice any real-world improvements in your child's behavior? **YES / NO (circle)**<br>What, specifically?<br><br>If EVO was available to you after this study, would you want him/her to play it more? **YES / NO (circle)**<br><br>Would you want your child to play EVO instead of the regular game he/she plays? **YES / NO / MAYBE (circle)**<br><br>What would have made the experience more enjoyable for your child?<br><br>For you?

**EVO Gameplay**<br><br>**Completed by Parent on**<br><br>**DAY 28**<br><br><br>**Participant #:______**	How often does your child play video games?<br>**NEVER / ONCE A MONTH / ONCE A WEEK / EVERY DAY (circle)**<br><br><br>How many hours a week does your child play?<br>**Less than 1 HOUR / 1 – 5 HOURS / 5 – 10 HOURS Greater than 10 HOURS (circle)**<br><br><br>What types of games does your child like to play?<br>**CONSOLE / PHONE / TABLET / COMPUTER / OTHER (describe) (circle)**<br><br><br>What are some of his/her favorite games?<br><br><br>What further information are you interested in that would make you want to have your child play EVO more?
